# GM‐CSF suppresses antioxidant signaling and drives IL‐1β secretion through NRF2 downregulation

**DOI:** 10.15252/embr.202154226

**Published:** 2022-06-13

**Authors:** Sara Di Carlo, Georg Häcker, Ian E Gentle

**Affiliations:** ^1^ Institute of Medical Microbiology and Hygiene Faculty of Medicine Medical Center – University of Freiburg Freiburg Germany; ^2^ BIOSS Centre for Biological Signalling Studies University of Freiburg Freiburg Germany

**Keywords:** GM‐CSF, IL‐1β, inflammasome, NRF2, TNF, Immunology, Post-translational Modifications & Proteolysis, Signal Transduction

## Abstract

GM‐CSF is a potent inflammatory cytokine regulating myeloid cell differentiation, hematopoiesis, and various other functions. It is functionally associated with a number of inflammatory pathologies including rheumatoid arthritis and inflammatory bowel disease. GM‐CSF has been found to promote NLRP3‐dependent IL‐1β secretion, which may have a significant role in driving inflammatory pathologies. However, the molecular mechanisms remain unknown. Here, we show that GM‐CSF induces IL‐1β secretion through a ROS‐dependent pathway. TNF is required for reactive oxygen species (ROS) generation that strikingly does not promote NLRP3 activation, but instead drives ubiquitylation of IL‐1β, promoting its cleavage through basal NRLP3 activity. GM‐CSF regulates this pathway through suppression of antioxidant responses via preventing upregulation of NRF2. Thus, the pro‐inflammatory effect of GM‐CSF on IL‐1β is through suppression of antioxidant responses, which leads to ubiquitylation of IL‐1β and enhanced processing. This study highlights the role of metabolic regulation of inflammatory signaling and reveals a novel mechanism for GM‐CSF to promote inflammation.

## Graphical abstract

GM‐CSF is a potent cytokine involved in inflammatory pathologies that promotes IL‐1β secretion by blocking antioxidant responses, leading to IL‐1β ubiquitylation. IL‐1β is then processed and secreted from living cells in a NLRP3‐ and gasdermin D‐dependent way.

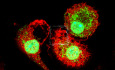

## Introduction

GM‐CSF is a member of the colony‐stimulating factor (CSF) superfamily. Most of these factors play a role in myelopoiesis (Becher *et al*, 2016). Although GM‐CSF is able to trigger differentiation of myeloid precursors, there is debate about the degree to which it is involved in normal hemopoiesis. Loss of GM‐CSF signaling largely affects the differentiation of alveolar macrophages and certain dendritic cell subsets (Stanley *et al*, 1994; Greter *et al*, 2012; Guilliams *et al*, 2013; Schneider *et al*, 2014; Becher *et al*, 2016; Hamilton, 2019). GM‐CSF does, however, play a major role in regulating inflammatory responses of myeloid cells (Hamilton, 2019). GM‐CSF treatment of monocytes drives the differentiation of monocytic macrophages and dendritic cells (Erlich *et al*, 2019) and can promote the inflammatory activity of macrophages (Na *et al*, 2016; Hamilton, 2019; Lee *et al*, 2019). GM‐CSF is mostly produced by lymphocytes at sites of inflammation but can also be secreted by fibroblasts and endothelial and epithelial cells (Hamilton, 2019). Through activation of myeloid cells, it is thought that GM‐CSF can contribute to inflammatory feedback loops in concert with other factors such as TNF and IL‐1β (Hamilton, 1993). This has implications in pathologies such as rheumatoid arthritis where GM‐CSF plays an important role and anti‐GM‐CSF therapies have recently been shown to be beneficial (Lee *et al*, 2020).

IL‐1β is an inflammatory cytokine that plays a central role in many inflammatory pathologies. The regulation of its secretion is unconventional in that IL‐1β is produced in an immature pro‐form that must be cleaved by caspase‐1 within inflammasome complexes to produce the mature secreted protein. Inflammasomes are protein platforms for caspase‐1 recruitment and activation and consist of different receptor proteins that recognize various ligands and that recruit the adaptor ASC, followed by caspase‐1 (Schroder & Tschopp, 2010; Broz & Dixit, 2016). NLRP3 is the best‐studied receptor, although it is unknown exactly what NLRP3 recognizes, as many stimuli can trigger its multimerization. Secretion of IL‐1β via NLRP3 activity requires a two‐step mechanism involving a priming step driven by TLR activation, often TLR4, that upregulates IL‐1β synthesis as well as various post‐translational modifications of inflammasome components (McKee & Coll, 2020). The second step is provided by activators that typically trigger potassium efflux including activation of P2X7 receptors (ATP) or ionophores such as nigericin. These secondary stimuli lead to the assembly of NLRP3 inflammasomes and caspase‐1 activation. However, other stimuli also activate NLRP3 and induce IL‐1β secretion independently of potassium efflux, including the poisoning of the mitochondrial respiratory chain through imiquimod, or depletion of NADH pools by salmonella infection (Groß *et al*, 2016; Sanman *et al*, 2016; Yabal *et al*, 2018). Many studies have now linked mitochondrial dysfunction to NLRP3 activation, particularly through the generation of reactive oxygen species (ROS) (Yabal *et al*, 2018; Paik *et al*, 2021). Both steps in priming and also activation of NRLP3 inflammasomes are suggested to be regulated by ROS, depending on the stimulus.

Upon experimental inflammasome activation, cells die rapidly through a process called pyroptosis, caused by caspase‐1‐dependent cleavage of gasdermin D, which assembles into large pores in the plasma membrane, leading to leakage of the cytoplasm and cell death (He *et al*, 2015; Liu *et al*, 2016). It has been proposed that this process is required for the release of IL‐1β, but other studies have since shown that there can be gasdermin D‐dependent IL‐1β release in the absence of cell death (Evavold *et al*, 2018; Monteleone *et al*, 2018). This cell death‐independent IL‐1β secretion is typically slower than during pyroptosis (Monteleone *et al*, 2018). GM‐CSF‐dependent promotion of IL‐1β secretion can occur through NLRP3 (Budai *et al*, 2017). We, here, use primary monocytic macrophages and HoxB8 macrophages (Wang *et al*, 2006; Rosas *et al*, 2011) to show that GM‐CSF causes NLRP3‐dependent IL‐1β secretion that is independent of cell death and is driven by mitochondrial ROS production and post‐translational modification of IL‐1β. We found that GM‐CSF promoted the activity of ROS through suppression of NRF2 and downstream antioxidant responses, identifying this axis as an important mediator of the inflammatory activity of this cytokine.

## Results

### GM‐CSF promotes NLRP3‐dependent IL‐1β secretion in response to LPS

**Figure 1:**
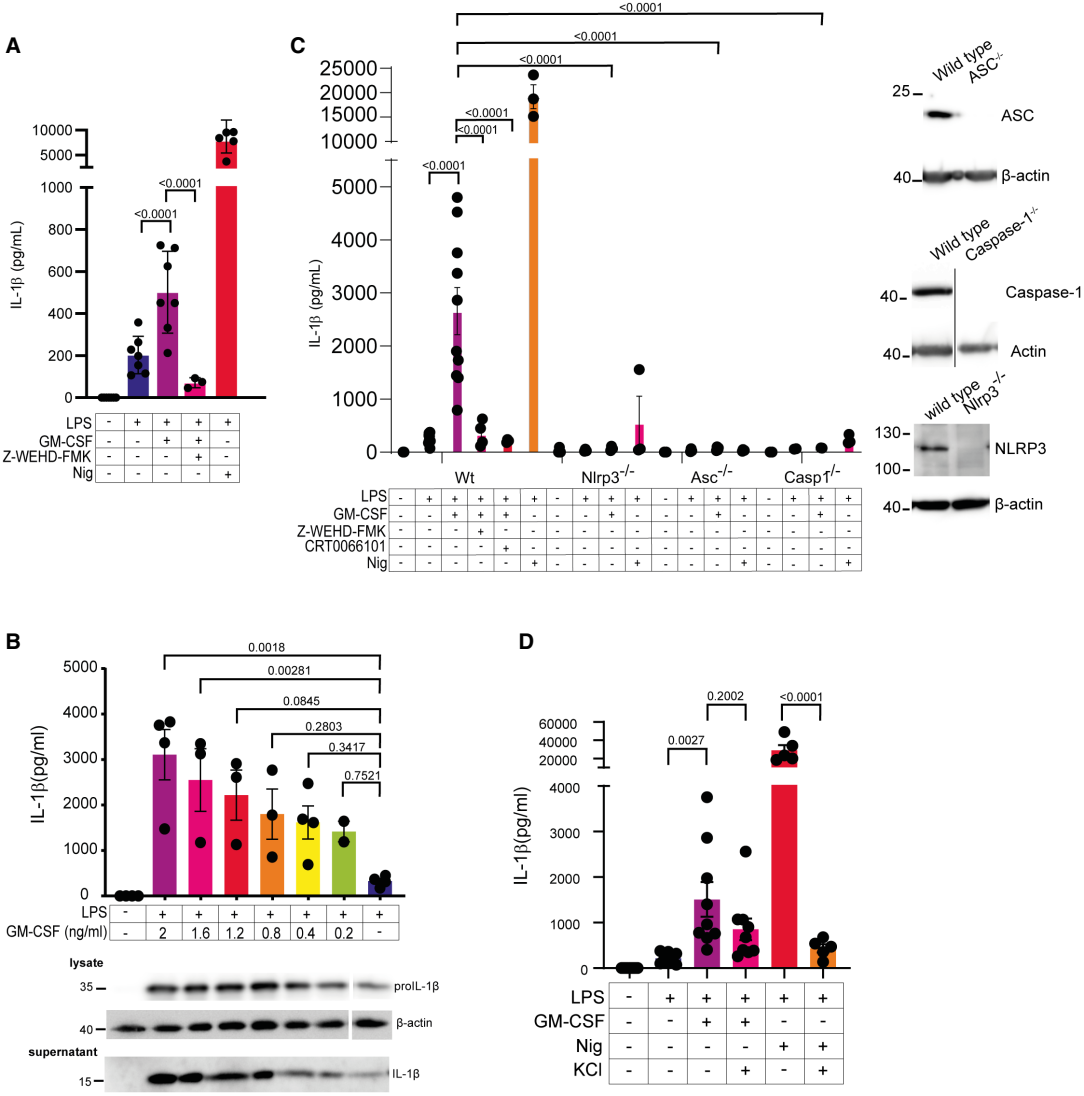
GM‐CSF induces NLRP3‐dependent IL‐1β secretion BMDMM were treated as indicated with either 60 ng/ml ultra‐pure (UP) LPS, 50 ng/ml human‐derived recombinant GM‐CSF, 40 µM WEHD‐FMK for 16 h, or 5 µM nigericin for the last hour. Supernatants were then analyzed for IL‐1β secretion by ELISA.HoxB8 macrophages were treated with the indicated dilutions of GM‐CSF using supernatants from GM‐CSF‐expressing cells. Shown are ELISA analysis of IL‐1β secretion in supernatants and western blots indicating IL‐1β expression in lysates.HoxB8 macrophages of the indicated genotypes were treated as indicated with 60 ng/ml UP‐LPS, 1% GM‐CSF containing supernatant (approximately 2 ng/ml GM‐CSF), 40 µM WEHD.FMK, and 0.5 µM CRT‐0066101 for 16 h; 5 µM nigericin was added for the last hour. Supernatants were analyzed by ELISA for IL‐1β. Inlay panels show western blots for levels of IL‐1β expression in the indicated knockouts.HoxB8 cells were treated with 60 ng/ml of UP‐LPS, 1% GM‐CSF supernatant, 50 mM KCl, and 5 µM nigericin as indicated for 16 h before supernatants were analyzed for IL‐1β by ELISA. Data information: *n* ≥ 3 biological replicates (every dot represents one biological replicate). For all panels, error bars are SEM. Significance was calculated using one‐way ANOVA with multiple comparisons or two‐way ANOVA to compare across genotypes. *P*‐values are shown. Source data are available online for this figure.

**Figure EV1:**
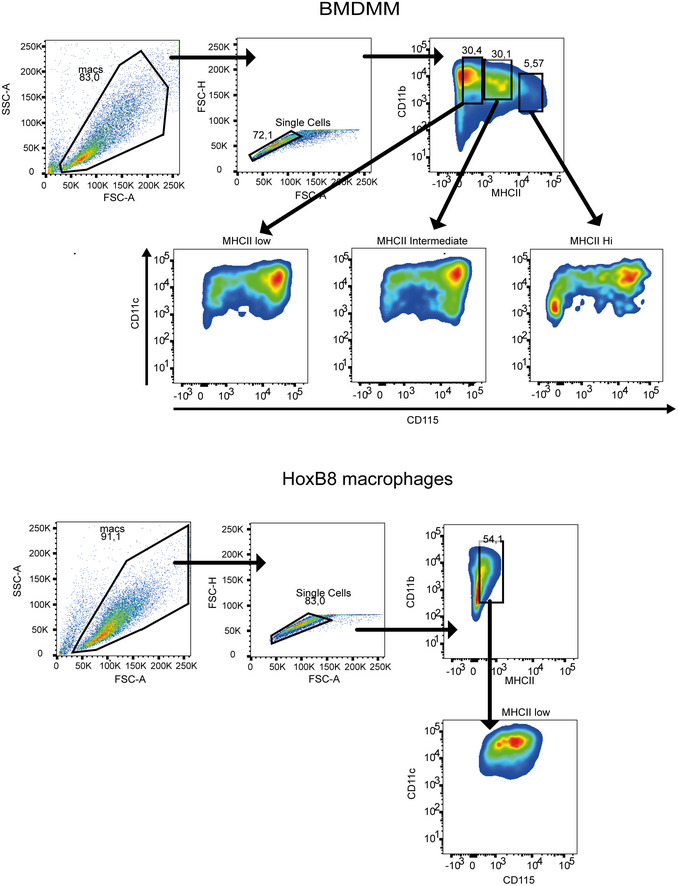
HoxB8 macrophages are similar to bone marrow‐derived monocytic macrophages Shown is the surface staining of markers for monocytic macrophages. All cells were stained as described in the Materials and Methods and staining was detected using flow cytometry. Data information: Staining is representative of *n* ≥ 3 biological replicates.

**Figure EV2:**
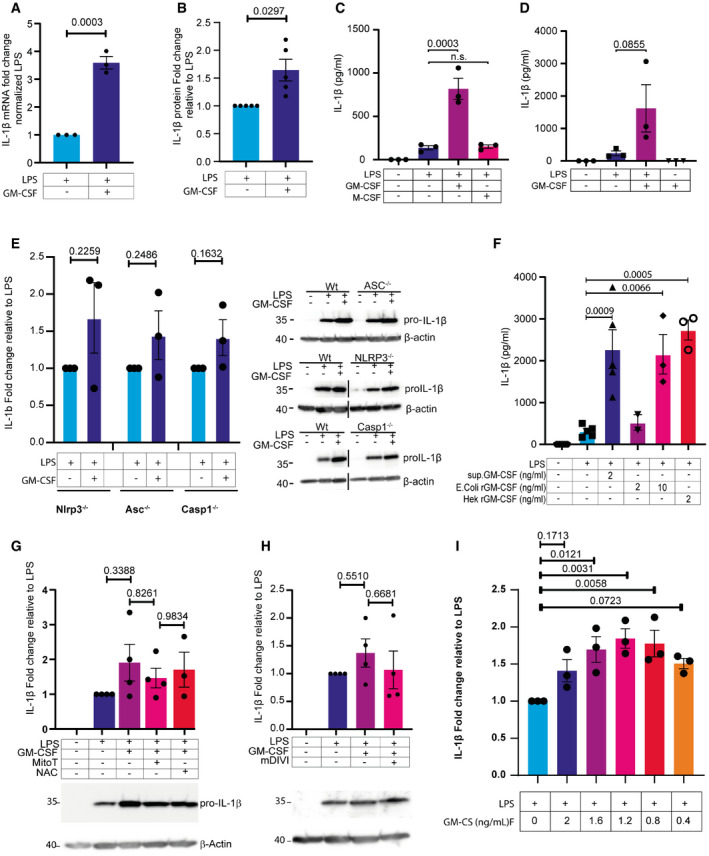
Quantification of IL‐1β mRNA and protein levels HoxB8 macrophages were treated for 12 h as indicated. The RNA was extracted and analyzed by qPCR for IL‐1β.Densitometric quantification of pro‐IL‐1β protein level relative to LPS‐treated samples.HoxB8 macrophages were co‐treated with 10 ng/ml recombinant M‐CSF for 16 h as indicated. Media were analyzed for IL‐1β secretion by ELISA.HoxB8 macrophages were treated as indicated using 60 ng/ml UP‐LPS and or 1% of GM‐CSF supernatant. IL‐1β secretion was analyzed in the supernatant by ELISA.Densitometric quantification of pro‐IL‐1β protein level in the indicated HoxB8 genotypes treated for 16 h with LPS in the presence or not of GM‐CSF. Shown are representative western blots of pro‐IL‐1β (right panel). Black vertical lines indicate non‐relevant lanes that were removed during figure preparation. All samples were run on the same gel.HoxB8 macrophages were treated with the indicated concentrations of either media from GM‐CSF producing cells, *E. coli*‐expressed recombinant GM‐CSF, or HEK293‐expressed recombinant GM‐CSF plus LPS. Supernatants were analyzed for IL‐1 β by ELISA.Densitometric quantification of pro‐IL‐1β protein level relative to LPS‐treated samples. HoxB8 macrophages were treated as indicated for 16 h. Data correspond to Fig. 2A. Below are shown representative western blots.Densitometric quantification of pro‐IL‐1β protein level relative to LPS‐treated samples. HoxB8 macrophages were treated as indicated for 16 h. Data correspond to Fig. 2C. Below are shown representative western blots.Densitometric quantification of pro‐IL‐1β protein level relative to LPS‐treated samples. HoxB8 macrophages were treated with indicated doses of supernatant containing GM‐CSF for 16 h. Data information: *n* ≥ 3 biological replicates (every dot represents one biological replicate). For all panels, error bars are SEM. Significance was calculated using unpaired *t*‐test. *P*‐values are shown. Source data are available online for this figure.

**Figure 2:**
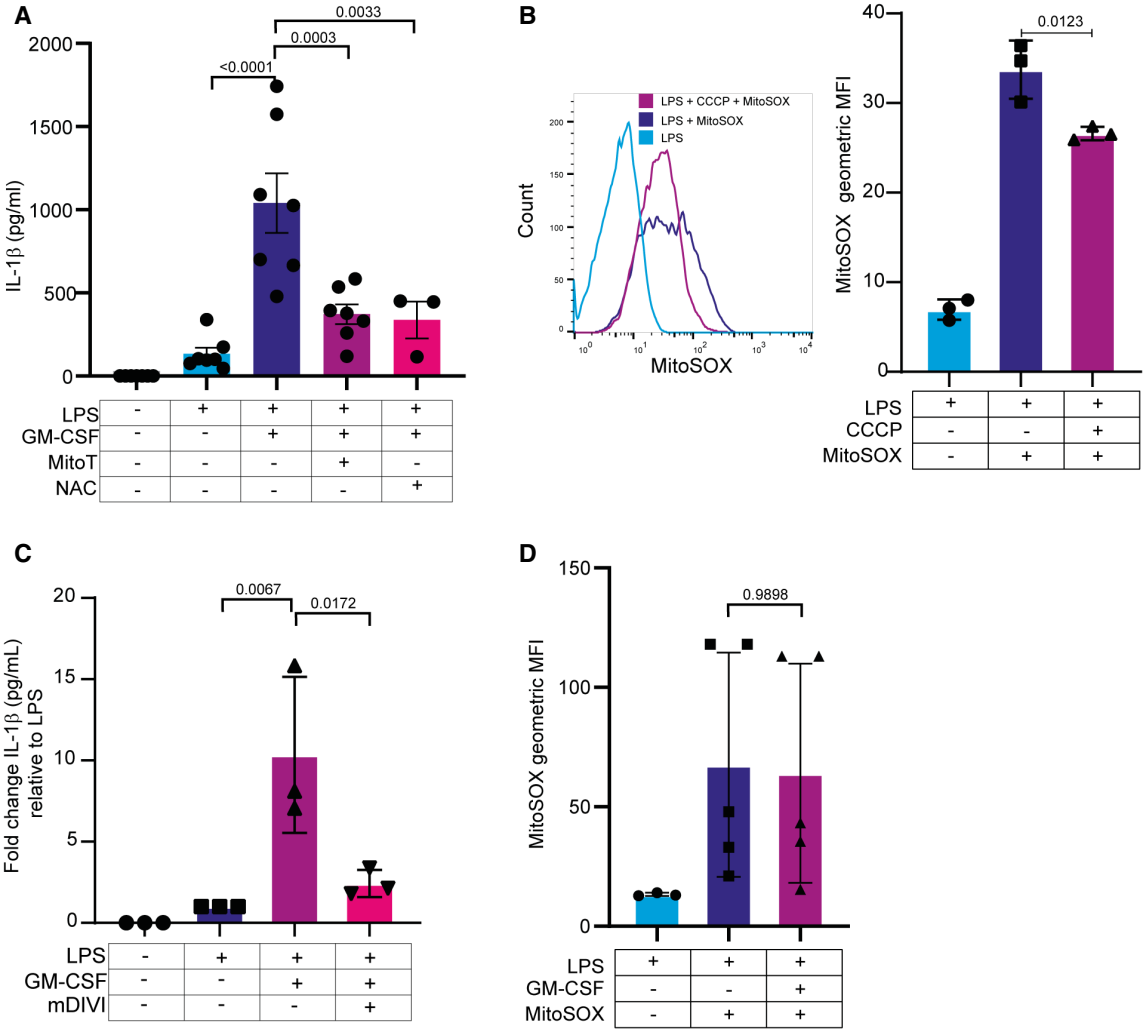
Mitochondrial ROS are required for GM‐CSF‐induced IL‐1β secretion HoxB8 macrophages were treated as indicated with the antioxidant compounds mitoTEMPO or NAC during LPS + GM‐CSF treatment. Levels of secreted IL‐1β were measured by ELISA.HoxB8 macrophages were treated for 8 h with 60 ng/ml of LPS and then stained with mitoSOX red as described in the Materials and Methods. MitoSOX red fluorescence was then measured using flow cytometry. Shown are a representative histogram and the quantification of the mean fluorescence intensity (MFI) from three independent experiments.HoxB8 macrophages were treated with 60 ng/ml of LPS for 16 h with or without 25 µM mDIVI‐1. IL‐1β was measured in the supernatant by ELISA.MFI quantification of mitoSOX staining. Wild‐type cells were treated as in (B) with LPS alone or LPS and GM‐CSF. Data information: *n* ≥ 3 biological replicates (every dot represents one biological replicate). For all panels, error bars are SEM. Significance was tested using one‐way ANOVA with multiple comparisons. *P*‐values are shown.

Monocytic macrophages were recently shown to be the major IL‐1β‐secreting cells in BMDC cultures (Erlich *et al*, 2019). Additionally, monocytes differentiated in GM‐CSF have been reported to secrete IL‐1β without secondary signals (Budai *et al*, 2017). To determine if GM‐CSF can drive IL‐1β secretion in these cells, we generated Bone Marrow‐Derived Monocytic Macrophages (BMDMM) by differentiation of bone marrow in GM‐CSF as described in the Materials and Methods and treated them with LPS or LPS and GM‐CSF. There was a clear increase in IL‐1β secretion in GM‐CSF‐treated cells (Fig 1A). GM‐CSF triggered secretion of IL‐1β without the addition of a typical secondary stimulus, suggesting that it can activate the inflammasome by itself (Fig 1A and B). HoxB8‐immortalized myeloid progenitors can be differentiated in GM‐CSF to provide a useful model for monocytic macrophages (Wang *et al*, 2006; Rosas *et al*, 2011) (Fig EV1). Using HoxB8‐immortalized macrophages, we confirmed that GM‐CSF promotes IL‐1β secretion in a dose‐dependent fashion (Fig 1B). GM‐CSF also enhanced the expression of pro‐IL‐1β at the level of transcription, which has been previously published (Oster *et al*, 1992) (Fig EV2A). However, the increase in pro‐IL‐1β could not account for the around 10‐fold higher levels of secreted IL‐1β (Fig 1B), and quantification of western blots showed only modest upregulation which was not significant (Fig EV2B). M‐CSF is a related cytokine that also promotes the differentiation of myeloid cells into macrophages. We confirmed that M‐CSF is not able to promote IL‐1β secretion like GM‐CSF can (Fig 2C). Additionally, we also confirmed that GM‐CSF treatment alone is unable to trigger IL‐1β secretion but still requires the priming activity of LPS (Fig EV2D).

Because Hoxb8 cells allow more flexibility with genetic manipulations and growth, we chose to use them as a model for further investigation. IL‐1β can be processed by multiple inflammasomes; however, the NLRP3 inflammasome has been reported to be involved in the secretion of IL‐1β in GM‐CSF‐derived myeloid cells previously (He *et al*, 2013; Budai *et al*, 2017), although the mechanism remained unclear. Using HoxB8 macrophage cell lines, we tested NLRP3^−/−^, ASC^−/−^, and Caspase1^−/−^ cells for GM‐CSF‐induced IL‐1β secretion. Loss of NLRP3, caspase‐1, or ASC abolished the effect of GM‐CSF, as did caspase‐1 inhibition using the peptide‐inhibitor WEHD.FMK (Fig 1C). No changes in pro‐IL‐1β production were seen between the knockout cell lines (Fig EV2E). NLRP3 activation was also shown to be dependent on the activity of protein kinase D (PKD) (Zhang *et al*, 2017). IL‐1β secretion could also be blocked by inhibition of PKD by CRT0066101 (Fig 1C). Commonly, K^+^ efflux is involved in the activation of NLRP3 inflammasomes, although several stimuli are known to bypass this requirement. We tested the requirement for K^+^ efflux by incubating HoxB8 macrophages with 50 mM KCl alongside LPS + GM‐CSF (Fig 1D). While a reduction was seen in IL‐1β secretion, it was mild and not significant compared to its effect on nigericin treatment (Fig 1D). This is in line with other findings showing K^+^‐independent NLRP3 activation upon treatments that induce ROS (Groß *et al*, 2016; Sanman *et al*, 2016).

As a technical note, we mostly used supernatants from B16 melanoma cells expressing GM‐CSF or recombinant GM‐CSF produced in HEK293 cells. Up to 10 times higher concentrations of *E. coli*‐derived recombinant GM‐CSF were required to induce IL‐1β secretion (Fig EV2F). This suggests that modifications such as glycosylation are required for GM‐CSF activity. This may be due to the increased half‐life of glycosylated cytokines and the long time courses used in these assays.

### GM‐CSF‐induced IL‐1β secretion requires mitochondrial Reactive Oxygen Species

Given the absence of other obvious secondary activators of NRLP3 in GM‐CSF‐treated cells, this suggests that a cell‐intrinsic signal triggered by GM‐CSF is leading to IL‐1β secretion. Mitochondrial ROS are known regulators of NLRP3 activity that act in a cell‐intrinsic fashion. In order to determine if ROS are required for GM‐CSF‐induced IL‐1β secretion, macrophages were treated with the ROS scavengers NAC and mitoTEMPO prior to stimulation. Both agents blocked secretion of IL‐1β substantially but had little effect on induction of pro‐IL‐1β (Fig 2A), indicating that mitochondrial ROS are indeed required for GM‐CSF‐induced IL‐1β secretion. The mitochondrial origin of the ROS was further confirmed by inhibition of oxidative phosphorylation. LPS‐stimulated macrophages perform reverse electron transport (RET) which generates ROS from complex I (Mills *et al*, 2016) and this should be blocked by complex I inhibitors or other inhibitors such as CCCP that disrupt the electron transport chain. We confirmed this using mitoSOX staining that showed a reduction in mitoSOX fluorescence after CCCP treatment, due to dissipation of high membrane potential which is a requirement for RET to occur (Fig 2B) (Scialò *et al*, 2017). To avoid toxicity from rotenone or other inhibitors over the long time course of the GM‐CSF stimulus, we used mDIVI‐1, a substance developed to inhibit DRP1 on the mitochondrial outer membrane, which has subsequently been shown to be a reversible complex I inhibitor that is tolerated by cells over longer treatments (Bordt *et al*, 2017). Pretreatment of HoxB8 macrophages with mDIVI‐1 fully blocked IL‐1β secretion in response to LPS + GM‐CSF (Fig 2C). We again confirmed that the use of ROS scavengers and mDIVI did not prevent IL‐1β production (Fig EV2G and H). These data suggest that LPS‐stimulated HoxB8 macrophages produce mitochondrial ROS, probably via RET through complex I, and that these ROS are driving IL‐1β secretion. To examine if GM‐CSF may be driving IL‐1β secretion through induction of ROS, we stimulated HoxB8 cells with LPS or LPS + GM‐CSF and stained them with mitoSOX. No differences in mitoSOX staining were observed after GM‐CSF treatment (Fig 2D).

### GM‐CSF‐induced IL‐1β secretion is not due to global changes in cytokine secretion

**Figure EV3:**
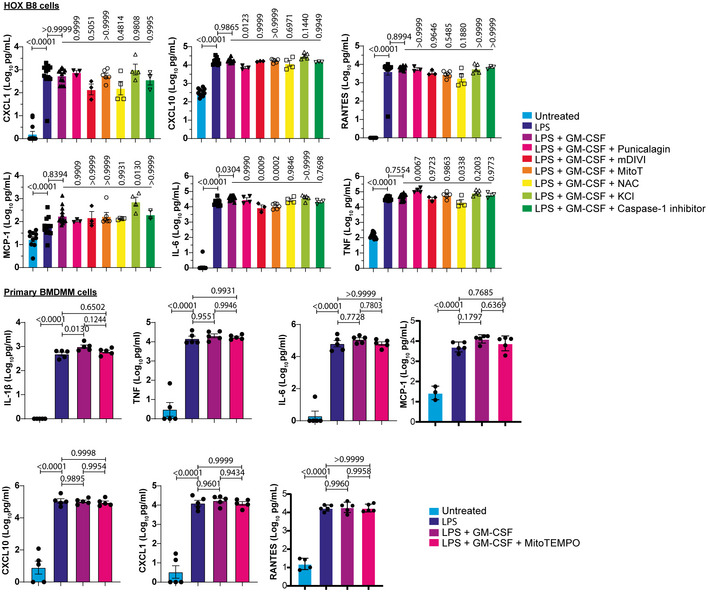
LPS and GM‐CSF treatment induce a pro‐inflammatory profile HoxB8 macrophages or BMDMM were treated for 16 h as indicated. The indicated cytokines were analyzed in supernatants by LEGENDplex antiviral response assay as indicated in the Materials and Methods. Shown are the concentrations (pg/ml) converted into log_10_ in order to be following a normal Gaussian distribution for statistical analysis. Data information: *n* ≥ 3 biological replicates (every dot represents one biological replicate). For all panels, error bars are SEM. Significance was calculated using one‐way ANOVA with multiple comparisons. *P*‐values are indicated.

To determine that there is not simply a global increase in cytokine secretion triggered by GM‐CSF, we performed multiplex ELISA (LEGENDplex) on supernatants from LPS or LPS + GM‐CSF‐treated cells, both HoxB8 and primary cells, with or without the inhibitors described above, and measured levels of CXCL1, CXCL10, IL‐6, TNF, MCP‐1, and RANTES. For primary cells, only, IL‐1β showed significant increases in secretion in response to GM‐CSF, and this could be reversed by mitoTEMPO. Otherwise, no significant changes could be detected in response to GM‐CSF. HoxB8 cells showed a significant increase in IL‐6 and MCP‐1 after GM‐CSF treatment. For IL‐6, this could be reversed by mDIVI or mitoTEMPO treatment (Fig EV3). TNF secretion could also be reduced by NAC treatment but was, unlike IL‐1β, increased by punicalagin. Punicalagin also caused a significant decrease in CXCL10 secretion. Otherwise, all other cytokines were not significantly affected. Thus, IL‐6 is surprisingly the only cytokine that shows a similar pattern of response to IL‐1β. This implies that the effect seen for IL‐1β is rather specific and that the inhibitors we have used are not simply blocking cytokine production in general.

### GM‐CSF‐induced IL‐1β secretion requires TNF but is independent of cell death

**Figure 3:**
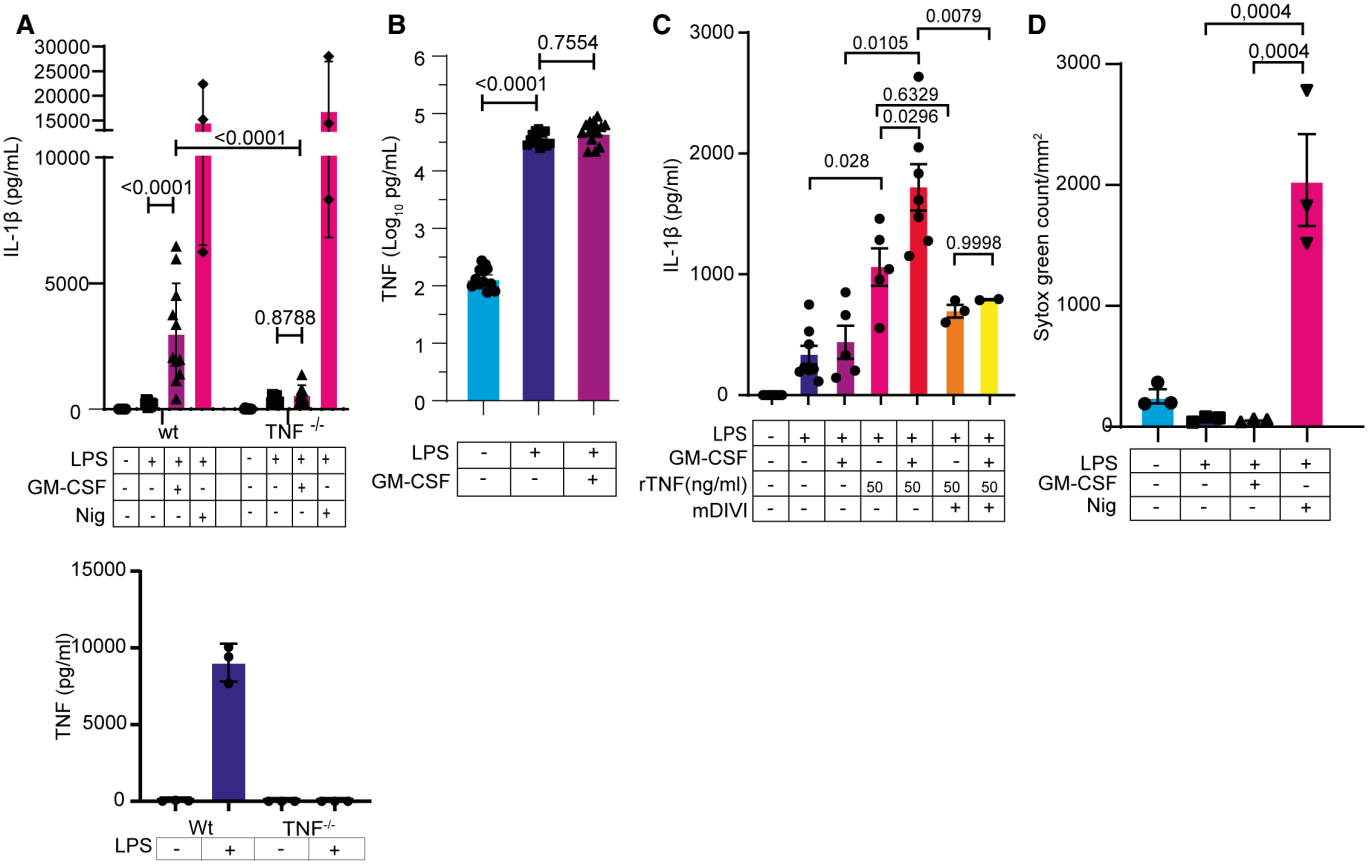
TNF is required for GM‐CSF‐induced IL‐1β secretion, independently of cell death HoxB8 macrophages of the indicated genotypes were treated for 16 h with 60 ng/ml of UP‐LPS with or without GM‐CSF and IL‐1β in the supernatant was analyzed by ELISA. Bottom graph shows by ELISA that TNF^−/−^ cells do not secrete any TNF when treated with UP‐LPS.The same supernatants shown in (A) were measured for TNF by LEGENDplex assay.TNF^−/−^ HoxB8 macrophages were treated as indicated with the addition of 50 ng/ml recombinant TNF throughout the treatment with or without mDIVI for 16 h. Levels of IL‐1β were then measured in the supernatant by ELISA.HoxB8 macrophages were seeded in a 96‐well microplate and treated as indicated. After 16 h, SYTOX green was added and cells were imaged in an IncuCyte incubator microscope. Shown is the SYTOX green signal normalized to the total cell area. Data information: *n* ≥ 3 biological replicates (every dot represents one biological replicate). For all panels, error bars are SEM. Significance was measured using one‐way ANOVA with multiple comparisons, or two‐way ANOVA to compare across genotypes. *P*‐values below 0.1 are shown.

TNF is a known driver of ROS and LPS‐stimulation results in autocrine TNF signaling. To test if TNF was required for IL‐1β secretion, TNF^−/−^ macrophages were also tested and showed a near‐complete loss of secretion of IL‐1β (Fig 3A). Knockout was confirmed with ELISA against TNF (Fig 3A lower panel). No significant differences in TNF secretion were detected in multiplex ELISA samples between LPS and LPS + GM‐CSF‐treated cells (Figs 3B and EV2), showing GM‐CSF is not acting by suppressing TNF production. To test if we could restore the sensitivity to GM‐CSF in TNF^−/−^ HoxB8 macrophages, recombinant TNF was added throughout the treatment with LPS ± GM‐CSF. Multiplex ELISA showed levels of TNF at around 50 ng/ml, so we used this dose to treat TNF^−/−^ cells. The addition of TNF and LPS without GM‐CSF was able to induce IL‐1β secretion and GM‐CSF caused a significant enhancement of this (Fig 3C). The GM‐CSF‐induced increase could, in turn, be reversed by the addition of mDIVI, confirming it to be a ROS‐driven event. Cell death through pyroptosis is associated with IL‐1β release, and TNF may promote cell death in macrophages. To control for this, cell cultures were assayed for cell death by SYTOX green exclusion using live‐cell imaging. Almost no cell death was observed, and no increase in cell death was observed in GM‐CSF‐treated cells compared to LPS‐only treated cells (Fig 3D). GM‐CSF clearly does not induce cell death in order to promote the secretion of IL‐1β.

### GM‐CSF‐induced IL‐1β secretion is blocked by IL‐10 treatment, but GM‐CSF does not affect the upregulation of IRG1

**Figure EV4:**
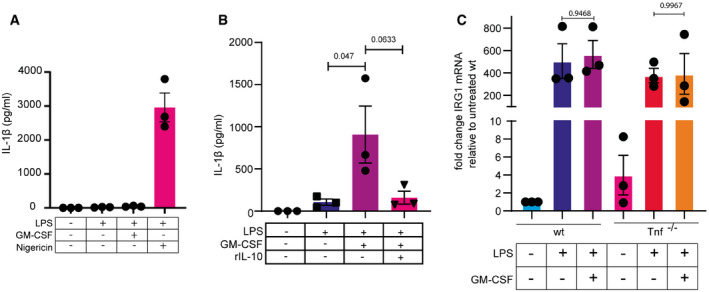
GM‐CSF‐induced IL‐1β secretion is blocked by IL‐10 but does not require IRG1 dysregulation BMDM were generated as described in Materials and Methods and treated as indicated for 16 h. IL‐1β levels were measured in media by ELISA.HoxB8 macrophages were treated as indicated with or without recombinant IL‐10 and secreted IL‐1β levels were measured using ELISA.Wild‐type and Tnf^−/−^ HoxB8 macrophages were treated as indicated for 12 h, and RNA was extracted. Levels of IRG1 were analyzed by qPCR. Data information: *n* ≥ 3 biological replicates (every dot represents one biological replicate). For all panels, error bars are SEM. Significance was calculated using two‐way ANOVA with multiple comparisons. *P*‐values are shown.

We were unable to trigger IL‐1β secretion with LPS + GM‐CSF in bone marrow‐derived macrophages (BMDM) (Fig EV4A). BMDM are known to be functionally distinct from bone marrow‐derived monocytic macrophages, particularly with respect to the production of IL‐10. BMDM produce significant amounts of IL‐10 upon LPS stimulation, whereas monocytic macrophages do not (Lacey *et al*, 2012; Budai *et al*, 2017). Loss of IL‐10 or its cognate receptor results in IL‐1β secretion in response to LPS in BMDM (Ip *et al*, 2017). That study also demonstrated a requirement for ROS to trigger IL‐1β secretion. IL‐10 can also block cleavage and secretion of IL‐1β in tissue‐resident macrophages (Ipseiz *et al*, 2020). In order to determine if IL‐10 could reverse the function of GM‐CSF in triggering IL‐1β secretion, we treated wild‐type Hoxb8 macrophages with IL‐10 in combination with LPS + GM‐CSF. IL‐10 strongly suppressed IL‐1β secretion (Fig EV4B).

Mitochondrial ROS generation is largely driven by the electron transport chain, particularly through complexes I and III. Recent studies have demonstrated that in macrophages treated with LPS, oxidative phosphorylation is blocked through the upregulation of IRG1 (Immune‐Responsive Gene 1), which is responsible for the generation of the metabolite Itaconate. Itaconate has multiple effects on inflammasome activation, antioxidant responses, and electron transport chain activity, where it blocks the activity of complex II (Mills *et al*, 2016, 2018; Hooftman *et al*, 2020). IL‐10 was also reported to block Irg1 expression in macrophages in response to bacterial exposure (Gautam *et al*, 2011; De Souza *et al*, 2019). To test if the expression of IRG1 could be regulating oxidative phosphorylation and ROS production, we tested its mRNA expression during treatment with LPS or LPS + GM‐CSF. Basal expression of IRG1 was very low, but upon LPS stimulation, massive upregulation was observed. IRG1 expression between LPS and LPS + GM‐CSF was comparable, however (Fig EV4C). Additionally TNF^−/−^ macrophages, although having higher basal IRG1, also showed no significant change in IRG1 expression in response to GM‐CSF. Therefore, IRG1 expression is not behind GM‐CSF‐TNF‐driven IL‐1β secretion and is unlikely to be the cause of IL‐10‐dependent blockage of IL‐1β secretion.

### GM‐CSF prevents NRF2 upregulation and cellular responses to oxidative stress induced by LPS

**Figure 4:**
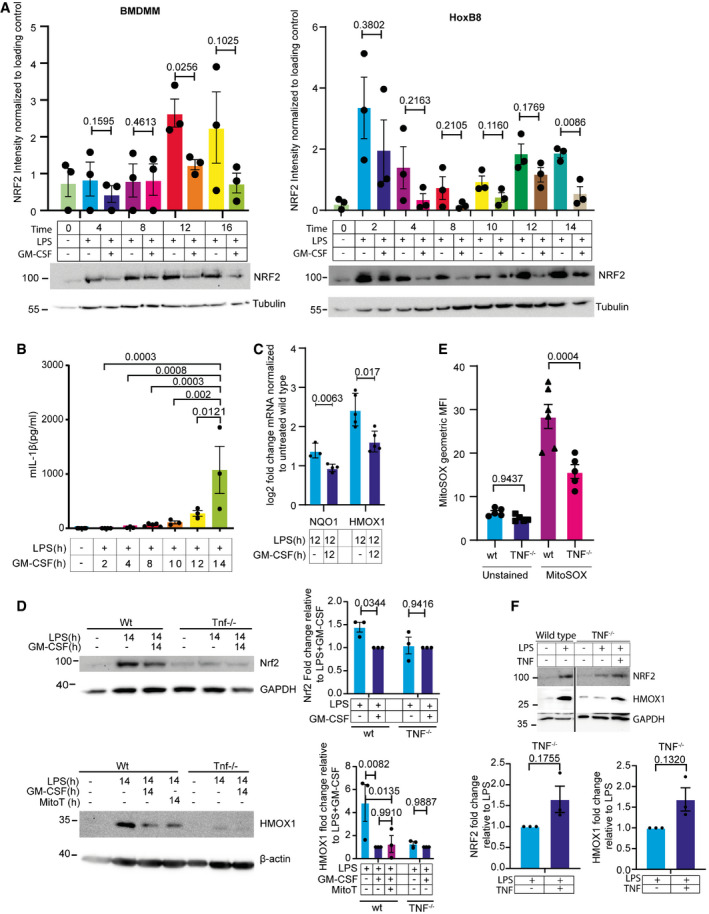
GM‐CSF suppresses NRF2 upregulation to promote ROS activity BMDMM (left panel) and HoxB8 macrophages (right panel) were treated as indicated with either LPS or LPS + GM‐CSF. Cells were harvested and proteins extracted and analyzed by western blot for levels of NRF2. Shown is the densitometric quantification of Nrf2 protein level from three independent experiments.HoxB8 macrophages were treated as in (A). and levels of IL‐1β in the supernatant were analyzed by ELISA.HoxB8 macrophages were treated for 12 h with LPS or LPS + GM‐CSF and RNA was extracted and analyzed by qPCR for the indicated targets.Wild‐type and TNF^−/−^ HoxB8 cells were treated as indicated. Cells were harvested and proteins extracted and analyzed by western blot for levels of NRF2 and HMOX1. For wild‐type cells, MitoTEMPO (50 µM) was also added as indicated. Shown are the densitometric quantifications of NRF2 and HMOX1 protein levels of three independent experiments (right panel).HoxB8 cells of the indicated genotypes were treated with LPS for 8 h, followed by staining with mitoSOX red. The fluorescent signal was analyzed using flow cytometry. Shown is the average geometric mean fluorescence intensity of mitoSOX red.TNF^−/−^ HoxB8 macrophages were treated with LPS with or without 50 ng/ml recombinant TNF for 16 h. Cells were harvested and proteins extracted and analyzed by western blot for levels of NRF2 and HMOX1 (upper panel). Shown are the densitometric quantifications of indicated protein levels of three independent experiments (lower panel). Data information: *n* ≥ 3 biological replicates (every dot represents one biological replicate). For all panels, error bars are SEM. Significance was calculated using one‐way ANOVA with multiple comparisons, or two‐way ANOVA to compare across genotypes. For the western blot quantification, significance was calculated using unpaired *t*‐test. *P*‐values are shown. Source data are available online for this figure.

Although ROS appear to drive the secretion of IL‐1β, GM‐CSF‐treated cells did not show increased ROS staining (Fig 2D). One possible, alternative explanation for the GM‐CSF effect could be that GM‐CSF‐treated cells lack the capacity to counter ROS signaling efficiently through changes in the antioxidant response. To test if the normal redox stress response is active in GM‐CSF‐treated cells, we analyzed the levels of NRF2 over time in BMDMM and in HoxB8 macrophages treated with LPS or LPS + GM‐CSF. NRF2 is a master regulator of antioxidant responses and acts as a ROS‐sensitive transcription factor to drive the upregulation of various antioxidant genes (Saha *et al*, 2020). LPS induced a robust NRF2 upregulation in BMDMM, which was sustained throughout the 14 h treatment. In HoxB8 cells, the NRF2 response showed two waves of induction: a strong increase in the first 2 h was followed by a reduction and the second wave of NRF2 upregulation between 10 and 14 h in HoxB8 cells (Fig 4A). Strikingly, in cells co‐treated with GM‐CSF, NRF2 upregulation was reduced in both primary BMDMM and HoxB8 cells (Fig 4A). The time at which NRF2 was reduced and then returned varied but is significantly different at 14 h in HoxB8 cells and 12 h in the BMDMM (Fig 4A). The timing of the second NRF2 wave in HoxB8 cells coincided with the appearance of IL‐1β secretion (Fig 4B). A reduction of NRF2 activity was confirmed by qPCR against the NRF2 targets Nqo1 and HMOX1, which both showed a significant reduction in mRNA levels in GM‐CSF‐treated cells (Fig 4C), as well as by western blot against HMOX‐1, whose LPS‐induced upregulation was reduced by GM‐CSF treatment (Fig 4D). The ROS‐scavenger mitoTEMPO also blocked the upregulation of HMOX‐1 (Fig 4D).

In TNF^−/−^ cells, GM‐CSF had been unable to induce IL‐1β secretion. TNF can trigger ROS production (Schulze‐Osthoff *et al*, 1992; Blaser *et al*, 2016). We, therefore, tested whether this could be due to a loss of ROS production in these cells. Western blots confirmed that there was no NRF2 induction by LPS in TNF^−/−^ macrophages (Fig 4D). Similarly, upregulation of the NRF2 target gene HMOX1 did not occur in TNF^−/−^ cells, supporting the lack of NRF2 upregulation (Fig 4D). To confirm that this is due to a failure to upregulate ROS, we measured ROS production in TNF^−/−^ macrophages stimulated with LPS using mitoSOX red staining. Compared to wild‐type cells, TNF^−/−^ macrophages showed reduced ROS production (Fig 4E). We could also partially restore NRF2 levels and expression of HMOX1 in TNF^−/−^ cells treated with LPS, by exogenous addition of TNF; however, this was not significant (Fig 4F). Nevertheless, this coupled with the restoration of GM‐CSF‐induced IL‐1β secretion in TNF^−/−^ cells with exogenous TNF and subsequent inhibition with mDIVI support a role for TNF in driving ROS.

**Figure EV5:**
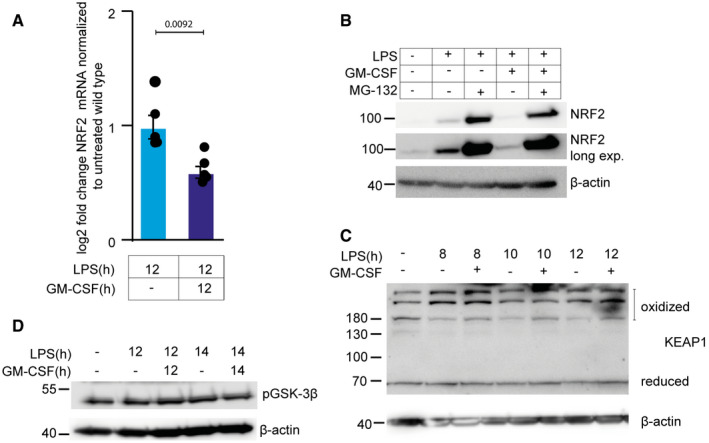
GM‐CSF‐regulated NRF2 stability independently of GSK3β HoxB8 macrophages were treated for 12 h with LPS or LPS + GM‐CSF and RNA was extracted and analyzed by qPCR for NRF2 mRNA.HoxB8 macrophages were treated for 12 h ± GM‐CSF. MG132 was added for 1 h prior to cells being lysed and analyzed for levels of NRF2 by western blot.HoxB8 macrophages were treated for the indicated times with LPS or LPS + GM‐CSF and proteins extracted and western blots made against KEAP1. High‐molecular‐weight bands represent oxidized KEAP1.HoxB8 macrophages were treated for 12 or 14 h with LPS or LPS + GM‐CSF. Proteins were analyzed by western blot for levels of phospho‐GSK3β. Data information: *n* ≥ 3 biological replicates (every dot represents one biological replicate). Error bars are SEM and *P*‐values were calculated using unpaired *t*‐tests. Westerns are representative images from three biological replicates. Source data are available online for this figure.

To understand how NRF2 upregulation is lost in GM‐CSF‐CSF‐treated cells, we first analyzed mRNA levels after GM‐CSF treatment. NRF2 mRNA was reduced by GM‐CSF treatment (Fig EV5A). NRF2 is normally continuously degraded through a proteasomal mechanism triggered by its binding to Kelch Like ECH‐Associated Protein 1 (KEAP1), which recruits Cullin 3 ubiquitin ligase to K48‐ubiquitylate NRF2 marking it for degradation (Baird & Yamamoto, 2020). To determine if differences in NRF2 mRNA are behind the reduced expression in GM‐CSF‐treated cells, the proteasome inhibitor MG132 was added to LPS or LPS + GM‐CSF‐treated cells. The addition of MG132 in GM‐CSF‐treated cells resulted in only slightly lower NRF2 levels, probably reflecting the reduced mRNA expression. This indicates that in GM‐CSF‐treated cells, there is still ample mRNA present to express NRF2, and that GM‐CSF rather regulates the stability of NRF2 (Fig EV5B). p62/SQSTM1, an autophagic cargo receptor, can regulate NRF2 levels through competition for and degradation of KEAP1, thus releasing NRF2 from degradation (Komatsu *et al*, 2010; Lau *et al*, 2010). No differences in the levels of KEAP1 could be seen in GM‐CSF‐treated cells, arguing against this being the mechanism (Fig EV5C). NRF2 turnover can also be regulated by βTrCP‐Cullin‐1‐mediated ubiquitylation in response to GSK3β signaling (Rada *et al*, 2011). Again, however, no differences in phosphorylation of GSK3 β could be detected upon GM‐CSF treatment arguing against this being the mechanism (Fig EV5D).

### GM‐CSF does not promote NLRP3 activation

**Figure 5:**
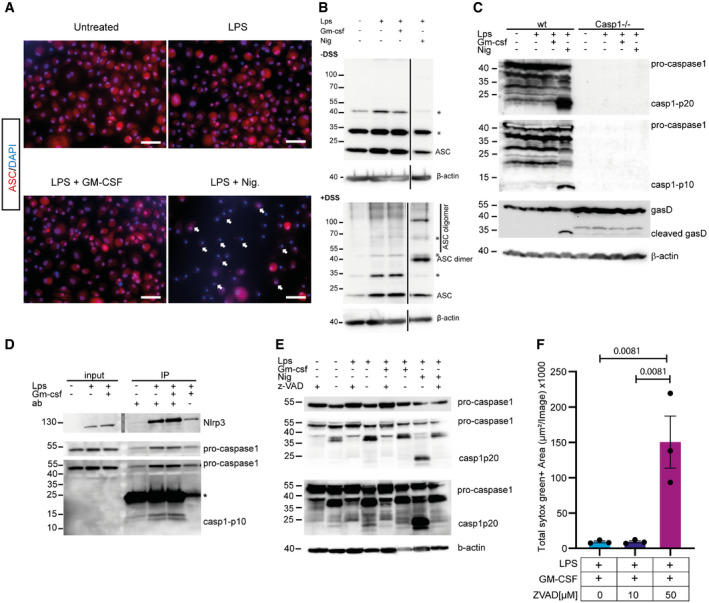
GM‐CSF does not activate NLRP3 inflammasomes HoxB8 macrophages were seeded in an 8‐well Ibidi chamber and treated as indicated for 12 h. Cells were fixed and stained for ASC (Red) and with DAPI (blue). For nigericin treatments, nigericin was added for only 30 min. Example cells containing ASC Specks are shown with white arrows. Scale bars represent 50 µm.HoxB8 cells were treated as indicated with LPS or LPS + GM‐CSF. The cross‐linker DSS was added to the cells as described in the Materials and Methods. Samples were analyzed by western blot for ASC oligomerization. Black vertical lines indicate that non‐relevant lanes were removed during figure preparation, but samples were run on the same gel.Wild‐type and caspase‐1^−/−^ HoxB8 macrophages were treated as indicated for 16 h. Cell lysates were made and analyzed by western blot for levels of caspase‐1 and gasdermin D cleavage.HoxB8 macrophages were treated as indicated with LPS or LPS + GM‐CSF for 12 h. Cells were lysed and NLRP3 was immune precipitated using a magnetic bead‐coupled antibody. As a negative control, beads without NLRP3 antibody were used. Beads were boiled and analyzed for the recruitment of NLRP3 and caspase‐1. * indicates light chain from the antibody. Molecular weight markers shown in the middle of the NLRP3 blot were embedded in the image using Chemostar imaging software (INTAS) at the time of image acquisition.HoxB8 macrophages were incubated with combinations of LPS, GM‐CSF, and nigericin ± Z‐VAD.FMK as indicated. Proteins were extracted and analyzed by western blot for caspase‐1 processing.HoxB8 macrophages were seeded in a 96‐well microplate and co‐treated with either 10 µM or 50 µM z‐VAD with LPS + GM‐CSF for 16 h. Then, 100 nM of SYTOX Green was added to the cells. The number of SYTOX green^+^ cells per image was quantified. Data information: Microscopy pictures and westerns are representative images from three biological replicates. Significance was calculated using one‐way ANOVA with multiple comparisons. Every dot represents one biological replicate. *P*‐values are shown. Source data are available online for this figure.

Our results show that GM‐CSF downregulates the antioxidant response in macrophages, but how this leads to increased IL‐1β secretion was not clear. Our results are consistent with increased NLRP3 activation driving more IL‐1β secretion. To test if NLRP3 inflammasomes are more active in GM‐CSF‐treated cells, several approaches were used. Firstly, ASC‐speck formation was analyzed by immunofluorescence. Although clear speck formation was detected upon LPS + Nigericin treatment, no specks could be detected in LPS + GM‐CSF treated cells (Fig 5A). The ASC‐complex formation can further be identified by chemical cross‐linking. However, no clear cross‐linking products of ASC could be seen upon GM‐CSF treatment but were readily detected upon LPS + Nigericin treatment (Fig 5B). When levels of cleaved caspase‐1 were examined by western blot, again very low levels of cleavage products p10 or p20 in LPS or LPS + GM‐CSF‐treated cells were found, although many other caspase‐1 bands could be detected with long exposure and substantial amounts of the active p10 and p20 fragments could be seen with Nigericin treatment (Fig 5C). Increased interaction of caspase‐1 with NLRP3 in response to LPS was found by immunoprecipitation of NLRP3, and the p10‐fragment was co‐precipitated and detectable in this assay (Fig 5D). However, no difference with the addition of GM‐CSF was seen. Interestingly, intermediate cleavage products of caspase‐1 were detected in both untreated and LPS or LPS + GM‐CSF‐treated cells including a significant 35 KDa fragment which probably reflects the CARD‐large subunit fragment. Addition of Z‐VAD.FMK blocked the formation of these products in all cases, showing that there are indeed caspase‐dependent cleavage products constitutively being produced (Fig 5E). To avoid induction of necroptosis through ZVAD inhibition of caspase‐8, we titrated ZVAD down to 10 µM and confirmed that this does not induce cell death (Fig 5F). These results indicate that, surprisingly, at least a small amount of active caspase‐1 is always present in macrophages. Together, these data strongly suggest that there is no significant increase in NLRP3 activity in response to GM‐CSF, despite increased NLRP3‐dependent IL‐1β secretion. They also highlight that there is a basal activation of caspase‐1 that is sufficient for the processing of IL‐1β without full assembly of ASC specks.

### IL‐1β is not secreted through autophagic or lysosomal secretory mechanisms but accumulates in membrane ruffles

**Figure 6:**
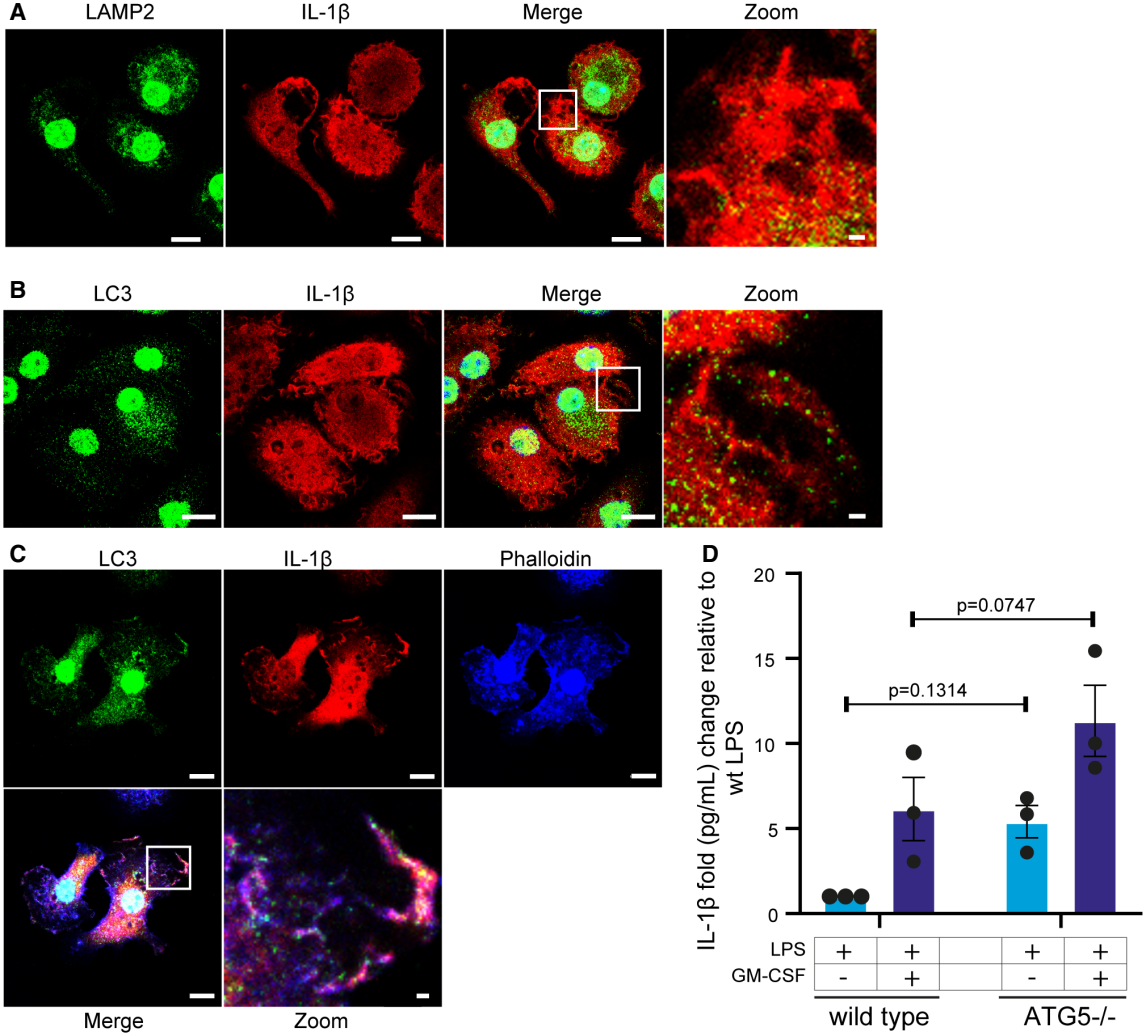
IL‐1β accumulates in membrane ruffles HoxB8 macrophages were seeded in an 8‐well Ibidi chamber and treated as indicated for 14 h with LPS and GM‐CSF. Cells were fixed and stained for LAMP2 (green) and IL‐1β (red). Images were taken at the Zeiss LSM880 confocal microscope.After treatment as in (A), cells were stained for LC3 (green) and IL‐1β (red).Cells treated and stained as in (B) and with phalloidin (blue).Wild‐type and ATG^−/−^ HoxB8 macrophages were treated as indicated for 16 h. Supernatants were analyzed for IL‐1β secretion by ELISA and shown is the fold change relative to the wild‐type LPS sample. Data information: *n* ≥ 3 biological replicates (every dot represents one biological replicate). For all panels, error bars are SEM. Significance was calculated using two‐way ANOVA to compare across genotypes *P*‐values are shown. Microscopy pictures are representative images from three biological replicates. Scale bars represent 10 µm except in zoomed panels where scale bars represent 1 µm.

To further understand how GM‐CSF‐stimulated IL‐1β is secreted, we used confocal microscopy to determine if IL‐1β localized with secretory compartments previously reported to secrete IL‐1β. We firstly analyzed co‐localization with LAMP2, a marker of lysosomes. IL‐1β has been reported to be secreted through lysosome‐derived secretory vesicles (Andrei *et al*, 2004). IL‐1β showed elongated structures, with only occasional co‐localization with LAMP2, suggesting lysosomal secretion is unlikely (Fig 6A). IL‐1β was also shown to be secreted by secretory autophagosomes (Kimura *et al*, 2017; Karmakar *et al*, 2020). We performed confocal microscopy against IL‐1β and LC3, a marker of autophagosomes. Strikingly, there is a clear co‐localization of LC3 puncta within the larger IL‐1β^+^ structures (Fig 6B). The larger wavy structures formed by IL‐1β are reminiscent of membrane ruffles which were previously shown to accumulate mature IL‐1β and were proposed to be the site of IL‐1β secretion (Monteleone *et al*, 2018). Membrane ruffles are formed by actin filaments, so we co‐stained cells treated with LPS+GM‐CSF with phalloidin and looked for LC3 and IL‐1β. Phalloidin showed strong co‐localization with IL‐1β, as did LC3, possibly suggesting autophagosomal secretion to the plasma membrane (Fig 6C). To determine if IL‐1β follows an autophagosomal secretory route, we generated ATG5^−/−^ HoxB8 cells and treated them with LPS or LPS + GM‐CSF. Contrary to IL‐1β requiring autophagosomes for secretion, loss of ATG5 actually increased IL‐1β secretion, both with LPS and LPS + GM‐CSF, although the differences were not significant (Fig 6D). This clearly shows that despite apparent co‐localization, autophagosomes are not required for IL‐1β secretion.

### GM‐CSF promotes IL‐1β ubiquitylation

**Figure 7:**
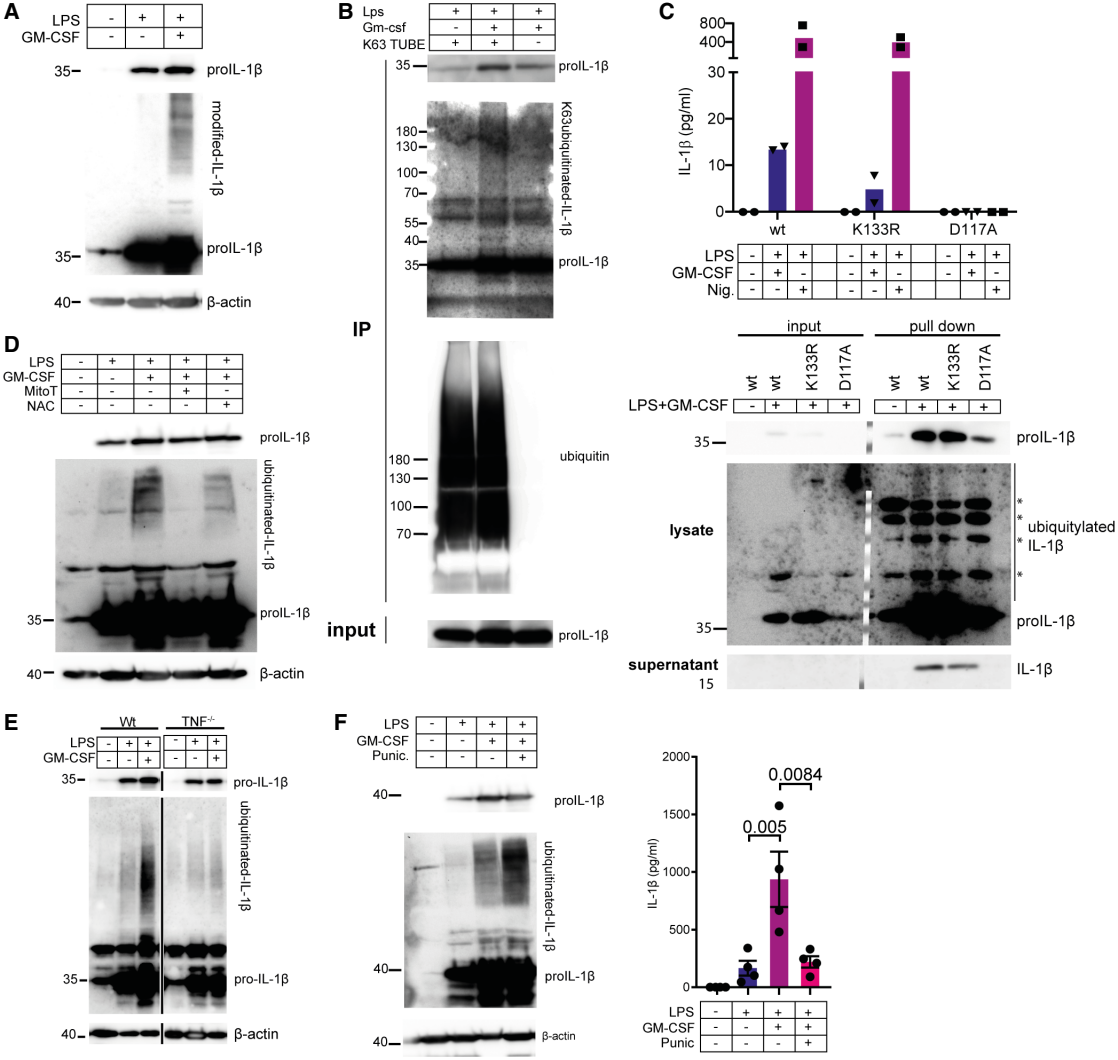
GM‐CSF‐induced ROS promotes IL‐1β ubiquitylation which drives IL‐1β secretion HoxB8 macrophages were treated for 16 h with LPS ± GM‐CSF as indicated. Proteins were extracted and analyzed for levels of IL‐1β by western blot.HoxB8 macrophages were treated with LPS or LPS + GM‐CSF as indicated for 16 h. Cells were lysed in buffer‐containing FLAG‐tagged K63 TUBE and K63 ubiquitylated proteins were precipitated using anti FLAG pull down. Pulled down proteins were analyzed for IL‐1β and ubiquitin by western blot.IL‐1β^−/−^ hoxB8 macrophages were infected with lentivirus expressing IL‐1β‐FLAG‐6xHis or the indicated mutants from the IL‐1β promoter. Cells were treated as indicated with LPS or LPS + GM‐CSF or LPS + Nigericin and levels of secreted IL‐1β were analyzed by ELISA (top panel). GM‐CSF treated cells were also lysed in urea buffer and nickel NTA affinity pull down was performed. Nickel NTA pull down was also performed on supernatants (bottom panel). Pulled down proteins form the His‐tag pull down were analyzed for the presence of IL‐1β by western blot. Molecular weight markers shown in the middle of the blot were embedded in the image using Chemostar imaging software (INTAS) at the time of image acquisition.HoxB8 macrophages were treated as in (A) with the addition of 50 µM mitoTEMPO or 10 mM NAC as indicated. Proteins were extracted and analyzed for IL‐1β by western blot.Wild‐type and TNF^−/−^ HoxB8 macrophages were treated as indicated with LPS or LPS + GM‐CSF. Proteins were extracted and analyzed for IL‐1β by western blot. Black vertical lines indicate that non‐relevant lanes were removed during figure preparation but samples were run on the same gel.HoxB8 macrophages were treated as in (A) but with the addition of 25 µM punicalagin throughout the 16 h time course. Proteins were extracted and analyzed by western blot (left panel). Supernatants were also analyzed by ELISA for secreted IL‐1β (right panel). Data information: *n* ≥ 3 biological replicates (every dot represents one biological replicate). For all panels, error bars are SEM. Significance was calculated using one‐way ANOVA with multiple comparisons, or two‐way ANOVA to compare across genotypes. *P*‐values are shown. Source data are available online for this figure.

When levels of IL‐1β were analyzed by western blot in cell lysates, clear modification of IL‐1β was seen, with high‐molecular‐weight signals detectable on western blots, which was strongly enhanced by the addition of GM‐CSF (Fig 7A). The smearing and increased molecular weight is suggestive of ubiquitylation. Ubiquitylation has been reported for IL‐1β previously and has been shown to promote its cleavage and maturation (Duong *et al*, 2015; Bulek *et al*, 2020). In order to test if IL‐1β is ubiquitylated in response to LPS + GM‐CSF, we used K63 TUBE pull downs and probed for IL‐1β. There was a clear enrichment of IL‐1β in the fraction of proteins bound by K63 TUBE, supporting the idea that IL‐1β is modified by K63‐linked ubiquitin chains (Fig 7B). IL‐1β is reported to be K63 ubiquitylated at lysine 133 (K133) and a target of A20 de‐ubiquitylation (Duong *et al*, 2015). To test if this residue is also the target in this system, we made IL‐1β^−/−^ HoxB8 cells and reconstituted them with lentivirus containing the IL‐1β promoter to drive LPS‐dependent expression of either wild‐type‐IL‐1β, K133R‐IL‐1β or a non‐cleavable mutant D117A‐IL‐1β where the caspase‐1 cleavage site is mutated, all containing a C‐terminal FLAG and 6xHIS tag. These cells were differentiated and treated with LPS or LPS + GM‐CSF and levels of IL‐1β secreted were measured by ELISA. Although expression levels were low, there was a strong reduction of secretion of K133R IL‐1β compared to wt, whereas mutation of D117 completely blocked secretion as expected (Fig 7C). IL‐1β was precipitated from supernatants using 6xHis tag purification, clearly showing a reduction of K133R‐IL‐1b secretion, although not a complete loss as was seen with the D117A mutant (Fig 7C). Cell lysates were also used for 6xHis tag purification in denaturing urea buffers to preserve ubiquitylation (Fig 7C). There was a significant, but not complete, reduction in the ubiquitylation of K133R‐IL‐1β.

**Figure 8:**
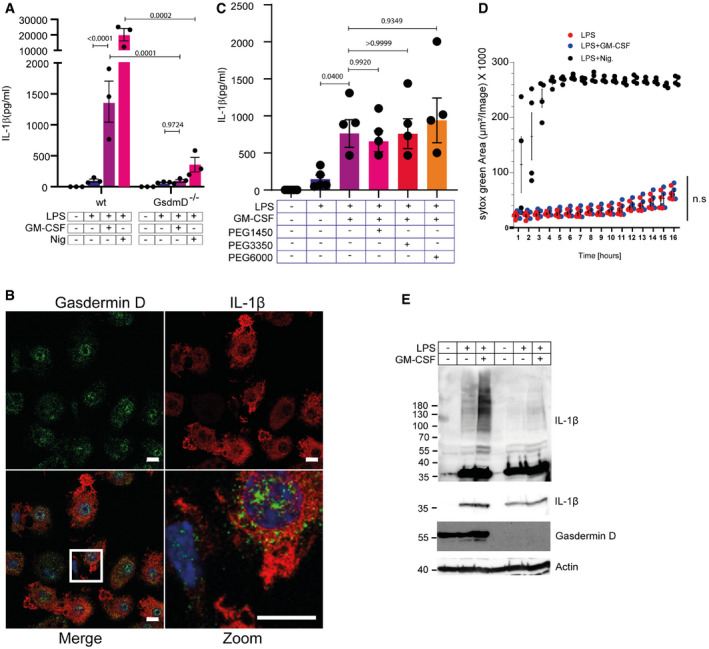
IL‐1β secretion occurs independently of pore formation Wild‐type and GsdmD^−/−^ HoxB8 macrophages were treated as indicated for 16 h. Supernatants were analyzed for IL‐1β secretion by ELISA.HoxB8 macrophages were seeded in an 8‐well Ibidi chamber and treated for 14 h with LPS and GM‐CSF. Cells were fixed and stained for gasdermin D (green), IL‐1β (red), and DAPI (blue). Images were taken at the Zeiss LSM880 Confocal microscope.HoxB8 macrophages were co‐stimulated with three different PEG 3 mM for 16 h as indicated. Supernatants were analyzed for IL‐1β secretion by ELISA.HoxB8 macrophages were seeded in a 96‐well microplate and treated as indicated. Before treatment, SYTOX green was added and cells were imaged in an IncuCyte incubator microscope every hour for 16 h. Shown is the SYTOX green signal normalized to the total cell area.Wild‐type and GsdmD^−/−^ HoxB8 macrophages were treated as indicated for 16 h. Proteins were extracted and analyzed for IL‐1β ubiquitination by western blot. Data information: *n* ≥ 3 biological replicates (every dot represents one biological replicate). For all panels, error bars are SEM. Significance was calculated using one‐way ANOVA with multiple comparisons, or two‐way ANOVA to compare across genotypes. *P*‐values are shown. Microscopy pictures and westerns are representative images from three biological replicates. Scale bars in microscopy images represent 10 µm. Source data are available online for this figure.

To determine the trigger for ubiquitylation of IL‐1β, we reasoned that ROS, which were required for the GM‐CSF‐triggered secretion of IL‐1β (Fig 2), may play a role in IL‐1β ubiquitylation. Indeed, ROS inhibition with mitoTEMPO blocked ubiquitylation of IL‐1β, as did NAC addition (Fig 7D). Further, the enhanced ubiquitylation induced by GM‐CSF was much reduced or absent in TNF^−/−^ cells (Fig 7E). The addition of punicalagin, a drug that blocks IL‐1β secretion by preventing the activation of membrane proteins such as pore‐forming gasdermin D (Martín‐Sánchez *et al*, 2016) blocked IL‐1β secretion and caused accumulation of the ubiquitylated form of IL‐1β in cells (Fig 6F), indicating a potential coupling of ubiquitylation and secretion. Gasdermin D can promote the secretion of IL‐1β from living macrophages, independent of its activation of pyroptosis (Evavold *et al*, 2018; Monteleone *et al*, 2018; Bulek *et al*, 2020). We asked whether gasdermin D was required for GM‐CSF‐induced IL‐1β secretion. When gasdermin D knockouts were tested, there was a complete block in IL‐1β secretion (Fig 8A). Confocal microscopy showed a vesicle‐like speckled distribution of gasdermin D which showed little co‐localization with membrane ruffle‐concentrated IL‐1β (Fig 8B). Additionally, we detected no gasdermin D cleavage after LPS + GM‐CSF treatment (Fig 5C). We further tested if pores formed by gasdermin D are required. To test this, we used incubation with polyethylene glycol (PEG) which blocks the osmotic release of cellular contents through gasdermin D pores (Fink & Cookson, 2006). Incubation with various sizes of PEG could not prevent IL‐1β release (Fig 8C). We also incubated the cells throughout the treatment with SYTOX green. If pores form during the treatment, even transiently, this should result in positive staining of nuclei with SYTOX green. Again, no significant staining was observed throughout the time course, and no differences between LPS and LPS + GM‐CSF were present (Fig 8D). The small increase seen corresponds to a low level of cell death present in the culture.

Full‐length gasdermin D was previously reported to promote IL‐1β ubiquitylation and subsequent processing and secretion (Bulek *et al*, 2020). Given we could detect neither cleaved gasdermin D, nor pore formation, but IL‐1β secretion was strongly dependent on gasdermin D, we tested to see if gasdermin D loss reduced IL‐1β ubiquitylation. Indeed, gasdermin D^−/−^ HoxB8 cells showed a clear reduction in ubiquitylated IL‐1β (Fig 8E). Gasdermin D is, therefore, required for IL‐1β secretion in response to GM‐CSF stimulation, possibly through regulation of IL‐1β ubiquitylation and transport, but not through pore formation. Together, these data support that indeed, K133 is the main target of IL‐1β ubiquitylation in response to ROS, but other residues are also ubiquitylated. Ubiquitylation of IL‐1β K133 as well as other lysine residues promotes its cleavage and/or secretion.

## Discussion

This study provides to our knowledge the first physiological link between GM‐CSF and the regulation of oxidative stress and inflammation, and this likely represents a pathway that is at play in inflammatory tissues. Macrophages alter their metabolism depending on treatment and also subset (Izquierdo *et al*, 2015; Mills *et al*, 2016). Depending on the type of macrophage or the stimulus, this can regulate how much ROS is generated. We have shown that GM‐CSF can potently trigger IL‐1β secretion in monocytic macrophages through a pathway requiring TNF‐driven mitochondrial ROS production and a blockage of the NRF2 antioxidant response. ROS are likely derived through RET and complex I, indicated by the ability of mDIVI‐1 (a complex I inhibitor) to block IL‐1β secretion. This corresponds to BMDMs that perform RET in response to LPS (Mills *et al*, 2016). In BMDMs, this is driven through the upregulation of IRG1 and subsequent inhibition of complex II by itaconate, the product of IRG1 activity. We could show that the GM‐CSF had no effect on IRG1 expression indicating that this pathway is not enhanced by its addition. Instead, GM‐CSF suppressed the upregulation of NRF2 in response to LPS, reducing the antioxidant response. Suppression of NRF2 expression occurred partly at the level of transcription, but more so through preventing its escape from proteolytic degradation. This was independent of KEAP1 degradation or changes in GSK3β activity. There are numerous other mechanisms for regulating the stability of NRF2, and it remains to be seen how GM‐CSF is able to regulate this process. NRF2 activity is associated with other inflammatory activities, and it could be that GM‐CSF regulation of NRF2 activity plays a significant part in its pro‐inflammatory function alongside IL‐1β secretion. We could, however, not detect GM‐CSF‐dependent regulation in many of the other cytokines tested other than IL‐6 and MCP‐1 suggesting it specifically promotes IL‐1β secretion.

Under GM‐CSF treatment, no increased NLRP3 activation was observed, rather there was a low‐level NLRP3 assembly in response to LPS and constitutive caspase‐1 activity, independent of LPS treatment. A recent model proposed for inflammasome activity whereby the majority of caspase‐1 substrate cleavage occurs with intermediately cleaved caspase‐1 that does not necessarily require speck formation (Boucher *et al*, 2018). The p35 fragment that is constantly present in HoxB8 macrophages may represent a constantly active caspase‐1 which is further recruited to NLRP3 in response to LPS. Exactly what drives this caspase‐1 activity is not clear however. A number of reports have demonstrated an activating role of ROS in NLRP3 inflammasome regulation, although there are conflicting studies suggesting this is not the case (Nakahira *et al*, 2011; Zhou *et al*, 2011; Yabal *et al*, 2018). We show instead that ROS drives ubiquitylation of IL‐1β, providing a signal to drive its cleavage and secretion. Together, these observations highlight that strong inducers of NLRP3 such as nigericin that lead to ASC speck formation are not necessary for the processing of IL‐1β, but rather the modification of IL‐1β itself can account for significant secretion by enhancing its processing by constitutively low levels of active caspase‐1.

We demonstrated that ROS promotes the ubiquitylation of IL‐1β at K133R and other residues. How ubiquitylation of IL‐1β regulates its processing and or secretion is unclear. We propose that at least for the slow prolonged IL‐1β secretion seen in this and other studies, ubiquitylation of IL‐1β at k133 is important to regulate this process. During the preparation of this manuscript, a publication was released demonstrating an important role in the ubiquitylation of K133. In this study, ubiquitylation was shown to reduce the stability of IL‐1β and the K133R mutation resulted in increased secretion of IL‐1β due to the higher levels of IL‐1β present in the cell (Vijayaraj *et al*, 2021). Why we see decreased secretion of IL‐1β may be due to differences in the experimental setup. We are using LPS + GM‐CSF as a stimulus, whereas Vijayaraj *et al*, have used stimuli such as ATP and nigericin. In our system, we could show that there is no difference in nigericin‐induced secretion of K133R‐mutated IL‐1β, supporting that a parallel pathway for IL‐1β secretion exists that requires ubiquitylation, whereas as classical stimuli such as ATP or nigericin do not. There is also a likely difference in the BMDM or 293T expression used versus the BMDMM used in this study.

Punicalagin treatment triggered the accumulation of ubiquitylated IL‐1β. Punicalagin stabilizes the plasma membrane, and it was reported to prevent mature IL‐1β from being secreted in response to ATP (Martín‐Sánchez *et al*, 2016). We were unable to determine if the ubiquitylated form of IL‐1β observed is the pro or mature form so cannot conclude for sure if ubiquitylation promotes cleavage or secretion; however, ubiquitylation of IL‐1β was previously proposed to promote IL‐1β cleavage suggesting it may be at that level (Duong *et al*, 2015).

The secretion of IL‐1β has had many potential mechanisms proposed. We show that IL‐1β, as previously described (Monteleone *et al*, 2018), accumulates in membrane ruffles after stimulation with LPS + GM‐CSF. LC3, a marker of autophagosomes, was also enriched within the membrane ruffles. We could show that loss of autophagy, using ATG5^−/−^ HoxB8 cells, rather than reducing IL‐1β secretion, actually increased it, suggesting that the co‐localization with LC3 may, in fact, reflect either a coincidental autophagosome accumulation within membrane ruffle regions or a potential degradative process for IL‐1β. LAMP2 showed little co‐localization with IL‐1β arguing against a secretory lysosomal pathway. IL‐1β, however, required gasdermin D for secretion. While pores formed by gasdermin D have been shown to be involved in IL‐1β secretion in the absence of cell death, our results show that IL‐1β release appears to be independent of pore activity. Additionally, gasdermin D could be detected within the membrane ruffles but was not enriched there like IL‐1β or LC3, nor was cleaved gasdermin D detectable. Full‐length gasdermin D has been shown in intestinal epithelial cells to promote IL‐1β ubiquitylation, cleavage by NLRP3 inflammasomes, and secretion (Bulek *et al*, 2020). We could also show reduced IL‐1β ubiquitylation in gasdermin D^−/−^ cells, indicating that a similar pathway may take place here to deliver IL‐1β to the membrane and or drive its secretion.

The mechanism how ROS regulate IL‐1β ubiquitylation remains unknown. However, one possibility could be through redox regulation of deubiquitinase (DUB) activity. DUBs function through a catalytic cysteine in their active site that can be reversibly targeted by ROS, inhibiting DUB activity (Cotto‐Rios *et al*, 2012; Kulathu *et al*, 2013; Lee *et al*, 2013). A20 is known to be susceptible to such regulation (Kulathu *et al*, 2013). Given its identification as a DUB acting on IL‐1β (Duong *et al*, 2015), it could well be that A20 activity is suppressed in cells treated with GM‐CSF; however, further work is needed to confirm this.

The fact that we could not trigger IL‐1β secretion in BMDM with GM‐CSF treatment highlights that BMDM are functionally different in their responses to LPS from monocytic macrophages. This may in part be due to the propensity of BMDM to produce IL‐10 in response to LPS (Lacey *et al*, 2012; Gurung *et al*, 2015; Budai *et al*, 2017; Wilbers *et al*, 2018; Ipseiz *et al*, 2020). We could largely block GM‐CSF‐induced IL‐1β secretion through co‐treatment with IL‐10. Humans with mutations in IL‐10 signaling manifest with severe inflammatory bowel diseases (Glocker *et al*, 2011). IL‐10 was also reported to prevent LPS‐induced free radical formation in macrophages and brain tissue (Dokka *et al*, 2001; Gao *et al*, 2020), and IL‐10 can also regulate IL‐1β secretion through regulation of macrophage metabolism and mitochondrial ROS production (Ip *et al*, 2017). Together, these results suggest that GM‐CSF‐induced IL‐1β secretion may follow a similar mechanism to macrophages deficient for IL‐10 or IL‐10 receptor. To date, no antioxidant therapies are used therapeutically for inflammatory diseases driven by IL‐1β. A number of diseases are associated with spontaneous inflammasome activation such as CAPS and FMF as well as IL‐1β‐driven pathologies such as SJIA. Little work has been done to see if ROS inhibition could potentially be used to treat these diseases, but this study may indicate that antioxidant therapies may help dampen the secretion of IL‐1β. GM‐CSF functions as a pro‐inflammatory cytokine and plays a driving role in diseases such as rheumatoid arthritis and probably many other inflammatory diseases. These auto‐inflammatory diseases may also benefit from anti‐GM‐CSF therapy if it turns out that it also plays a role in promoting ROS in such an inflammatory setting.

Together, this study shows that LPS is able to induce mitochondrial ROS in a TNF‐dependent pathway and that co‐signaling by GM‐CSF enhances the potency of the ROS by suppressing the NRF2 pathway. This results in the ubiquitylation of IL‐1β that promotes processing by constitutively low‐level caspase‐1 activity and secretion of IL‐1β through a gasdermin D‐dependent pathway. Understanding the extent to which GM‐CSF antioxidant regulation impacts its pro‐inflammatory as well as differentiation functions will be of great interest for understanding the effects of GM‐CSF on various pathological inflammatory diseases.

## Materials and Methods

### Cell culture, treatments

Hoxb8 macrophage progenitors were cultured in very low endotoxin (VLE) RPMI supplemented with 10% FCS (heat inactivated), 1% Pen/Strep, 1% glutamine, 1 μM β‐estradiol, and 1% culture supernatant from a GM‐CSF‐producing cell line (corresponding to ~ 2 ng/ml GM‐CSF). They were cultured in a 6‐well plate and split every 2 days, without exceeding the confluence of 1.5 × 10^6^ cells/ml. Cell culture was performed using standard culture conditions (37°C, 5% CO_2_) in a humidified incubator, and cells were replaced every 6 weeks by a freshly thawed stock of cells. For differentiation, the progenitors were washed from β‐estradiol 3× with PBS and resuspended in media without β‐estradiol. Cells were seeded at 5 × 10^5^ cells/plate in a 10‐cm non‐tissue culture‐treated dish. After 5 days, 100 µl fresh GM‐CSF/dish was added. Attached differentiated macrophages were harvested using Accutase on Day 7 and used for experiments. For experimental treatments, cells were seeded at 4 × 10^5^ cells/well in a 24‐well non‐tissue culture‐treated plate (Corning cat#351147) in VLE‐RPMI without GM‐CSF. Where indicated cells were treated as follows: using 60 ng/ml UP‐LPS (InvivoGen), 1% of supernatant containing GM‐CSF, 10 ng/ml E‐Coli rec.GM‐CSF, 2‐ng/ml HEK rec.GM‐CSF, 40 µM Z‐WEHD‐FMK (caspase‐1 inhibitor, R&D System), 0.5 µM CRT 0066101 (PLC inhibitor), 50 µM MitoTEMPO (Cayman Chemical), 25 µM punicalagin (Cayman Chemical), 100 ng/ml rIL‐10 (PeproTech), 50 mM KCl, 100 ng/ml rTNFα, Nigericin 5 µM, 3 mM PEG of different sizes (1,450 MW; 3,350 MW, and 6,00 MW(Sigma‐Aldrich)), 10 ng/ml *E. coli* recombinant M‐CSF (PeproTech), and 10 µM or 50 µM z‐VAD.FMK (Hycultec).

### Generation of HoxB8 macrophage progenitors

For the production of HoxB8‐immortalized macrophages, we isolated bone‐marrow cells from Balb/c mice, by flushing femurs and tibia with PBS, a part from Atg5^−/−^ macrophages that were isolated from fetal liver. Bone marrow and fetal liver cells were so collected into a 50‐ml conical tube and pelleted at 1,500 rpm for 5 min. Pellet cells were resuspended in 2 ml ACK red blood cell lysis buffer and incubated for 2 min at RT. Lysis was stopped by the addition of 18 ml of PBS. Cells were again pelleted and resuspended in “Stem cell media” consisting of RPMI with 15% FBS, 1% P/S, 1% glutamine, 10 ng/ml mIL‐3, 20 ng/ml mIL‐6, and 1% supernatant from SCF‐producing B16 melanoma cells. For 2–3 days, cells were cultured at 1 × 10^6^/ml or 5 × 10^5^/ml in this cytokine‐rich media. Then, the media were replaced with “Progenitor outgrowth medium” (VLE‐RPMI with 10% FBS, 1% P/S, 1% glutamine, 1‐µM estradiol, and 1% culture supernatant from a GM‐CSF‐producing cell line). 1 × 10^5^ cells/ 250 µl progenitor outgrowth medium was infected with 500 μl Hoxb8 retrovirus supernatant supplemented with 5 μg/ml polybrene in a 12‐well plate by spin infection at 1,800 *g* for 90 min at 30°C. One milliliter of progenitor outgrowth medium was added to the cells after spinning (Wang *et al*, 2006). The non‐adherent together with the lightly adherent cells were split and transferred into a new well every 3–4 days for 4 weeks until the cell populations were stably expanding.

For TNF^−/−^, ASC^−/−^, NLRP3^−/−^, CASP1^−/−^, and IL‐1β^−/−^ cell lines, the bone‐marrow cells were isolated from the correspondent knockout mice and generated as described above.

### Primary bone marrow‐derived monocytic macrophages and bone marrow‐derived macrophages

Primary Bone Marrow‐Derived Monocytic Macrophages (BMDMMs) were obtained from the tibia and femoral bones of C57/B6 wild‐type mice. Cells were cultured for 7 days with 20 ng/ml recombinant GM‐CSF in RPMI (10% FCS and 1% Pen/Strep) in Petri dishes. On Day 7 of differentiation, the attached macrophages were harvested using accutase. For experiments, cells were seeded at 4 × 10^5^ cells/well in a 24 non‐tissue culture‐treated plate and treated as described. For Bone Marrow‐Derived Macrophages (BMDM), cells were obtained from the tibia and femoral bones of C57/B6 wild‐type mice. These were incubated in RPMI (10% FCS and 1% Pen/Strep) containing 20 ng/ml recombinant M‐CSF (PeproTech cat. No. 300‐25) for 7 days. Attached macrophages were harvested with accutase and seeded at 4 × 10^5^ cells/well for indicated treatments.

### Generation of 6xHisTag_FLAG_IL1‐β reporters

For the cloning of IL‐1β, a 1 × 10 cm^2^ dish of HoxB8‐differentiated macrophages was stimulated for 4 h with LPS, and the RNA was isolated using the High‐Pure RNA Isolation Kit (Roche). cDNA was made using the Transcriptor First‐Strand cDNA Synthesis Kit (Roche).

PCR using Q5 High‐Fidelity DNA Polymerase was done using the following primers for IL‐1β (Forward: CACCATGGCAACTGTTCCTGAACT; Reverse: TCAGGAAGACACGGATTCCATG). The PCR product was run on a 1% agarose gel and purified using a Wizard gel purification kit (Promega). The IL‐1β ORF was inserted into the pENTR/D TOPO donor vector (Thermo) using directional TOPO cloning as described in the manual. Mutations in IL‐1β were generated using the Q5 mutagenesis kit (NEB) using the following primers, K133R (Forward:TGAACAACAAcgAAGCCTCGTGCTGTCG, Reverse: TCTCGGAGCCTGTAGTGC), D117A (Forward:CTGGTGTGTGcCGTTCCCATT, Reverse: CAGGTTATCATCATCATCCC). A C‐terminal 6xHIS‐FLAG tag was added using the HiFi assembly kit (NEB) using the following primers (Reverse: TCAGTGGTGGTGATGATGATGAAGCTTGAATTCTTTATCATCATCATCTTTATAATCGGAAGACACGGATTCCATGGTGAAG). For LPS‐inducible expression of IL‐1β, the pFIL‐1β‐GW‐SV40‐Puro vector was made as follows: 600‐bp upstream of the transcriptional start site of the mouse IL‐1β gene was amplified using Q5 polymerase using the following primers (Forward: gcaTTAATTAAtggcattatcagactgcatagg, Reverse: gcaGCTAGCgtccaacttgttttccctcc). The PCR product was cut using PacI and NheI restriction enzymes (NEB). The destination vector pFEF1α‐GW‐SV40‐Puro was digested using PacI and NheI restriction enzymes and both constructs were purified from 1% agarose gel. The PCR products were ligated using T4 DNA ligase (NEB) and the PCR product was transformed into CCDB‐Stable E. Coli (Thermo). IL‐1β ORf or mutants thereof were cloned into this vector using gateway cloning with LR recombinase (Thermo).

Cell lines expressing these constructs were made by generating lentiviruses expressing IL‐1β or its corresponding mutants as previously described. After infection, cells were selected using 3.5 µg/ml puromycin until uninfected control cells were 100% dead.

### Measurement of cytokine secretion

HoxB8‐differentiated macrophages were plated 4 × 10^5^/well in a 24‐well plate the day of stimulation in VLE‐RPMI without GM‐CSF. Cells were left unstimulated and co‐stimulated for 16 h with ultrapure LPS (InvivoGen) and 1% GM‐CSF‐conditioned media, Necrostatin‐1 10 µM (Sigma), Z‐WEHD‐FMK 40 µM (R&D System), MitoTEMPO 50 µM (Cayman Chemical), punicalagin 25 µM (Cayman Chemical), G3SK 3 µM, rTNFα 100 ng/ml, zVAD 20 µM (Bachem), and Nigericin 5 µM for 1 h. mIL‐1β and mTNF were detected from cells supernatant by ELISA.

### Quantitative reverse transcription PCR (qRT–PCR)

RNA isolation was performed using the Direct‐zol RNA Miniprep Kit (Zymo Research) and RNA quality was assessed using NanoDrop Spectrometry. Then, the genomic DNA was obtained using the *Transcriptor First‐Strand cDNA Synthesis Kit* (Roche).

Gene expression was determined by using the SYBER Green detection system and the following primers for NRF2 (forward: AGATGACCATGAGTCGCTTGC, reverse: GCCAAACTTGCTCCATGTCC), HMOX‐1 (forward: CCAGAGAAGGCTTTAAGCTGGT, reverse: TGGGGCATAGACTGGGTTCT), NQO1 (forward: GTGCTCGTAGCAGGATTTGC, reverse: AACGCAGGATGCCACTCTGA), Irg1 (forward: GCGAACGCTGCCACTCA, Reverse: ATCCCAGGCTTGGAAGGTC), IL‐1β (forward: TGT CTT GGC CGA GGA CTA AGG; reverse:TGG GCT GGA CTG TTT CTA ATG). Fold change was calculated using the ΔΔCt method with β‐Actin acting as an internal standard.

### Western blot analysis

Cell lysates were made by lysing 4 × 10^5^ cells in 100 µl of 6 M urea lysis buffer including a proteasome inhibitor (Roche), DUBs inhibitor NEM (Thermo Fisher), and PhosSTOP (Roche). Proteins were boiled in 1x LB containing DTT at 95°C for 6 min, and 50 µl of the lysate was used for electrophoresis on 4–20% polyacrylamide gradient gel (Invitrogen). Next, the proteins were transferred to a nitrocellulose membrane (wet transfer) and blocked using 5% non‐fatty milk in TBST for 1 h with gentle agitation. The membrane was incubated overnight with antibodies against IL‐1β (1:1,000, R&D System), Casp1p10 (1:1,000, Cell Signalling), Casp1p20 (1:1,000, AdipoGen), ASC (Merck), gasdermin D (1:1,000, Cell Signalling), Nrf2 (1:1,000, Cell Signalling), HMOX1 (1:1,000, Novus), β‐actin (1:50,000, Sigma).

Western blots were quantified using ImageJ software. All densitometry analyses were performed on images saved in TIFF format. For every experiment, the signal of the protein of interest was normalized to the loading control (β‐actin, tubulin, or GAPDH). We showed either the signal intensity after normalization or the fold change relative to LPS and LPS+GM‐CSF treatment (as indicated).

### ROS production

After 8 h treatment, cells were stained directly in media in a 24‐well plate with 5 µM mitoSOX red and incubated at 37°C, 5%CO_2_ for 20 min. Cells were washed 1x PBS, harvested using accutase, and re‐suspended in FACS buffer (PBS + 1% FCS). The fluorescent signal of mitoSOX positive cells was detected in the FL‐2 channel using FACSCalibur.

### Ni‐NTA affinity purification

For the selective purification of 6xHis_Tag_FLAG_IL‐1β constructs from total cell lysate, we performed Ni‐NTA affinity purification as described in the manufacturer's protocol (Qiagen, #30210). For each reaction, 1 mg of cell lysate was incubated with 25 µl Ni‐NTA agarose at 4°C in lysis buffer (0.1 M NaH_2_PO_4_, 6 M Urea, 130 mM NaCl, 5 mM imidazole, 10 mM Tris, 1% CHAPS, 1x Protease inh., 1 mM PMSF, 30 mM NEM, pH = 8.0). After 4 h of incubation time, the beads were washed using 20 ml of wash buffer (Lysis buffer with 20 mM imidazole, pH = 6.8) and incubated for 20 min at RT with the elution buffer (0.1 M NaH_2_PO_4_, 6 M Urea, 130 mM NaCl, pH = 4.3). Next, the beads were spun down, and the purified proteins were boiled in 1x SLB for 5 min at 95°C. Pulled down proteins together with the lysate kept from the input were run on 4–20% polyacrylamide gradient gel (Invitrogen) and transferred to a nitrocellulose membrane. Antibody against IL‐1β was used to detect precipitation of the ubiquitinated protein.

### K63‐TUBE IP

The pull down of K63‐ubiquitinated protein was performed as described in the manufacturer's protocol (LifeSensors, #UM604). The pull‐down 2 × 10 cm plate with around 1–1.5 × 10^6^ differentiated macrophages was lysed using 200 µl of lysis buffer (100 mM Tris–HCl, 150 mM NaCl, 5 mM EDTA, 1% NP40, 0.5% TritonX‐100, protease inhibitor, phosSTOP, 25 mM NEM, 5 mM PM, 75 µM PR‐619, and 220 nM FLAG TUBE). Around 1.5 mg of proteins was used for the reaction. The lysate was diluted 1:5 into a reaction buffer, consisting of lysis buffer without detergent + 220 nM of FLAG TUBE (in order to dilute the detergent out). For the negative control, no TUBE reagent was added. Then, anti‐FLAG M2 affinity beads and lysate were incubated at 4°C shaking for 2 h. After the incubation time, the beads were washed with 15 ml of washing buffer (100 mM Tris–HCl, 150 mM NaCl, 5 mM EDTA, and 0.05% NP40) and boiled in 3x SLB at 95°C for 5 min. At last, the beads were pelleted down and the boiled proteins moved into a new Eppendorf tube. Proteins were run on 4–20% polyacrylamide gradient gel (Invitrogen) and transferred to a nitrocellulose membrane. Antibody against IL‐1β was used to detect precipitation of the ubiquitinated protein.

### ASC immunofluorescence

HoxB8‐differentiated macrophages were treated for 12 h with LPS with or without GM‐CSF and with LPS+Nigericin for the last 1 h in an 8‐well Ibidi chamber. Next, cells were fixed using 4%PFA and permeabilized with 0.2% Triton‐X. After 30 min Fc block, cells were stained with anti‐ASC antibody (AL177, AdipoGen) for 1 h, followed by an anti‐rabbit secondary ab. Last, 0.2 µg/ml DAPI was added to the cells for 10 min. Stained cells were imaged for ASC Speck structure using a confocal LSM 880 microscope equipped with a 63x oil objective keeping the laser settings of images constant for comparison.

### Confocal microscopy

HoxB8‐differentiated macrophages were seeded 1 × 10^5^/well in an 8‐well microscope slide (Ibidi) and treated with LPS + GM‐CSF for 14 h. After stimulation, wells were washed 2x with PBS, fixed for 10 min in 5% PFA in PBS, and permeabilized using 0.2% Triton‐X100 in PBS for 10 min. Next, cells were incubated for 1 h at room temperature (RT) with the following primary antibodies: anti‐IL‐1β 1:100 dilution (#AF‐401‐NA, R&D System), anti‐LC3 1:200 dilution (#nb100‐2220, Novus), anti‐LAMP‐2 1:100 dilution (#ab13524, Abcam), and anti‐gasdermin D 1:100 dilution (#93709, Cell signaling). Cells were incubated for 1 h at RT with the respective secondary antibodies using 1:500 dilution: anti‐goat‐DyLight 649 (#705‐495‐147, Dianova), anti‐rabbit‐AlexaF488 (#711‐545‐152, Dianova), and anti‐rat‐Cy3 (#712‐165‐150, Dianova). Also, Alexa Fluor 546 phalloidin (#A22283, Thermo Fisher) was added at 1:40 dilution and incubated for 1 h with the secondary antibodies. Last, cell nuclei were stained for 10 min with DAPI. After 2× washing with PBS, cells were visualized in Ibidi mounting media at the Zeiss LSM880 using 63× oil objective.

### DSS cross‐linking

Cells were treated as described above, washed with PBS, and incubated in PBS with 2.5 mM DSS for 30 min at RT. The reaction was quenched using 10 mM Tris for 15 min, and the cells were lysed in 1x LB and boiled at 95°C. Proteins were used for WB and probed with an antibody against ASC.

### Cell death analysis

Cells were treated as described above and co‐stained with 100 nM SYTOX Green. Fluorescence images were taken using an IncuCyte live‐cell imager with 20× objective and 300‐ms exposure time. For quantification, the cell death was measured as the area of SYTOX green signal compared to the area covered by cells throughout the plate.

### Surface markers FACS staining

For the surface marker staining, differentiated macrophages were harvested using Accutase and 2 × 10^4^ cells were stained using anti‐CD11c‐PECy7 (eBioscience), anti‐CD11b‐APC (BD), anti‐MHCII‐FITC (BD), and anti‐CD115‐PE (eBioscience) antibodies diluted in FACS buffer (PBS + 1% FCS) supplemented with Fc‐blocking ab. After 1 h of incubation, the cells were washed 2x PBS and resuspended in FACS media. The analysis was performed using FACS Fortessa and data were analyzed using FlowJo software.

### Quantification of multicytokine secretion

The supernatant of treated macrophages was analyzed for cytokine secretion using a bead‐based assay (LEGENDplex™ Mouse Anti‐Virus Response Panel (13‐plex), BioLegend) according to the manufacturer’s protocol. Samples were recorded using FACS Fortessa, and the data were analyzed with the BioLegend online software.

## Author contributions

**Sara Di Carlo:** Conceptualization; Resources; Formal analysis; Investigation; Visualization; Methodology; Writing—original draft; Writing—review & editing. **Georg Häcker:** Conceptualization; Supervision; Funding acquisition; Writing—original draft. **Ian E Gentle:** Conceptualization; Resources; Formal analysis; Supervision; Visualization; Methodology; Writing—original draft; Project administration; Writing—review & editing.

In addition to the CRediT author contributions listed above, the contributions in detail are:

Conceptualization: SDC, IEG, GH; Methodology: SDC, IEG; Investigation: SDC, IEG; Writing—original draft: SDC, IEG, GH; Writing—review & editing: SDC, IEG, GH; Funding acquisition: GH; Resources: SDC, IEG, GH; Supervision: IEG, GH.

## Disclosure and competing interests statement

The authors declare that they have no conflict of interest.

## Supporting information



Expanded View Figures PDF

Source Data for Expanded View

Source Data for Figure 1

Source Data for Figure 4

Source Data for Figure 5

Source Data for Figure 7

Source Data for Figure 8

## Data Availability

This study includes no data deposited in external repositories.
