# Supplementary material for: GM‐CSF suppresses antioxidant signaling and drives IL‐1β secretion through NRF2 downregulation
Source: EMBO Rep. 2022 Jun 13;23(8):e54226. doi: 10.15252/embr.202154226 (PMC9346485; doi:10.15252/embr.202154226)
Supplement: Supplementary file 1 — Expanded View Figures PDF [file EMBR-23-e54226-s002.pdf]

Expanded View Figures

Figure EV1. HoxB8 macrophages are similar to bone marrow-derived monocytic macrophages.

Shown is the surface staining of markers for monocytic macrophages. All cells were stained as described in the Materials and Methods and staining was detected using flow cytometry.

Data information: Staining is representative of $n \geq 3$ biological replicates.

© 2022 The Authors

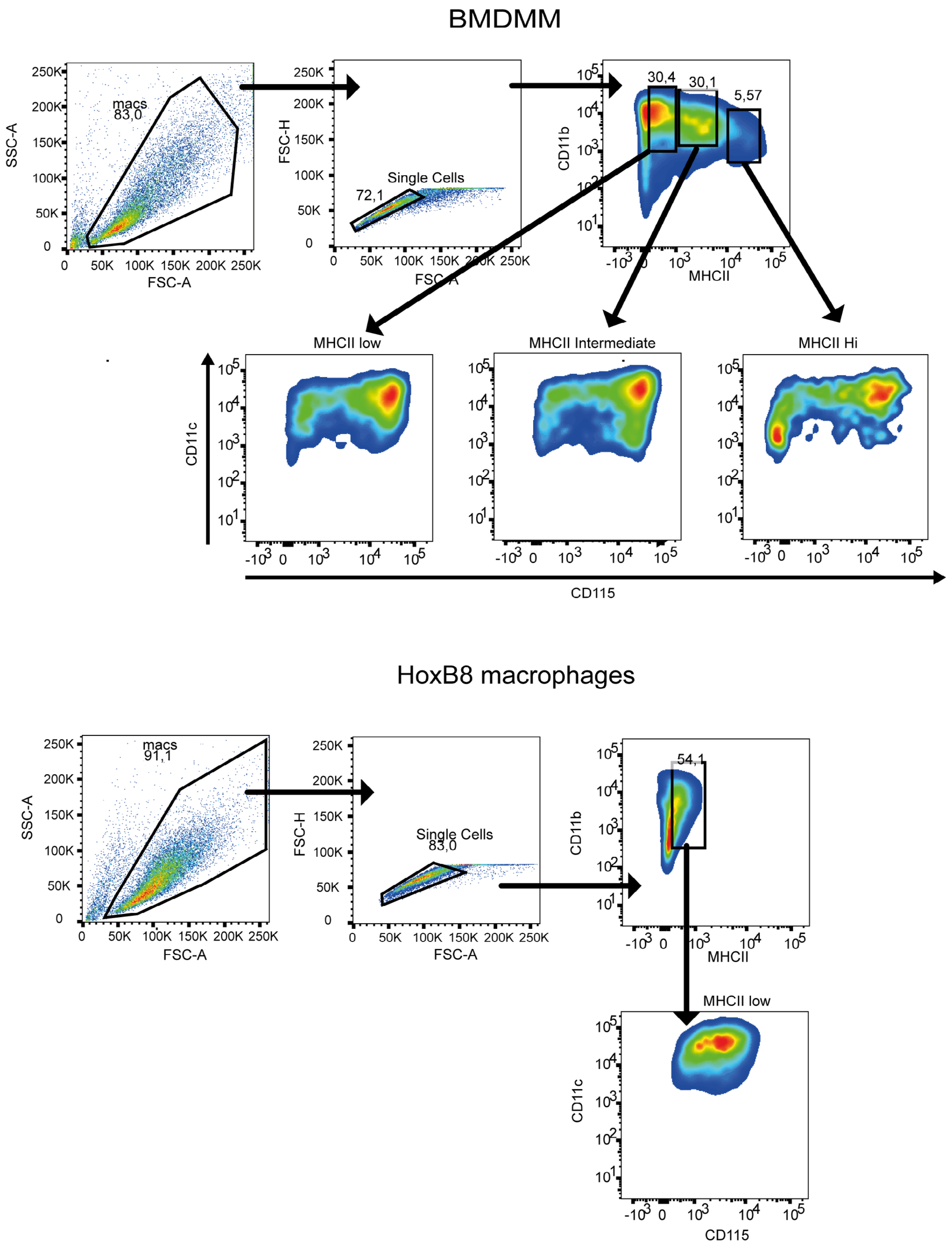
BMDMM
macs
83,0
SSC-A
FSC-A
FSC-H
Single Cells
72,1
CD11b
MHCII
30,4
30,1
5,57
MHCII low
MHCII Intermediate
MHCII Hi
CD11c
CD115
HoxB8 macrophages
macs
91,1
Single Cells
83,0
54,1
MHCII low

Figure EV1.

Figure EV2. Quantification of IL-1β mRNA and protein levels.

A HoxB8 macrophages were treated for 12 h as indicated. The RNA was extracted and analyzed by qPCR for IL-1β.
B Densitometric quantification of pro-IL-1β protein level relative to LPS-treated samples.
C HoxB8 macrophages were co-treated with 10 ng/ml recombinant M-CSF for 16 h as indicated. Media were analyzed for IL-1β secretion by ELISA.
D HoxB8 macrophages were treated as indicated using 60 ng/ml UP-LPS and or 1% of GM-CSF supernatant. IL-1β secretion was analyzed in the supernatant by ELISA.
E Densitometric quantification of pro-IL-1β protein level in the indicated HoxB8 genotypes treated for 16 h with LPS in the presence or not of GM-CSF. Shown are representative western blots of pro-IL-1β (right panel). Black vertical lines indicate non-relevant lanes that were removed during figure preparation. All samples were run on the same gel.
F HoxB8 macrophages were treated with the indicated concentrations of either media from GM-CSF producing cells, *E. coli*-expressed recombinant GM-CSF, or HEK293-expressed recombinant GM-CSF plus LPS. Supernatants were analyzed for IL-1 β by ELISA.
G Densitometric quantification of pro-IL-1β protein level relative to LPS-treated samples. HoxB8 macrophages were treated as indicated for 16 h. Data correspond to Fig. 2A. Below are shown representative western blots.
H Densitometric quantification of pro-IL-1β protein level relative to LPS-treated samples. HoxB8 macrophages were treated as indicated for 16 h. Data correspond to Fig. 2C. Below are shown representative western blots.
I Densitometric quantification of pro-IL-1β protein level relative to LPS-treated samples. HoxB8 macrophages were treated with indicated doses of supernatant containing GM-CSF for 16 h.

Data information: $n \geq 3$ biological replicates (every dot represents one biological replicate). For all panels, error bars are SEM. Significance was calculated using unpaired *t*-test. *P*-values are shown.
Source data are available online for this figure.

 © 2022 The Authors

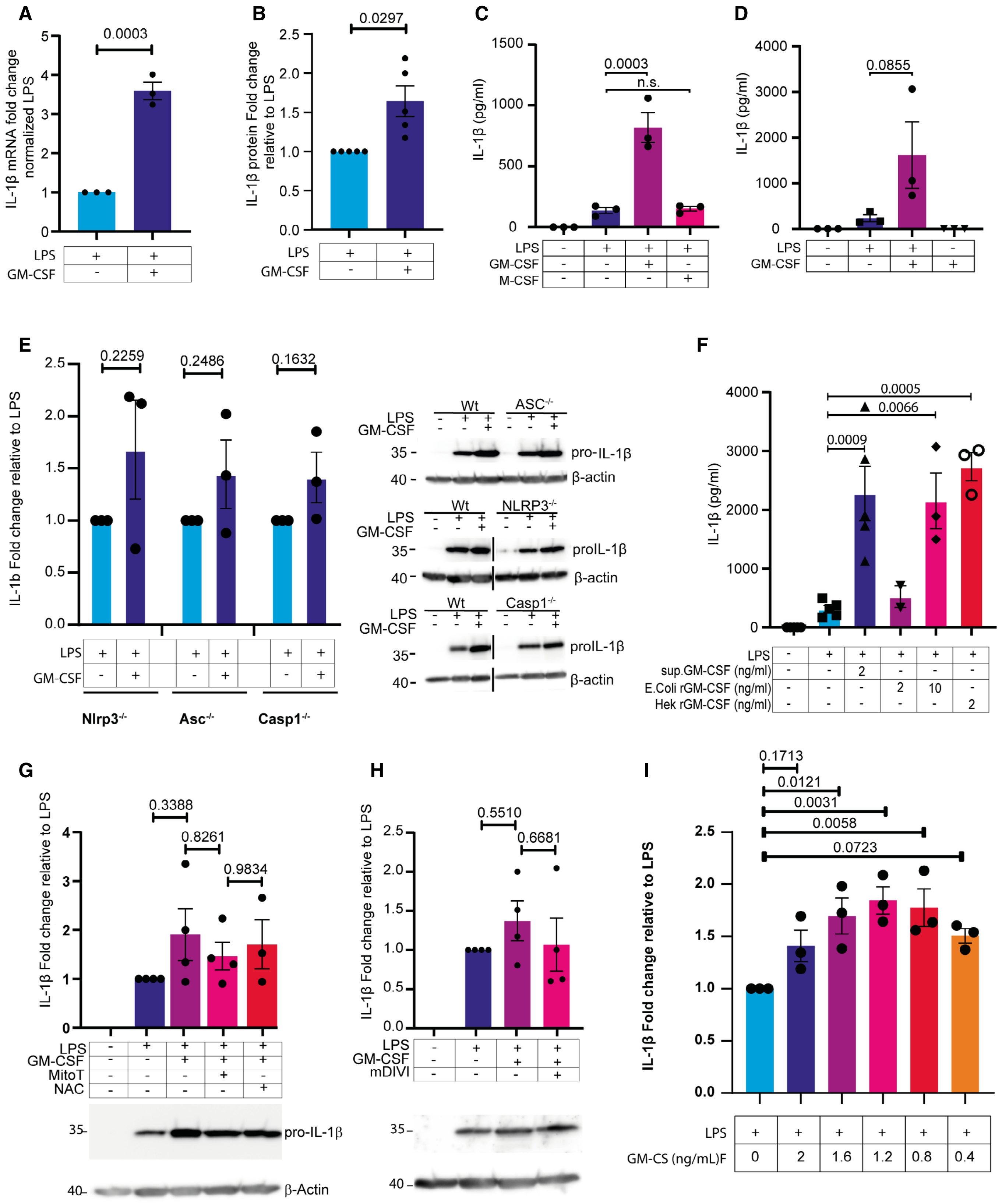
A
B
C
D
E
F
G
H
I
IL-1β mRNA fold change normalized LPS
IL-1β protein Fold change relative to LPS
IL-1β (pg/ml)
IL-1b Fold change relative to LPS
IL-1β Fold change relative to LPS
LPS
GM-CSF
M-CSF
0.0003
0.0297
n.s.
0.0855
0.2259
0.2486
0.1632
Nlrp3-/-
Asc-/-
Casp1-/-
Wt
ASC-/-
NLRP3-/-
Casp1-/-
pro-IL-1β
proIL-1β
β-actin
0.0005
0.0066
0.0009
sup.GM-CSF (ng/ml)
E.Coli rGM-CSF (ng/ml)
Hek rGM-CSF (ng/ml)
0.3388
0.8261
0.9834
MitoT
NAC
β-Actin
0.5510
0.6681
mDIVI
0.1713
0.0121
0.0031
0.0058
0.0723
GM-CS (ng/mL)F

Figure EV2.

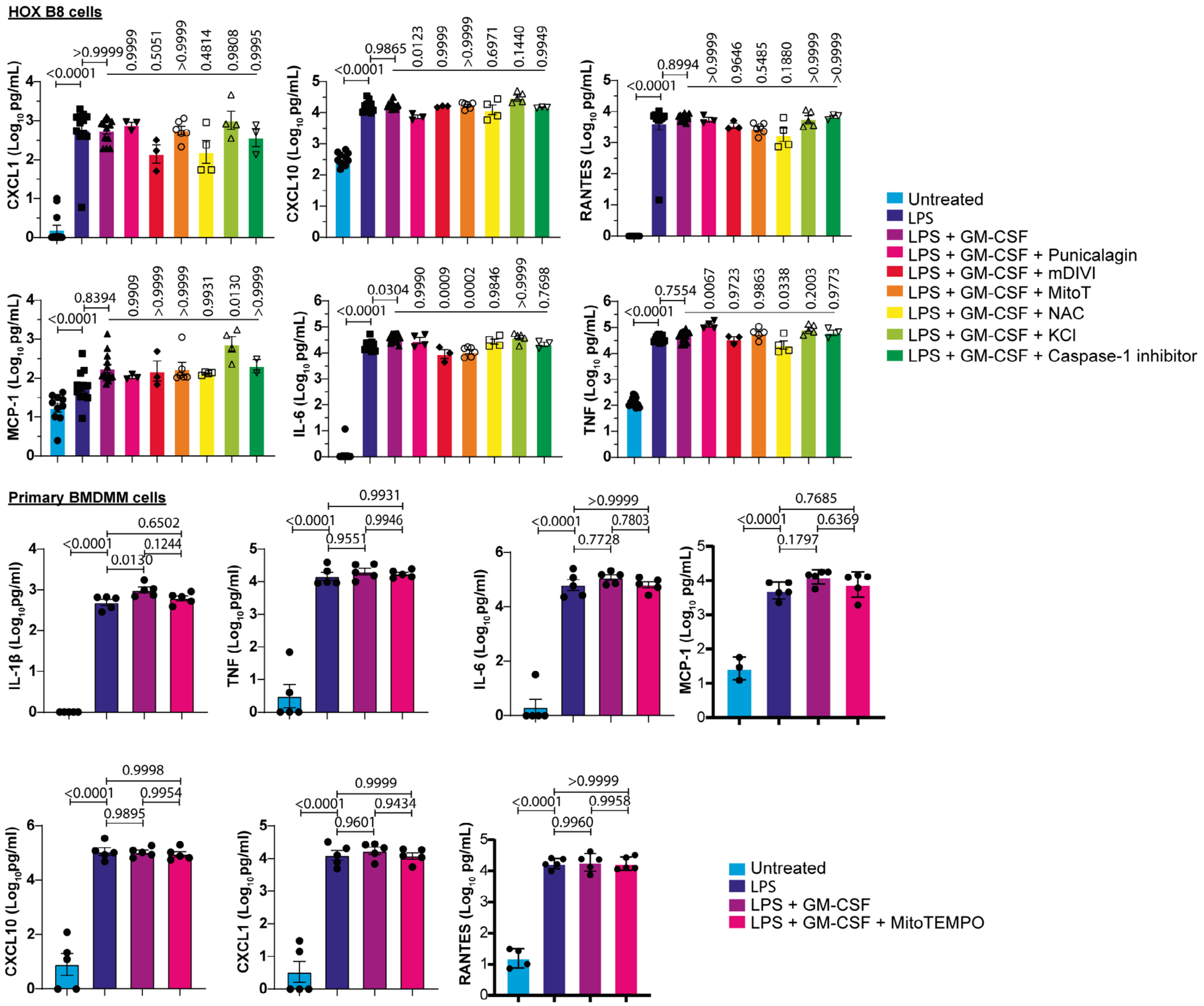

Figure EV3. LPS and GM-CSF treatment induce a pro-inflammatory profile.

HoxB8 macrophages or BMDMM were treated for 16 h as indicated. The indicated cytokines were analyzed in supernatants by LEGENDplex antiviral response assay as indicated in the Materials and Methods. Shown are the concentrations (pg/ml) converted into $\log_{10}$ in order to be following a normal Gaussian distribution for statistical analysis.

Data information: $n \geq 3$ biological replicates (every dot represents one biological replicate). For all panels, error bars are SEM. Significance was calculated using one-way ANOVA with multiple comparisons. *P*-values are indicated.

© 2022 The Authors

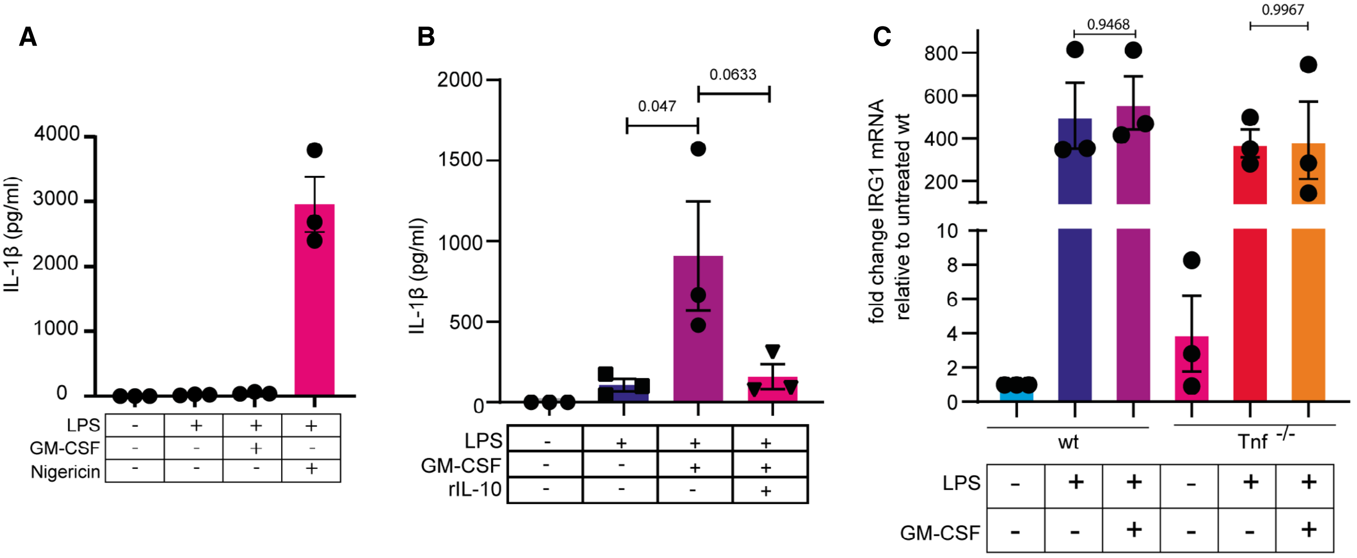

Figure EV4. GM-CSF-induced IL-1β secretion is blocked by IL-10 but does not require IRG1 dysregulation.

A BMDM were generated as described in Materials and Methods and treated as indicated for 16 h. IL-1β levels were measured in media by ELISA.

B HoxB8 macrophages were treated as indicated with or without recombinant IL-10 and secreted IL-1β levels were measured using ELISA.

C Wild-type and $Tnf^{-/-}$ HoxB8 macrophages were treated as indicated for 12 h, and RNA was extracted. Levels of IRG1 were analyzed by qPCR.

Data information: $n \geq 3$ biological replicates (every dot represents one biological replicate). For all panels, error bars are SEM. Significance was calculated using two-way ANOVA with multiple comparisons. *P*-values are shown.

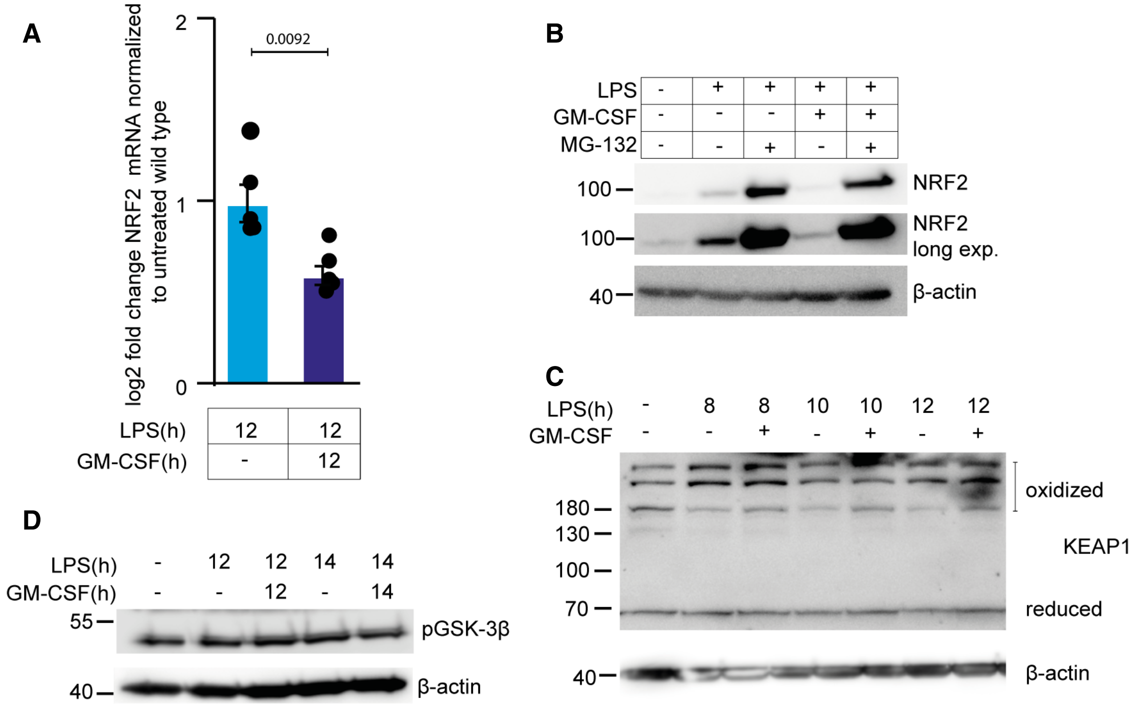

Figure EV5. GM-CSF-regulated NRF2 stability independently of GSK3β.

A HoxB8 macrophages were treated for 12 h with LPS or LPS + GM-CSF and RNA was extracted and analyzed by qPCR for NRF2 mRNA.

B HoxB8 macrophages were treated for 12 h ± GM-CSF. MG132 was added for 1 h prior to cells being lysed and analyzed for levels of NRF2 by western blot.

C HoxB8 macrophages were treated for the indicated times with LPS or LPS + GM-CSF and proteins extracted and western blots made against KEAP1. High-molecular-weight bands represent oxidized KEAP1.

D HoxB8 macrophages were treated for 12 or 14 h with LPS or LPS + GM-CSF. Proteins were analyzed by western blot for levels of phospho-GSK3β.

Data information: $n \geq 3$ biological replicates (every dot represents one biological replicate). Error bars are SEM and *P*-values were calculated using unpaired *t*-tests. Westerns are representative images from three biological replicates.
Source data are available online for this figure.

 © 2022 The Authors
