# Supplementary material for: GM‐CSF suppresses antioxidant signaling and drives IL‐1β secretion through NRF2 downregulation
Source: EMBO Rep. 2022 Jun 13;23(8):e54226. doi: 10.15252/embr.202154226 (PMC9346485; doi:10.15252/embr.202154226)

EV2E

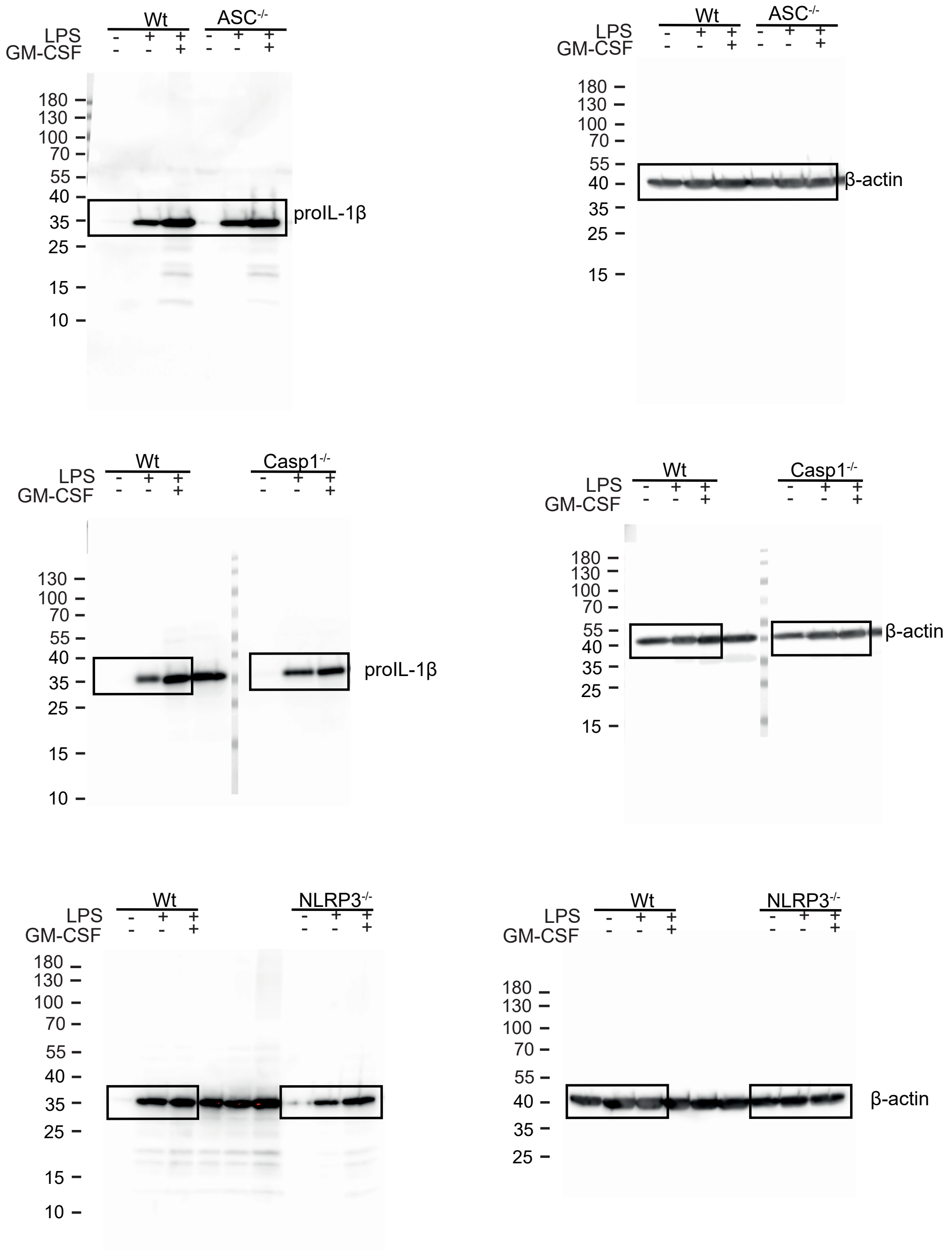
Wt
ASC-/-
LPS
GM-CSF
180
130
100
70
55
40
35
25
15
10
proIL-1β
β-actin
Casp1-/-
NLRP3-/-

EV5B

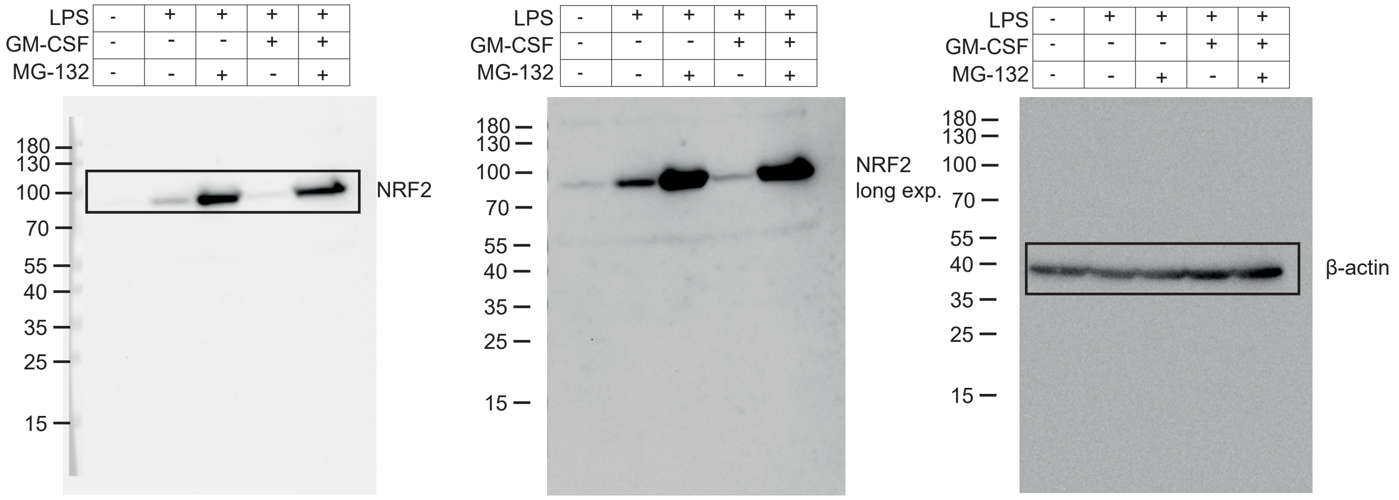

EV5C

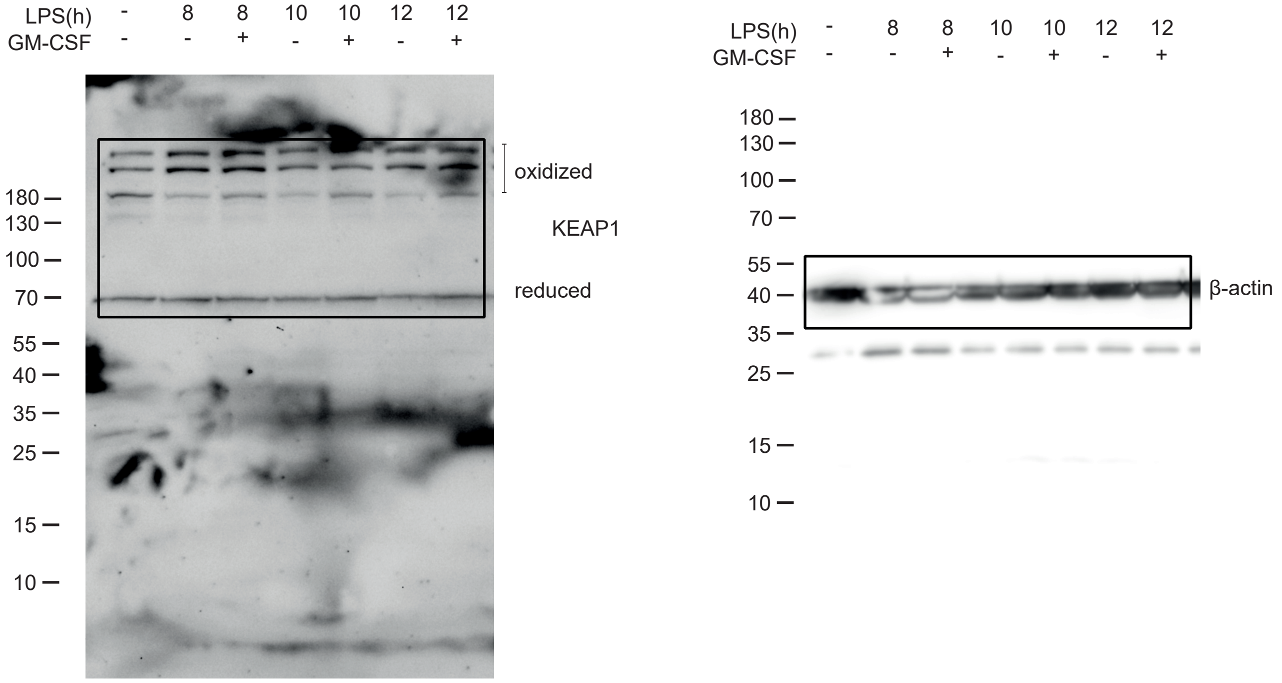

EV5D

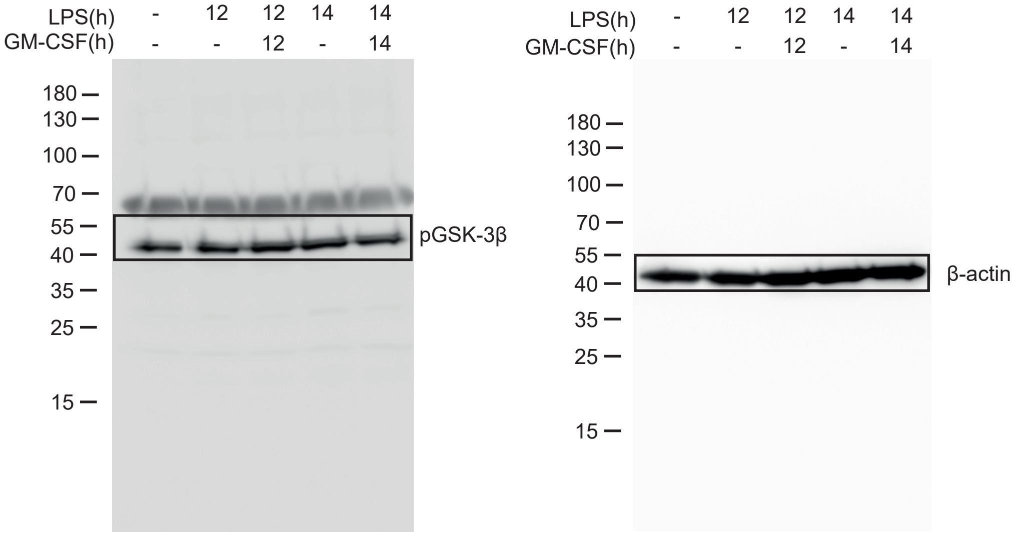

Supplement: Supplementary file 2 — Source Data for Expanded View [file EMBR-23-e54226-s004.pdf]
