# Supplementary figures and images for: GM‐CSF suppresses antioxidant signaling and drives IL‐1β secretion through NRF2 downregulation
Source: EMBO Rep. 2022 Jun 13;23(8):e54226. doi: 10.15252/embr.202154226 (PMC9346485; doi:10.15252/embr.202154226)

1B

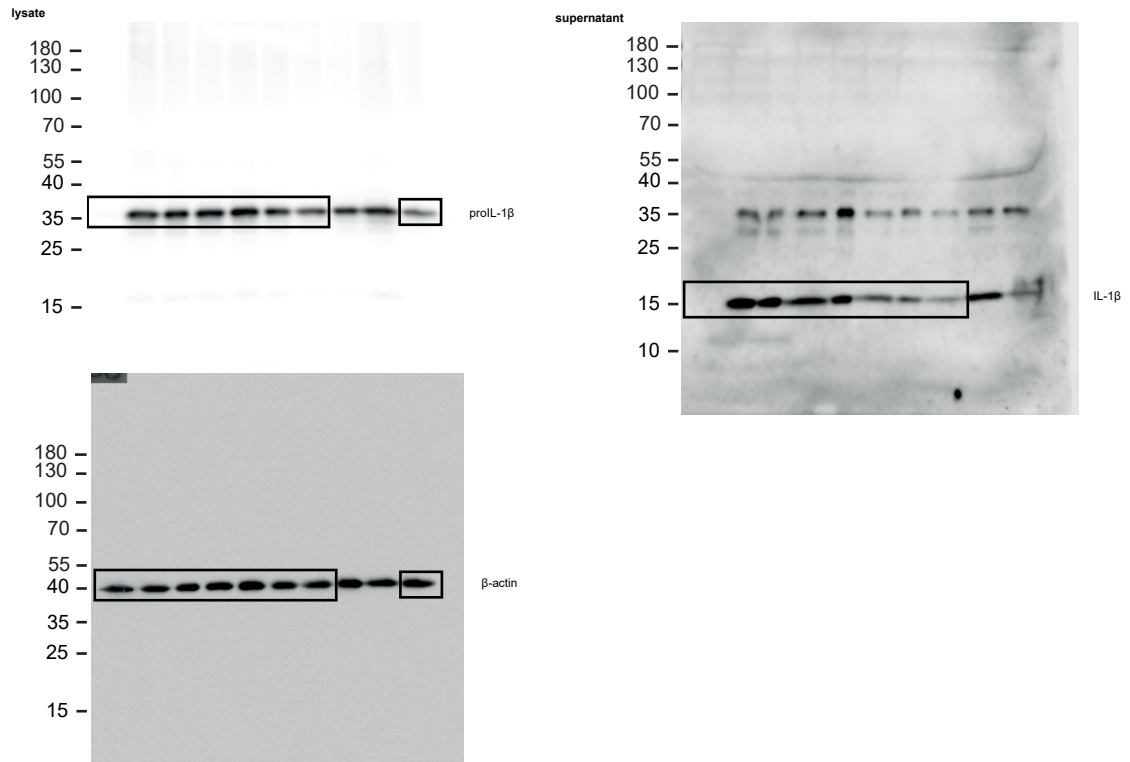

1C

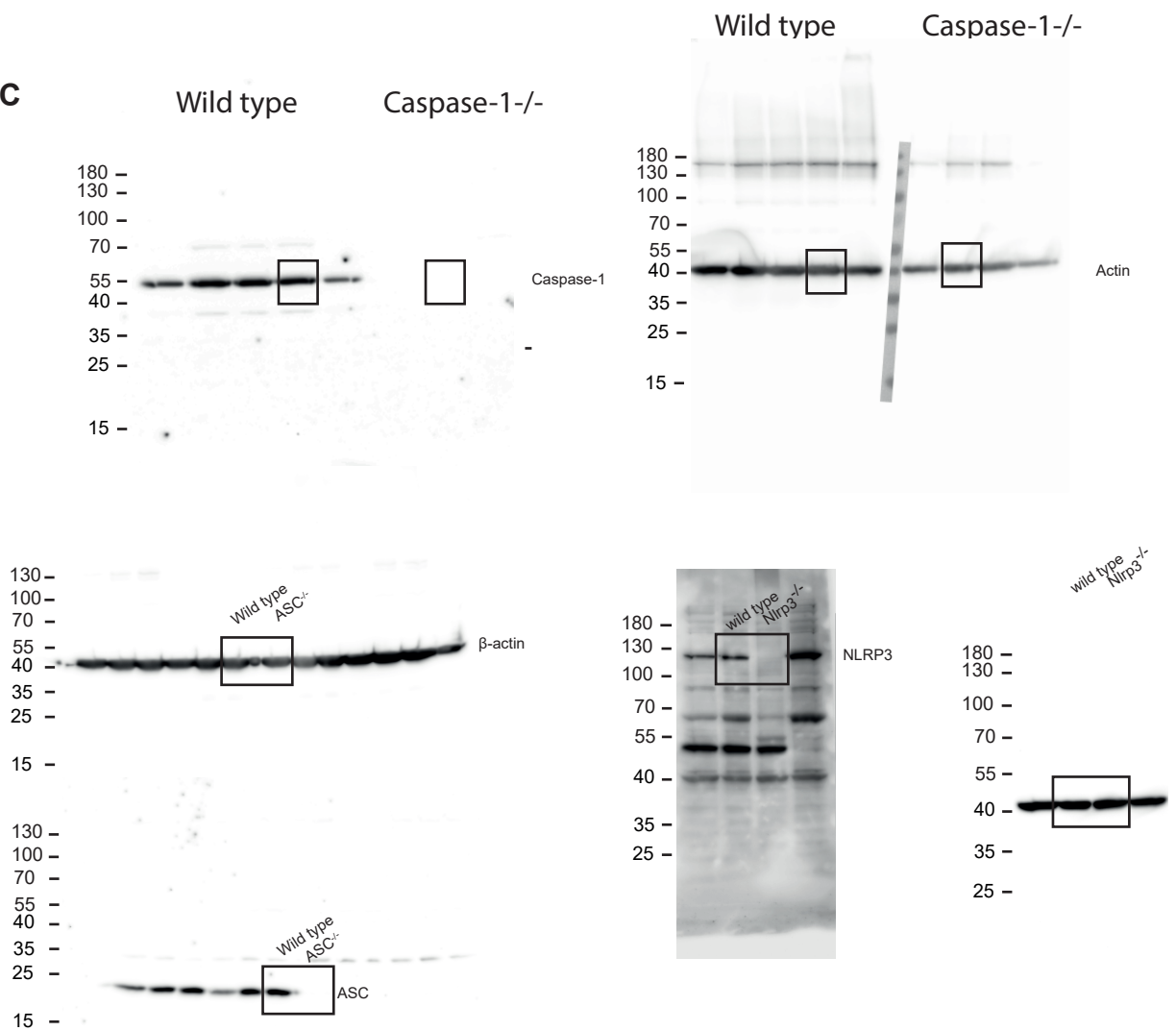

Supplement: Supplementary file 3 — Source Data for Figure 1 [file EMBR-23-e54226-s005.pdf]

4A

BMDMM

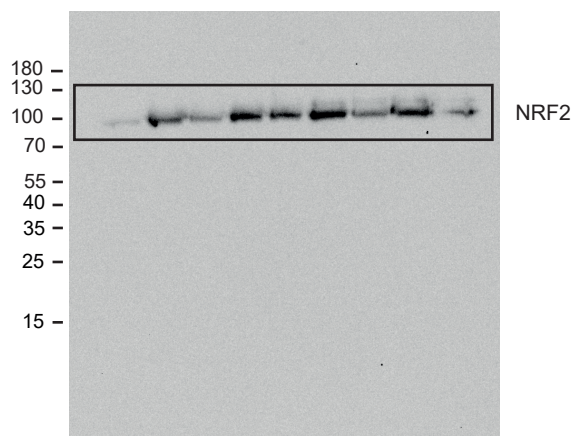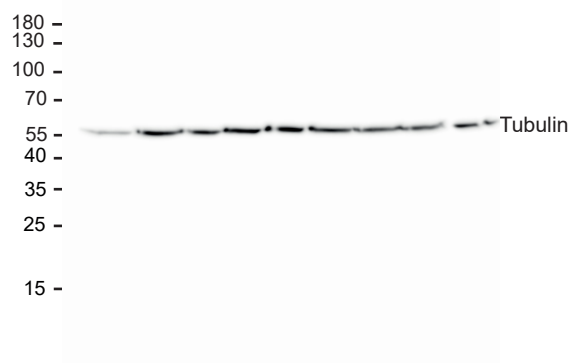

HoxB8

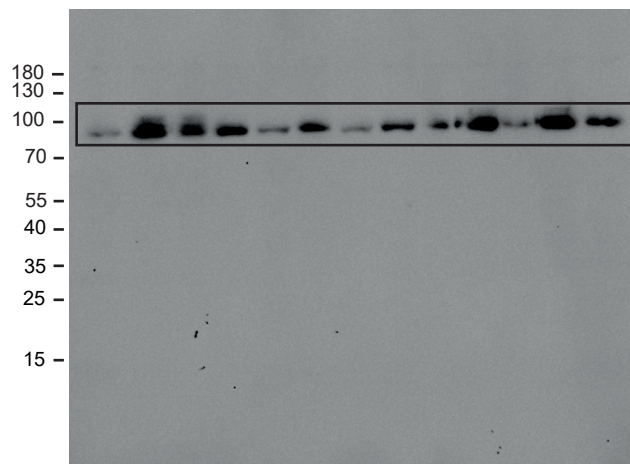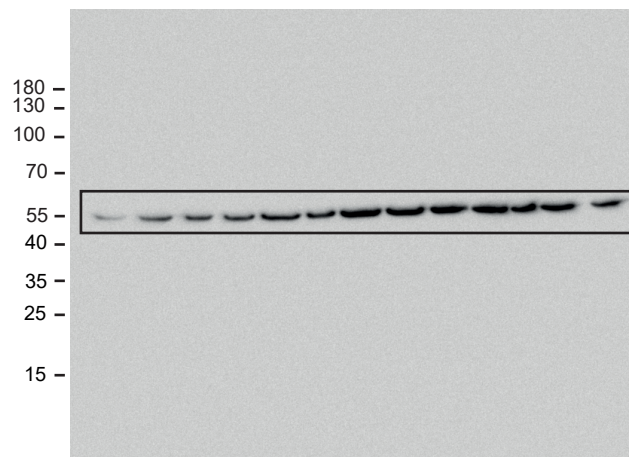

4D

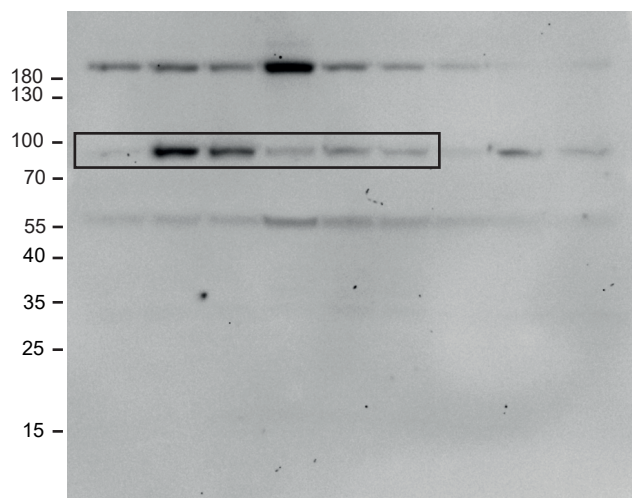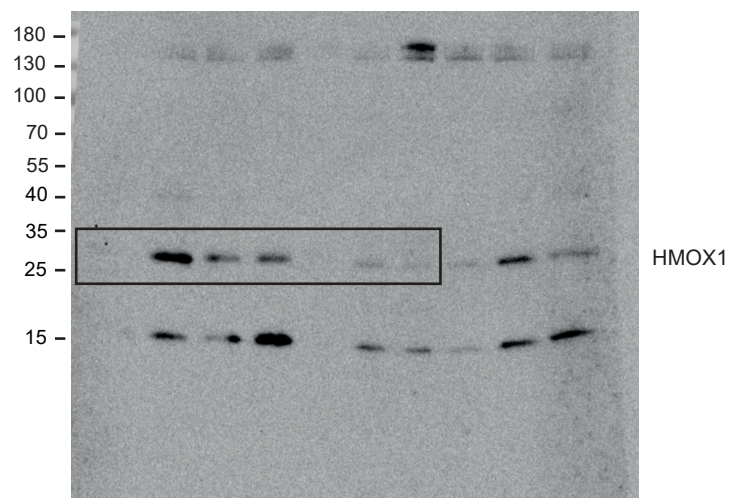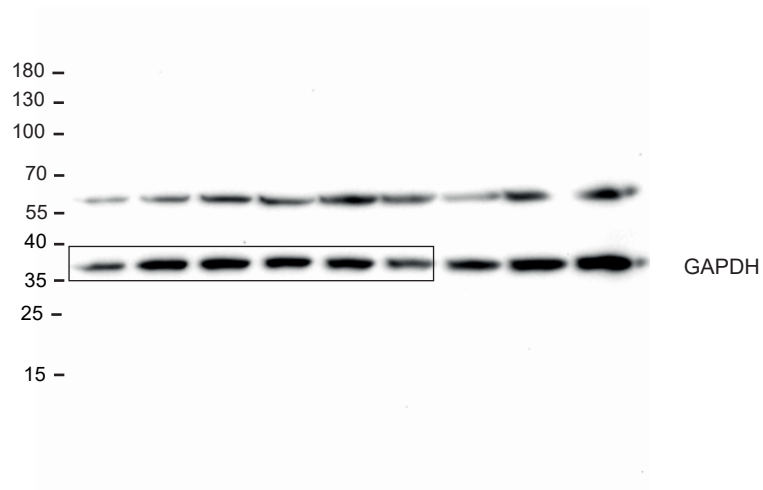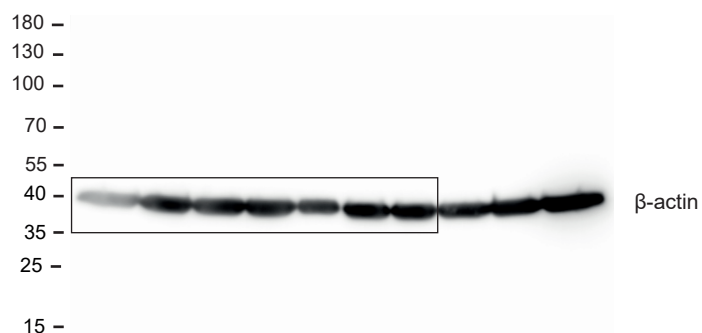

4F

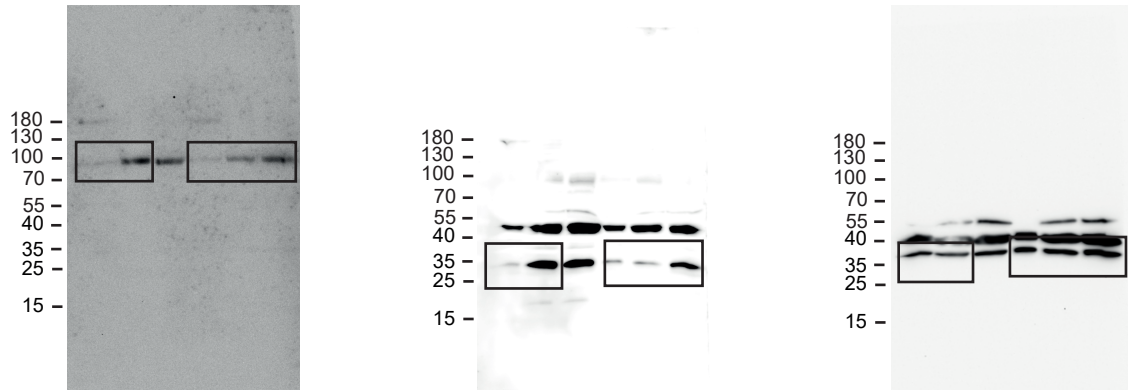

Supplement: Supplementary file 4 — Source Data for Figure 4 [file EMBR-23-e54226-s007.pdf]

5B

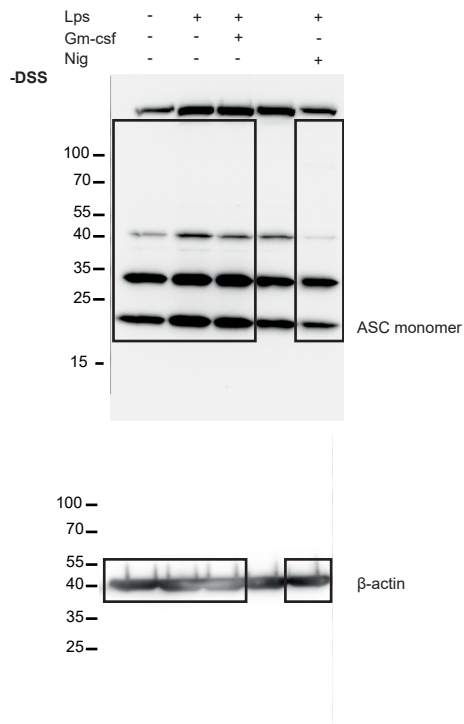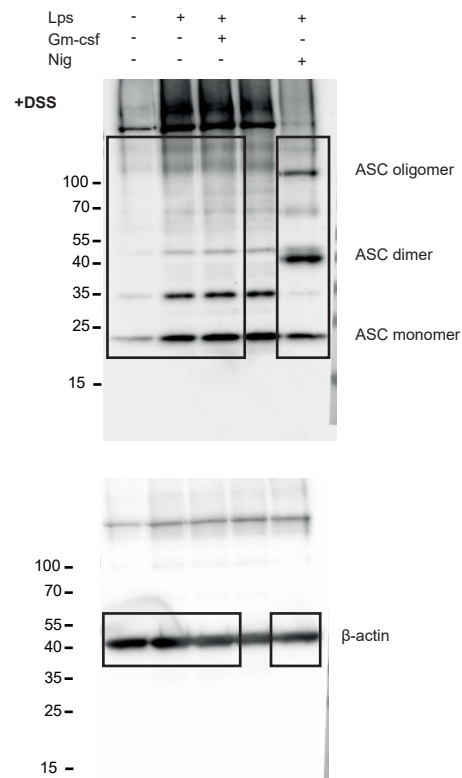

5C

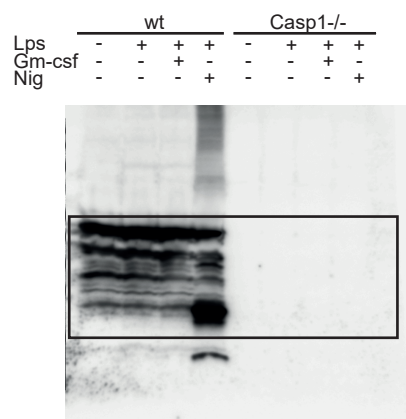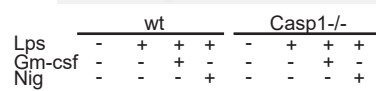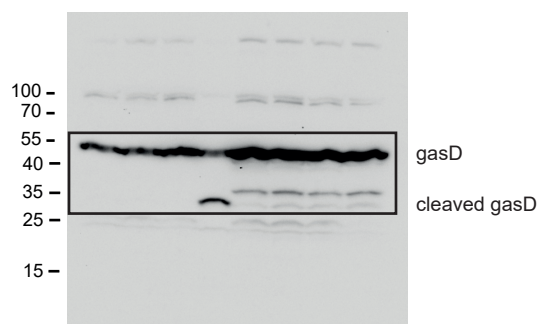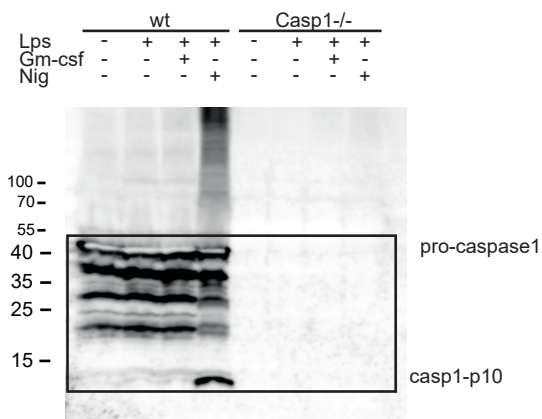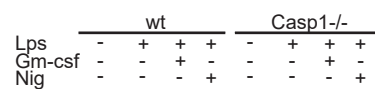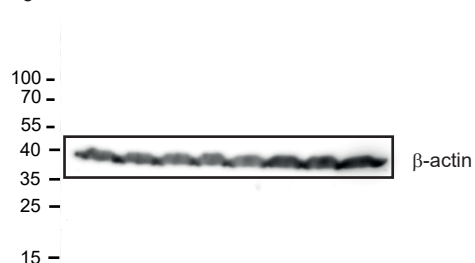

5D

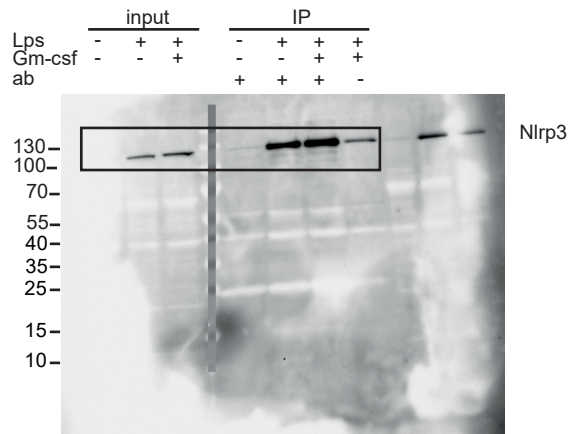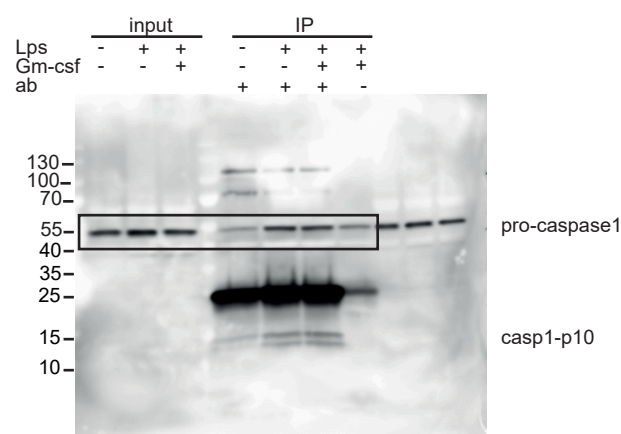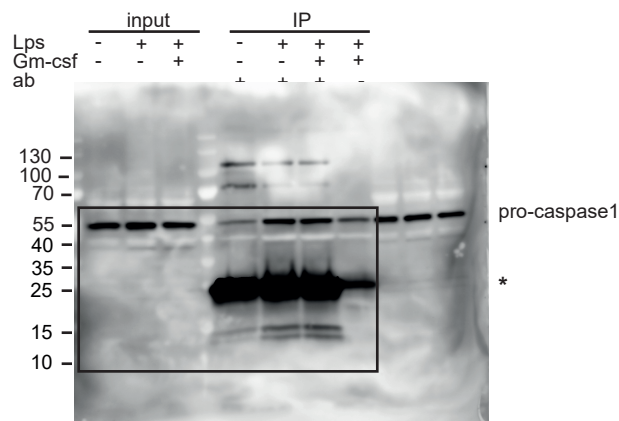

5E

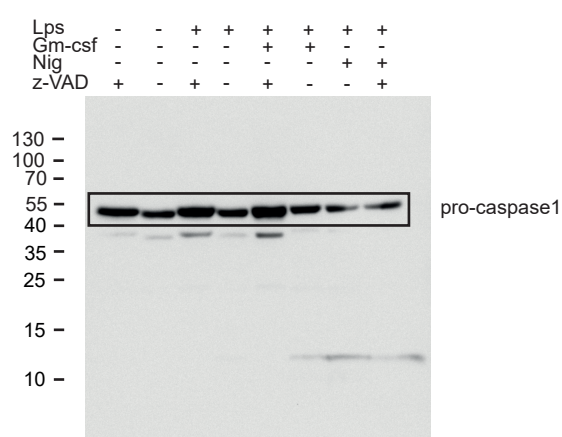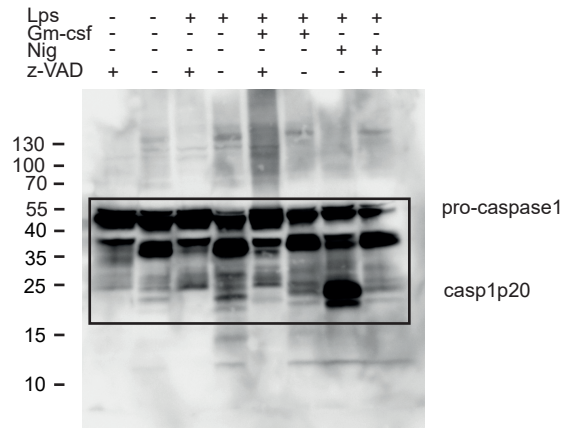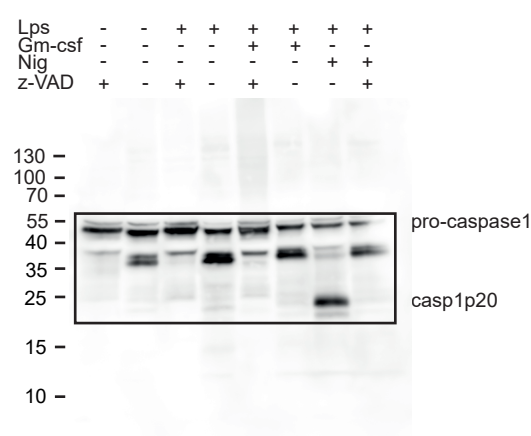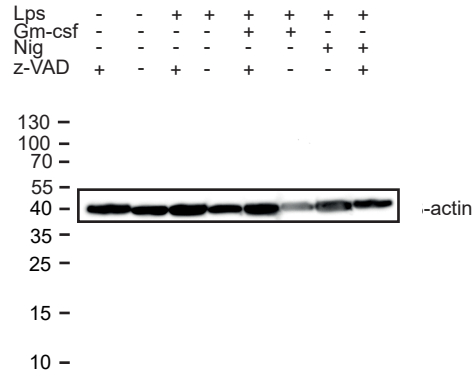

Supplement: Supplementary file 5 — Source Data for Figure 5 [file EMBR-23-e54226-s008.pdf]

7A

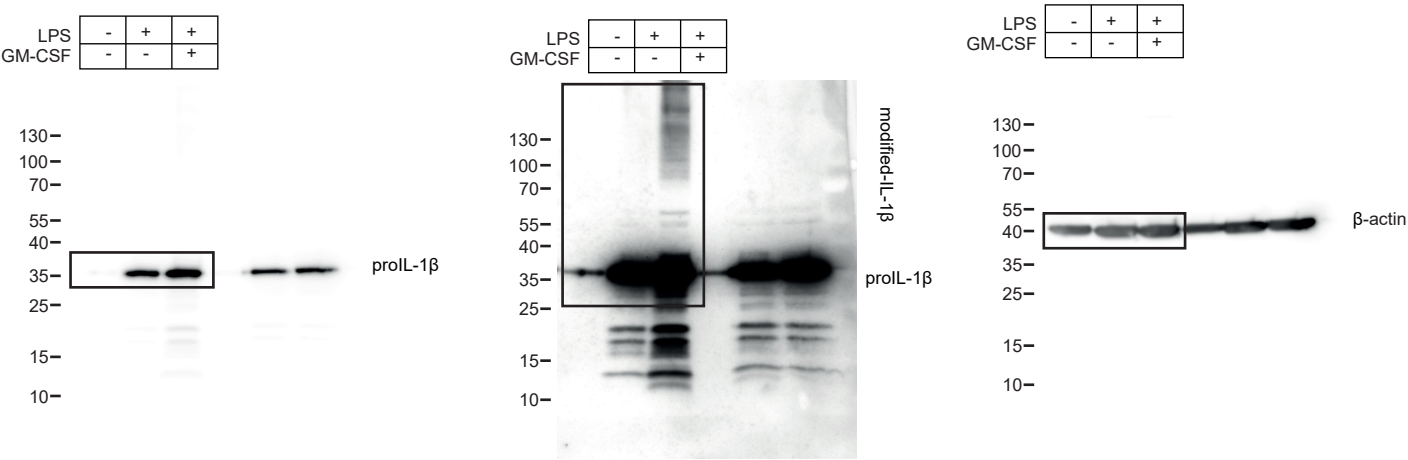

7B

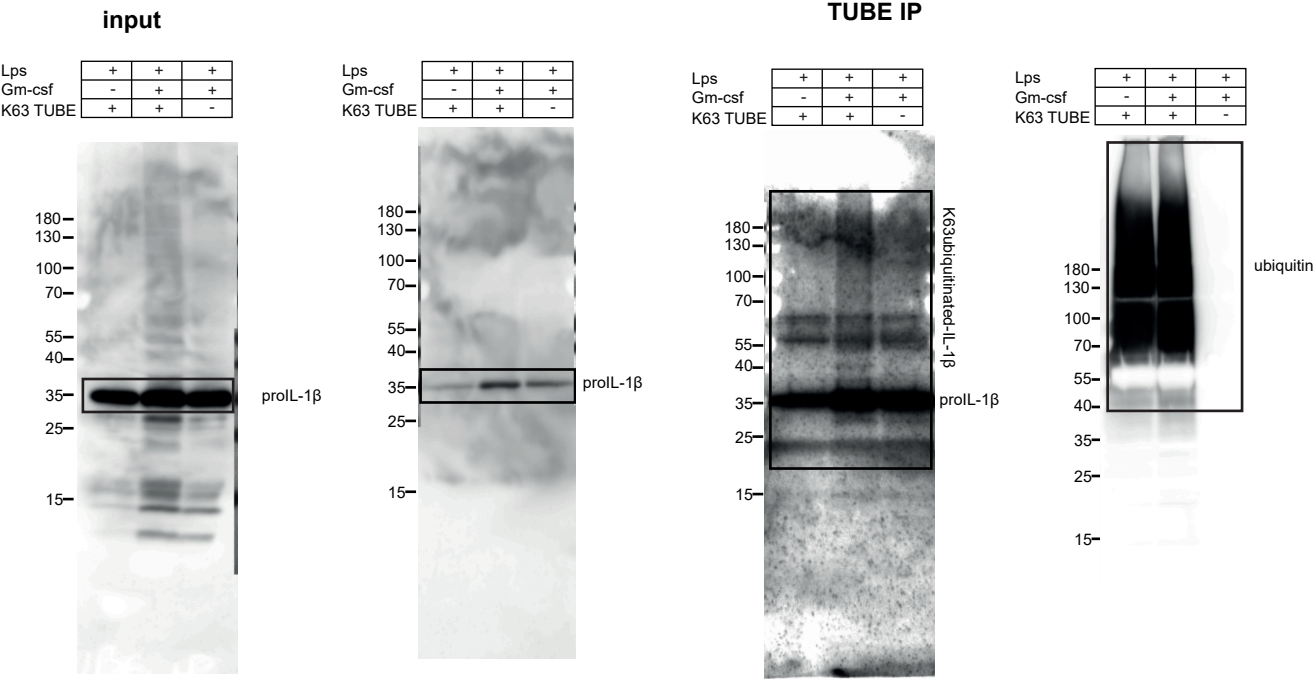

7D

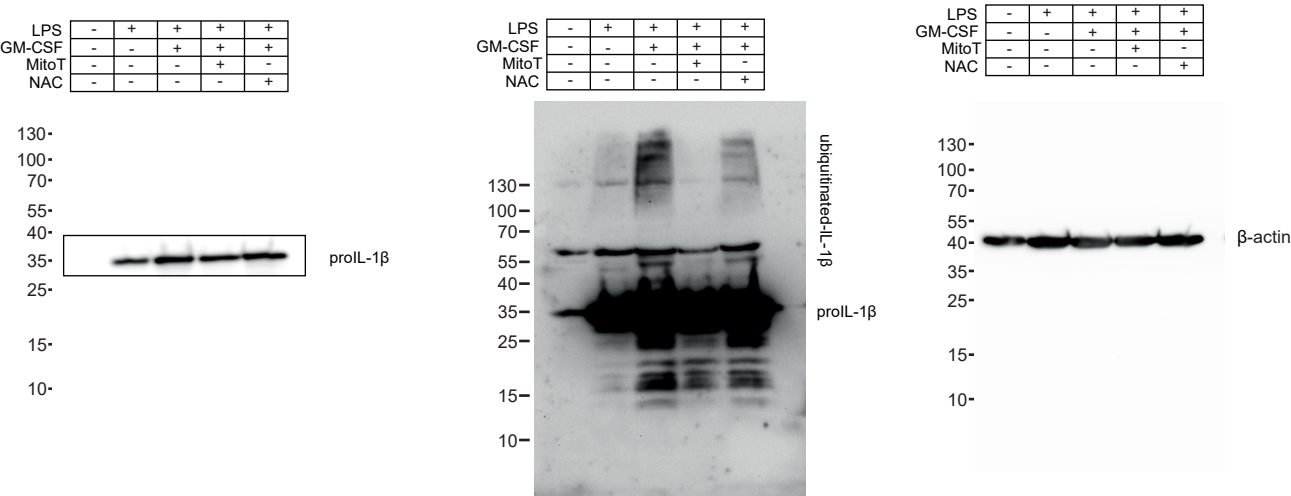

7E

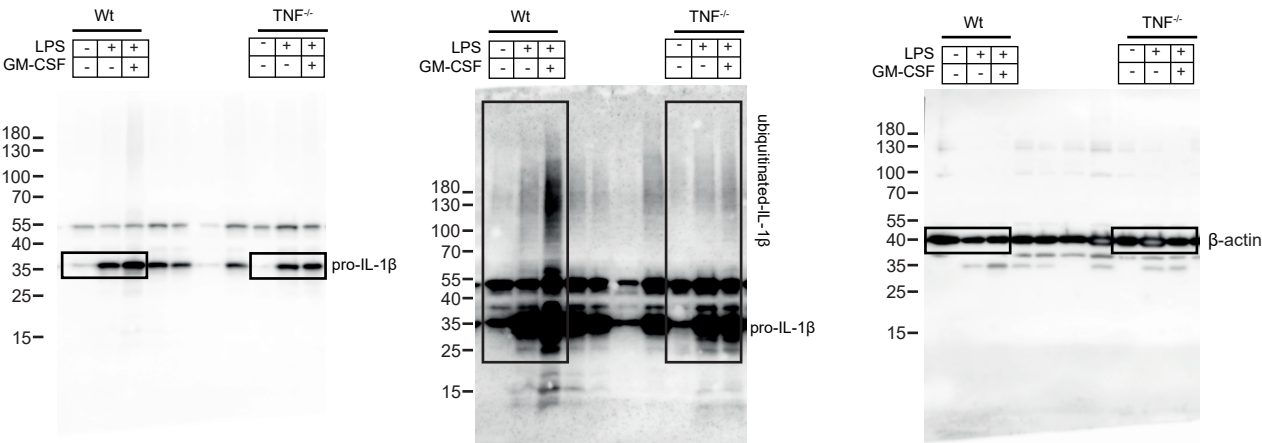

7F

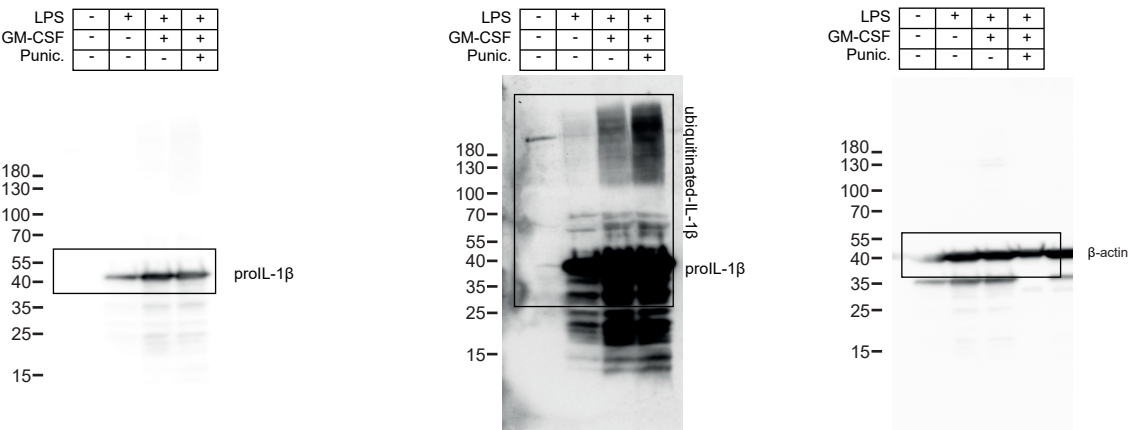

7C

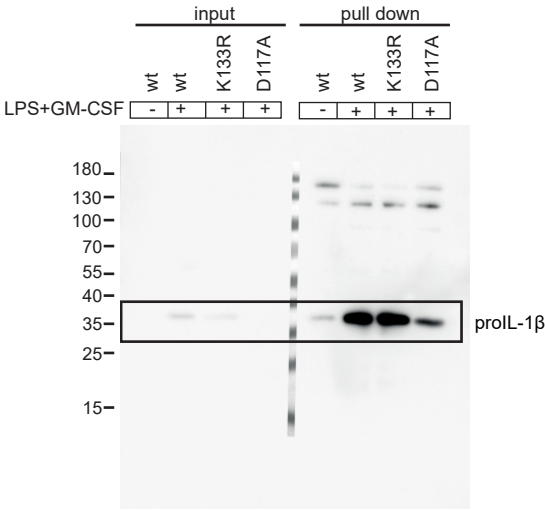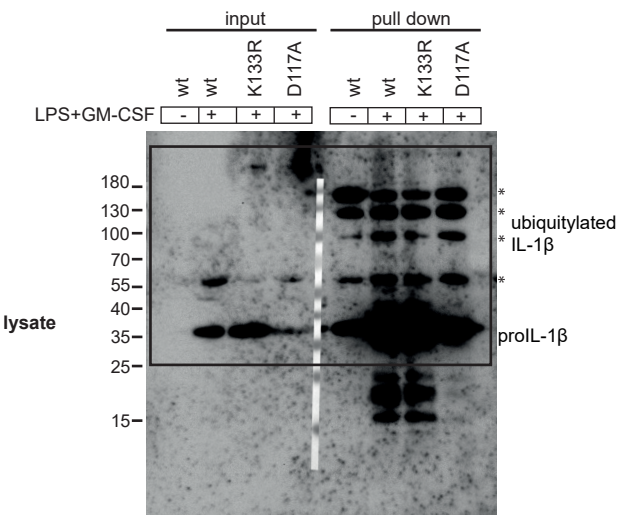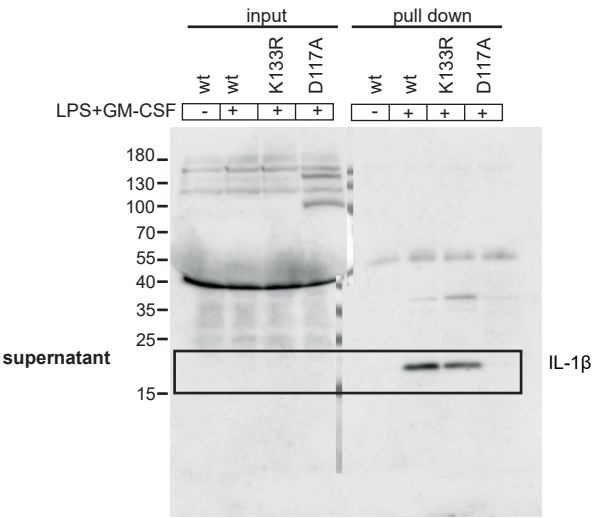

Supplement: Supplementary file 6 — Source Data for Figure 7 [file EMBR-23-e54226-s006.pdf]

8E

|        |   |   |   |   |   |   |
|--------|---|---|---|---|---|---|
| LPS    | - | + | + | - | + | + |
| GM-CSF | - | - | + | - | - | + |

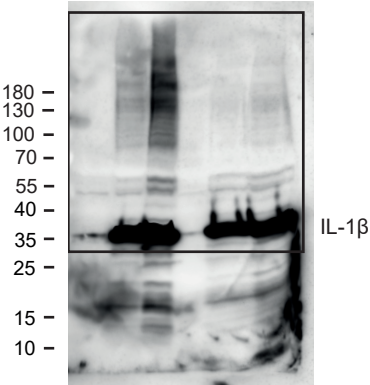

|        |   |   |   |   |   |   |
|--------|---|---|---|---|---|---|
| LPS    | - | + | + | - | + | + |
| GM-CSF | - | - | + | - | - | + |

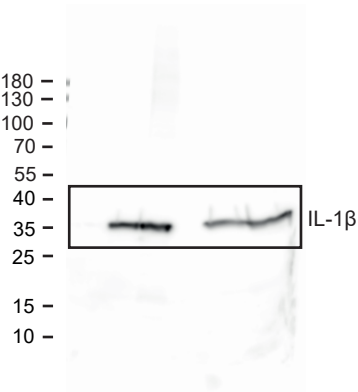

|        |   |   |   |   |   |   |
|--------|---|---|---|---|---|---|
| LPS    | - | + | + | - | + | + |
| GM-CSF | - | - | + | - | - | + |

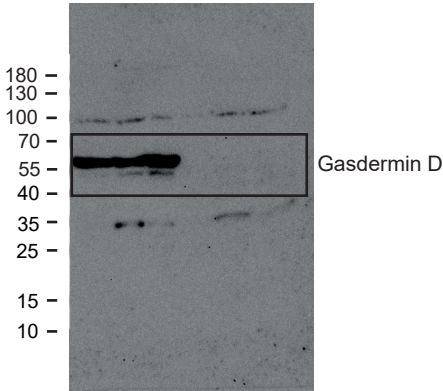

|        |   |   |   |   |   |   |
|--------|---|---|---|---|---|---|
| LPS    | - | + | + | - | + | + |
| GM-CSF | - | - | + | - | - | + |

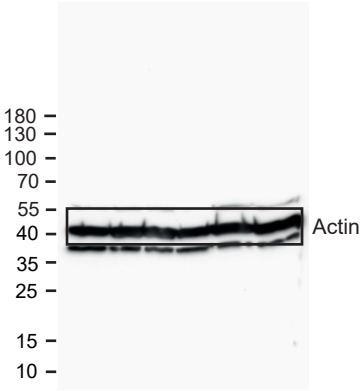

Supplement: Supplementary file 7 — Source Data for Figure 8 [file EMBR-23-e54226-s001.pdf]
