# Supplementary material for: GM‐CSF suppresses antioxidant signaling and drives IL‐1β secretion through NRF2 downregulation
Source: EMBO Rep. 2022 Jun 13;23(8):e54226. doi: 10.15252/embr.202154226 (PMC9346485; doi:10.15252/embr.202154226)
Supplement: Supplementary file 8 [file EMBR-23-e54226-s003.pdf]

EMBO reports

GM-CSF suppresses antioxidant signaling and drives IL-1β secretion through NRF2 downregulation

Sara Di Carlo, Georg Häcker, and Ian Gentle
DOI: 10.15252/embr.202154226

Corresponding author(s): Ian Gentle (ian.gentle@uniklinik-freiburg.de)

Review Timeline:

Submission Date:	26th Oct 21
Editorial Decision:	22nd Nov 21
Revision Received:	22nd Mar 22
Editorial Decision:	12th Apr 22
Revision Received:	11th May 22
Accepted:	24th May 22

Editor: Achim Breiling

Transaction Report:

(Note: With the exception of the correction of typographical or spelling errors that could be a source of ambiguity, letters and reports are not edited. Depending on transfer agreements, referee reports obtained elsewhere may or may not be included in this compilation. Referee reports are anonymous unless the Referee chooses to sign their reports.)

Dear Dr. Gentle,

Thank you for the submission of your research manuscript to EMBO reports. We have now received the reports from the three referees that were asked to evaluate your study, which can be found at the end of this email.

As you will see, the referees think that these findings are of interest. However, they have several comments, concerns and suggestions, indicating that a major revision of the manuscript is necessary to allow publication of the study in EMBO reports. As the reports are below, and all their points need to be addressed, I will not detail them here.

Given the constructive referee comments, we would like to invite you to revise your manuscript with the understanding that all referee concerns must be addressed in the revised manuscript or in the detailed point-by-point response. Acceptance of your manuscript will depend on a positive outcome of a second round of review. It is EMBO reports policy to allow a single round of revision only and acceptance of the manuscript will therefore depend on the completeness of your responses included in the next, final version of the manuscript.

Revised manuscripts should be submitted within three months of a request for revision. Please contact me to discuss the revision should you need additional time.

When submitting your revised manuscript, please also carefully review the instructions that follow below.

PLEASE NOTE THAT upon resubmission revised manuscripts are subjected to an initial quality control prior to exposition to re-review. Upon failure in the initial quality control, the manuscripts are sent back to the authors, which may lead to delays. Frequent reasons for such a failure are the lack of the data availability section (please see below) and the presence of statistics based on n=2 (the authors are then asked to present scatter plots or provide more data points).

When submitting your revised manuscript, we will require:

1) a .docx formatted version of the final manuscript text (including legends for main figures, EV figures and tables), but without the figures included. Please make sure that changes are highlighted to be clearly visible. Figure legends should be compiled at the end of the manuscript text.

2) individual production quality figure files as .eps, .tif, .jpg (one file per figure), of main figures and EV figures. Please upload these as separate, individual files upon re-submission.

The Expanded View format, which will be displayed in the main HTML of the paper in a collapsible format, has replaced the Supplementary information. You can submit up to 5 images as Expanded View. Please follow the nomenclature Figure EV1, Figure EV2 etc. The figure legend for these should be included in the main manuscript document file in a section called Expanded View Figure Legends after the main Figure Legends section. Additional Supplementary material should be supplied as a single pdf file labeled Appendix. The Appendix should have page numbers and needs to include a table of content on the first page (with page numbers) and legends for all content. Please follow the nomenclature Appendix Figure Sx, Appendix Table Sx etc. throughout the text, and also label the figures and tables according to this nomenclature.

For more details, please refer to our guide to authors:
http://www.embopress.org/page/journal/14693178/authorguide#manuscriptpreparation

Please consult our guide for figure preparation:
http://wol-prod-cdn.literatumonline.com/pb-assets/embo-site/EMBOPress_Figure_Guidelines_061115-1561436025777.pdf

See also the guidelines for figure legend preparation:
https://www.embopress.org/page/journal/14693178/authorguide#figureformat

3) a .docx formatted letter INCLUDING the reviewers' reports and your detailed point-by-point responses to their comments. As part of the EMBO Press transparent editorial process, the point-by-point response is part of the Review Process File (RPF), which will be published alongside your paper.

4) a complete author checklist, which you can download from our author guidelines (https://www.embopress.org/page/journal/14693178/authorguide). Please insert page numbers in the checklist to indicate where the requested information can be found in the manuscript. The completed author checklist will also be part of the RPF.

Please also follow our guidelines for the use of living organisms, and the respective reporting guidelines:
http://www.embopress.org/page/journal/14693178/authorguide#livingorganisms

5) that primary datasets produced in this study (e.g. RNA-seq, ChIP-seq, structural and array data) are deposited in an appropriate public database. If no primary datasets have been deposited, please also state this a dedicated section (e.g. 'No primary datasets have been generated and deposited'), see below.

See also: http://embor.embopress.org/authorguide#datadeposition

Please remember to provide a reviewer password if the datasets are not yet public.

The accession numbers and database should be listed in a formal "Data Availability " section (placed after Materials & Methods) that follows the model below. This is now mandatory (like the COI statement). Please note that the Data Availability Section is restricted to new primary data that are part of this study.

Data availability

The datasets produced in this study are available in the following databases:

- RNA-Seq data: Gene Expression Omnibus GSE46843 (https://www.ncbi.nlm.nih.gov/geo/query/acc.cgi?acc=GSE46843)
- [data type]: [name of the resource] [accession number/identifier/doi] ([URL or identifiers.org/DATABASE:ACCESSION])

*** Note - All links should resolve to a page where the data can be accessed. ***

Moreover, I have these editorial requests:

6) We strongly encourage the publication of original source data with the aim of making primary data more accessible and transparent to the reader. The source data will be published in a separate source data file online along with the accepted manuscript and will be linked to the relevant figure. If you would like to use this opportunity, please submit the source data (for example scans of entire gels or blots, data points of graphs in an excel sheet, additional images, etc.) of your key experiments together with the revised manuscript. If you want to provide source data, please include size markers for scans of entire gels, label the scans with figure and panel number, and send one PDF file per figure.

7) Our journal encourages inclusion of *data citations in the reference list* to directly cite datasets that were re-used and obtained from public databases. Data citations in the article text are distinct from normal bibliographical citations and should directly link to the database records from which the data can be accessed. In the main text, data citations are formatted as follows: "Data ref: Smith et al, 2001" or "Data ref: NCBI Sequence Read Archive PRJNA342805, 2017". In the Reference list, data citations must be labeled with "[DATASET]". A data reference must provide the database name, accession number/identifiers and a resolvable link to the landing page from which the data can be accessed at the end of the reference. Further instructions are available at: http://www.embopress.org/page/journal/14693178/authorguide#referencesformat

8) Regarding data quantification and statistics, can you please specify, where applicable, the number "n" for how many independent experiments were performed, if these were biological or technical replicates, the bars and error bars (e.g. SEM, SD) and the test used to calculate p-values in the respective figure legends. Please provide statistical testing where applicable, and also add a paragraph detailing this to the methods section. See:
http://www.embopress.org/page/journal/14693178/authorguide#statisticalanalysis

9) Please also note our reference format:
http://www.embopress.org/page/journal/14693178/authorguide#referencesformat

10) For microscopic images, please add scale bars of similar style and thickness to all the microscopic images, using clearly visible black or white bars (depending on the background). Please place these in the lower right corner of the images. Please do not write on or near the bars in the image but define the size in the respective figure legend.

11) Please order the manuscript sections like this:
Title page - Abstract - Introduction - Results - Discussion - Materials and Methods - DAS - Acknowledgements - Author contributions - Conflict of interest - References - Figure legends - Expanded View Figure legends

I look forward to seeing a revised version of your manuscript when it is ready. Please let me know if you have questions or comments regarding the revision.

Please use this link to submit your revision: https://embor.msubmit.net/cgi-bin/main.plex

Yours sincerely,

Achim Breiling
Editor

Referee #1:

General Comments: This MS describes novel and significant observations regarding signaling mechanisms by which GM-CSF can synergize with LPS to drive slow but sustained NLRP3/caspase-1-dependent proteolytic processing and non-lytic secretion of IL-1beta from murine bone marrow-derived monocytic macrophages. The authors present compelling data that supports an atypical pathway for processing and release of IL-1beta that includes: 1) LPS induced production of autocrine TNFa; 2) TNFa induction of mitochondrial ROS production; 3) GM-CSF-mediated suppression of the LPS-driven up-regulation of the Nrf2 master anti-oxidant protein; 4) ROS-dependent potentiation of an NLRP3/ASC/caspase-1 signaling axis which does NOT involve stable assembly of large NLRP3/ASC inflammasome signaling platforms; 5) sufficient caspase-1 activity to drive the proteolytic processing of proIL-1beta to mature IL-1b; 6) a GM-CSF-dependent increase in K63-ubiquitination of proIL-1beta which further enhances its proteolytic processing by caspase-1; 7) an atypical pathway for secretion/ release of the mature IL-1beta that is dependent on Gasdermin D (GSDMD) but apparently independent of GSDMD-proteolytic cleavage, GSDMD-pore-driven plasma membrane permeabilization and consequent pyroptosis. In general, the experiments are well-designed and thoughtfully interpreted. However, some additional experiments and discussion related to the atypical mechanism(s) underlying the observed IL-1beta secretion are required. A few other technical issues require clarification.

Specific Comments:

1. The authors cite Monteleone et al 2018 as background for how GSDMD pores can mediate slow IL-1beta release in the absence of pyroptotic cell death. However, the SyTox green uptake experiments (Fig 3D) indicate that no plasma membrane pores accumulate during LPS/GM-CSF stimulation of the HoxB8 macrophages. If GSDMD pores accumulated even transiently in low numbers and are countered by robust membrane repair, the SyTox influx through the non-lytic pores will intercalate with cellular DNA and irreversibly (on the time scale of these experiments) mark those cells with fluorescent SyTox/DNA complexes. Thus, IL-1beta efflux through non-lytic plasma membrane GSDMD pores is an unlikely mechanism. Rather, it seems that GSDMD is regulating an alternative IL-1b release that may involve trafficking to a vesicular export pathway (exosomes? secretory autophagosomes?) in these bone marrow monocyte-derived macrophages models. Several recent studies have described such pathways in other myeloid or non-myeloid cell models (Karmakar et al. 2020. PMID32371889; Kimura et al. 2017, PMID 27932448). The authors should evaluate whether some of these alternative pathways are involved in the observed IL-1beta secretion in their experiments. This is an important mechanistic question.

2.Fig 4: The authors should indicate whether exogenous TNFalpha "rescues" the LPS-induced accumulation of Nrf2 in the TNF-knockout macrophages.

3. Fig 5E: The authors should discuss the significance of the 35 kDa caspase-1 band observed in non-stimulated cells (lane 2).

4. Fig. 5: Prolonged treatment of most macrophages with LPS and the zVAD pan-caspase inhibitor induces necroptosis due the suppression of caspase-8 which otherwise restrains RIP1 and RIP3 activation. This should be evaluated/ considered in this experimental model.

Referee #2:

In the current manuscript, the authors describe that GM-CSF induces Nlrp3-dependent Il1b secretion in macrophages in vitro mediated by a ROS-dependent pathway, suppression of Nrf2 and post-translational modifications of Il1b.

The manuscript demonstrates a well constructed series of experiments with an interesting finding. However, several issues need be addressed, and experiments included that would strengthen their claim.

Key points:

Throughout the paper, secreted IL-1b is measured and the implication is that the effects of LPS/GM-CSF and various other reagents added to cell cultures which inhibit IL-1b secretion are, in fact, specific to IL-1b release. The authors should add experiments aimed at de-risking the possibility of a broader inhibition of secretory proteins by these various reagents may be taking place. This is necessary to strengthen the connection between the proposed mechanism and the results provided.
To further elaborate with an example, in Figure 2, the authors claim that LPS stimulated HoxB8 macrophages produce mitochondrial ROS, and that these ROS are driving IL-1β secretion.

Small molecules can have many unidentified off target effects. 25 uM is a high concentration for 16h incubation periods and there is not much literature on Mdivi-1, so I recommend strengthening this point with some additional experiments. The awareness to avoid undesired toxicity of small molecules is appreciated, but this is not the only class of off-target effects that

can be observed or make interpretation of results challenging.

The authors should show that secretion of other proinflammatory proteins is not inhibited (in your assay set ups) by the compounds used, ideally using a dose response strategy that covers the concentration used in the manuscript (for example, the 25uM MDIVI-1 or 50mM KCl). This should be considered for all compounds used that showed an increase or decrease of IL-1b secretion.

Perhaps a multiplex proinflammatory cytokine/chemokine assay on supernatants from LPS/GM-CSF-stimulated Hoxb8 and BMDMMs to show that indeed the reduction is either specific to, or enhanced for, IL-1b secretion.

Compounds to consider for this experiments include:
CRT0066101
NAP
CCCP
MDIVI-1
Punicalagin
KCl

If a claim is to be made that a mechanism influencing IL-1b secretion is discovered, it should ideally be shown in context of other cytokines and secreted proteins - namely that they are-or-are-not equally affected.

Perhaps the GM-CSF/NRF2 axis is of broader importance? This should be clarified.

Additional comments:

- Figure 4 - Whilst the impact of GM-CSF in NRF2 is clear and a key result in the manuscript is observable.... the "two waves" described in the text relating to Fig 4A seem to be influenced by less protein abundance at the 8h time point (at least in the Hoxb8 samples)? The actin controls seem less intense at the 8h time point and I do not see a normalisation to band strength. A comment on this would be appreciated. Also, this should be quantified.

- It would be nice to have a section in the discussion outlining the relative importance of these findings to human disease and development of new therapies. Are there any trials assessing ROS inhibition in IL-1b dependent disorders like CAPS, SJIA, FMF and others...?.

- Based on Supplementary Figure 1, it would recommend another experiment. CSF-1 promotes survival and differentiation of monocytes. GM-CSF has also been shown to have similar effects. It would be nice to show that CSF-1 has no impact on IL-1b secretion, particularly via the same mechanism. I would recommend running the same experiments as in Figure 1C and 4B with CSF-1, alongside GM-CSF. This would help to rule out survival benefits of exogenous cytokines in the culture systems.

- Figure 2b, an FMO (no reagent added) should be shown as control for each condition in the same histogram. This should be quantified with MFI.

- For all figures, number of samples must be added. In addition, it is not clear whether one experiment is shown or pooled data of several experiments. Individual dots for the replicates should be shown for each experiment.

- For western blots, quantifications need to be added

Referee #3:

The manuscript entitled "GM-CSF suppresses antioxidant signaling through NRF2 downregulation, driving IL-1β secretion" by Carlo et al has used Bone marrow derived monocytic macrophages (BMDMM) and HoxB8 differentiated to monocytic macrophages as model systems to decipher the underlying mechanism of IL-1β secretion reported earlier by the monocytic macrophages in BMDC cultures. The study has shown secretion of IL-1β upon GM-CSF treatment to LPS primed monocytic macrophages. The IL-1β secretion was dependent on components of inflammasome signalling such as Asc, NLRP3, Caspase-1 and GSDMD indicating the role of caspase-1 signalling during GM-CSF mediated IL-1β release. The study goes on to show that GM-CSF mediated IL-1β secretion was dependent on ROS, wherein GM-CSF inhibits the antioxidant signalling via downregulation of NRF2 at mRNA levels as well as affecting the protein degradation. ROS signalling in turn was dependent on LPS-mediated TNF upregulation. The authors finally show that ROS results in ubiquitylation of IL-1β at K133, which may result in its cleavage and secretion.
Therefore, the manuscript provides a link between GM-CSF and regulation of oxidative stress, which in turn can regulate IL-1β maturation and secretion.
The experiments performed are conclusive and augment the scientific conclusions made in the manuscript. However, I have the

following suggestions and concerns:

- Addition of GM-CSF to LPS primed cells results in IL-1β secretion (Fig 1A) and production of pro-IL -1β in cells over and above only LPS control (Fig 1B). The authors can include only GM-CSF controls without LPS priming in both these observations. It is important to show only GM-CSF controls, as GM-CSF is used for differentiation of these cells in the first place, and to see the effect of GM-CSF on IL-1β production and secretion without LPS priming.

- Although, the authors have cited a paper, it is still important to show the transcriptional upregulation of IL-1β upon GM-CSF addition in this scenario. Since, the later section of manuscript points towards the post-translational modification of IL-1β mediated by GM-CSF, it is worthy to see the effect of GM-CSF on the transcription of IL-1β as there are higher levels of pro-IL-1β in GM-CSF treated cells than LPS alone. Is it due to transcriptional upregulation or the post translation modifications and protein stability?

- In figure 1C, authors have shown the secretion of GM-CSF mediated IL-1β to de dependent on Asc, NLRP3 and Caspase-1. However, the production of GM-CSF mediated pro-IL-1β is not dependent on these players. I would expect higher levels of pro-IL-1β than WT in all these cases as there is no IL-1β secretion. A densitometric analysis of the pro-IL-1β blot in this case will be helpful for the readers to appreciate the pro-IL1- β levels upon GM-CSF treatment and in case where there is no secretion.

- In fig 1C, the authors can consider validating the KO lines using western blots as a supplementary information.

- In Fig 3C, the secreted IL-1β levels on LPS+TNF and LPS+GM-CSF+TNF are not statistically significant. However, the authors have concluded in the text that GM-CSF enhances the secretion of IL-1 β in this case. The text "Addition of TNF and LPS alone ...., but this could be further enhanced again by GM-CSF addition........ (Figure 3C)". I think such conclusion is not experimentally validated in the figure.

- In fig 3B and 3C, the levels of TNF produced/secreted is approximately 7.5 to 10 ng/mL upon LPS or LPS+GM-CSF treatment. However, the authors have used 100 ng/mL of rTNF in figure 3C. The effect of GM-CSF on IL-1β production vs LPS alone may be much more prominent if the authors use 10 ng/mL of TNF, which seems physiological in this case rather than a saturating concentration of 100 ng/mL for the experiment.

- The authors conclude that it is TNF-mediated ROS induction which leads to GM-CSF mediated IL-1β secretion. An experiment with rTNF and mDIVI on TNF knock-out cells with controls will be much more conclusive way of saying the same.

- The authors have not supported the text "GM-CSF treated cells did not show increased ROS staining" with any experimental results.

- In supplementary figure 4, the authors have concluded that GM-CSF regulates the protein stability of NRF2 and despite lower mRNA levels of NRF2 post GM-CSF treatment; it is the proteasomal degradation of NRF2 that is higher in case of GM-CSF treatment. However, the results provided do not give proper explanation of the conclusion made. In fig supp 4B, there is lower protein levels of NRF2 upon GM-CSF treatment in comparison to LPS alone, this could be because of lower mRNA levels as shown in fig supp 4A. Upon addition of MG-132, there are higher NRF2 levels in LPS alone treatment, and there are higher levels of NRF2 in cells treated with LPS+GM-CSF. Although, this could entirely be due to accumulation of NRF2 since there is no degradation happening and the lower levels are entirely due to lower mRNA levels. The authors should provide a kinetic experiment using cycloheximide to show higher turnover of NRF2 in GM-CSF treated cells vs LPS alone treatment, to support the conclusions.

- The western blot in figure 5C looks very white for Caspase-1 knock-out. Can authors provide any other representative blot?

- Is there any published report to support the statement "To determine the trigger for ubiquitylation of IL-1β we reasoned that ROS, which are required ....role in IL-1β ubiquitylation"?

- Fig 6 shows that GM-CSF mediated ubiquitylation of IL-1β at K133 and its secretion is dependent on ROS. However, there is no increased ROS experimentally observed in GM-CSF treated cells. The downregulation of NRF2 may result in increased ROS, but that was not observed experimentally. Can the authors comment on this enigma?

- Finally, the GM-CSF mediated IL-1β secretion is dependent of K133 ubiquitylation. Is it possible for the authors to transfect an active form of A20 DUB to the cells to show that deubiquitylation of K133 will result in no IL-1β secretion post GM-CSF treatment?

Minor comment:
- Figure 5B and 5C, is it LPS + GM-CSF + Nigercin or LPS + Nigercin?
- The authors have not cited Figure 5E.

Referee #1:

General Comments: This MS describes novel and significant observations regarding signaling mechanisms by which GM-CSF can synergize with LPS to drive slow but sustained NLRP3/caspase-1-dependent proteolytic processing and non-lytic secretion of IL-1βeta from murine bone marrow-derived monocytic macrophages. The authors present compelling data that supports an atypical pathway for processing and release of IL-1βeta that includes: 1) LPS induced production of autocrine TNFa; 2) TNFa induction of mitochondrial ROS production; 3) GM-CSF-mediated suppression of the LPS-driven up-regulation of the Nrf2 master anti-oxidant protein; 4) ROS-dependent potentiation of an NLRP3/ASC/caspase-1 signaling axis which does NOT involve stable assembly of large NLRP3/ASC inflammasome signaling platforms; 5) sufficient caspase-1 activity to drive the proteolytic processing of proIL-1βeta to mature IL-1β; 6) a GM-CSF-dependent increase in K63-ubiquitination of proIL-1βeta which further enhances its proteolytic processing by caspase-1; 7) an atypical pathway for secretion/ release of the mature IL-1βeta that is dependent on Gasdermin D (GSDMD) but apparently independent of GSDMD-proteolytic cleavage, GSDMD-pore-driven plasma membrane permeabilization and consequent pyroptosis. In general, the experiments are well-designed and thoughtfully interpreted. However, some additional experiments and discussion related to the atypical mechanism(s) underlying the observed IL-1βeta secretion are required. A few other technical issues require clarification.

We thank you for your positive response and criticisms. Please see below for our reply to your concerns.

Specific Comments:

1. The authors cite Monteleone et al 2018 as background for how GSDMD pores can mediate slow IL-1βeta release in the absence of pyroptotic cell death. However, the SyTox green uptake experiments (Fig 3D) indicate that no plasma membrane pores accumulate during LPS/GM-CSF stimulation of the HoxB8 macrophages. If GSDMD pores accumulated even transiently in low numbers and are countered by robust membrane repair, the SyTox influx through the non-lytic pores will intercalate with cellular DNA and irreversibly (on the time scale of these experiments) mark those cells with fluorescent SyTox/DNA complexes. Thus, IL-1βeta efflux through non-lytic plasma membrane GSDMD pores is an unlikely mechanism. Rather, it seems that GSDMD is regulating an alternative IL-1β release that may involve trafficking to a vesicular export pathway (exosomes? secretory autophagosomes?) in these bone marrow monocyte-derived macrophages models. Several recent studies have described such pathways in other myeloid or non-myeloid cell models (Karmakar et al. 2020. PMID32371889; Kimura et al. 2017, PMID 27932448). The authors should evaluate whether some of these alternative pathways are involved in the observed IL-1βeta secretion in their experiments. This is an important mechanistic question.

We thank the reviewer for this helpful comment. We must apologise as it seem we mislabelled the figure legend for the sytox green assays shown. In these assays the sytox green was in fact added after the LPS treatment and was thus not present throughout the assay time. To answer this question however, we have redone the experiments with the sytox green present throughout the time course. Supporting the reviewer's previous conclusion, there is no evidence of significant uptake of sytox green, and no differences between LPS and LPS + GM-CSF could be detected, arguing against there being transient pores of gasdermin D opening up. We further confirmed this by using PEG co-incubation throughout the experiment. Addition of different sizes of PEG can prevent leakage of cytoplasmic contents from plasma membrane pores and was shown to prevent IL-1β release previously. Addition of three different sizes of PEG could not prevent secretion of IL-1β, again arguing against there being a pore involved in its release (see new figure 8).

To address the idea that alternative pathways may be involved, we used confocal microscopy to analyse co-localization of IL-1β with either LAMP2 or LC3 as markers of autophagic vesicles or lysosomes, both of which were proposed to traffic IL-1β out of the cell as indicated in the references mentioned by the reviewer. We could detect some co-localization of LAMP2 with IL-1β, however this was relatively minor. There was a significantly better co-localizaiton with LC3 suggesting that IL-1β may travel through autophagic vesicles for secretion. To determine if autophagy was required for IL-1β release, we differentiated ATG-/- HoxB8 cells derived from ATG5-/- foetal liver. Interestingly, loss of ATG5 resulted in an increase of IL-1β secretion, both with LPS alone and also with LPS+GM-CSF. This argues against an autophagy dependent secretory pathway (see new figure 6).
Of not however, was the localization of IL-1β was mostly in larger longer structures with the LC3 decorating the IL-1β positive area, rather than IL-1β being inside or on LC3 vesicles. These longer structures are similar to those reported to be membrane ruffles in Monteleone etal (1). To confirm this, the cells were also stained with Phalloidin to stain filamentous actin which is responsible for the ruffle formation. There was a strong correlation of Phalloidin and IL-1β. LC3 vesicles could also be seen accumulating in the ruffle regions. This coupled with the increased secretion in ATG5-/- cells may suggest that IL-1β is rather being targeted for degradation by autophagy rather than IL-1β requiring it for secretion. This could explain the low level of co-localization with LAMP2, being the endpoint of autophagic degradation.
Given the large and complex nature of membrane ruffles it may also simply be co-incidental co-localization to these regions. The data do however fit with previous studies showing loss of ATG16L1 leads to spontaneous secretion of IL-1β upon LPS stimulation (2).

We cannot confirm how IL-1β gets to the Ruffles, however the study by Monteleone revealed that once at the ruffles IL-1β could be secreted by both gasdermin D dependent and Independent mechanisms. It seem in the model we have identified the Gasdermin D dependent pathway is dominant. In this study they propose that IL-1β must be cleaved to enrich it at the plasma membrane. Given the large amount detected in the ruffles in HoxB8 macrophages, but the relative low amount of processed IL-1β detected in the cell lysates, this would suggest that cleavage is not a pre-requisite for Ruffle localization, and suggest that cleavage may occur within these ruffle regions themselves. We now propose that IL-1β can be recruited to ruffle regions, probably through the polybasic region identified by monteleone etal. Confocal images of gasdermin D show a speckled pattern possibly reflecting vesicle localization. Gasdermin D was previously reported to promote il1b ubiquitylation (3) and we could confirm that loss of gasdermin D also strongly reduced IL-1β ubiquitylation (see new figure 8). We thus propose a model whereby IL-1β is trafficked to membrane ruffles, by an unknown route. GasderminD may promote IL-1β traffic or secretion or both, possibly through regulating its ubiquitylation.

2.Fig 4: The authors should indicate whether exogenous TNFalpha "rescues" the LPS-induced accumulation of Nrf2 in the TNF-knockout macrophages.
We have now treated the TNF-/- cells with TNF and showed an induction of NRF2 by western blot (see new figure 4). The induction was not statistically significant however. This may be due to the timing of when we have analysed the levels of NRF2. There is clearly an effect of exogenous TNF on IL-1β production as seen in figure 3C and this could be prevented by mDIVI. We do not know however how early TNF is inducing ROS and therefore NRF2 or for how long NRF2 remains induced. We have looked at 12 hours to try and choose a point at which we normally see a difference between the treatments, but it could be that treatment with exogenous TNF induces a more rapid response than with LPS and this is already fading by 12 hours. Due to the time constraints in the revision we could not do extensive time courses to confirm this however.

3. Fig 5E: The authors should discuss the significance of the 35 kDa caspase-1 band observed in non-stimulated cells (lane 2).

We have now discussed this in the results and discussion. The antibody used for figure 5E recognizes the p20 but not the p10 and thus does not recognize the c-terminus, thus it is likely that the 35KDa fragment represents the CARD and Large subunits. Constitutive cleavage within the Inter-domain linker (IDL) is thus likely responsible for this fragment which can be blocked by zVAD. This implies that there is a constitutive activation of caspase-1 in cells. We do not know which receptor is responsible for this low level constitutive activation.

4. Fig. 5: Prolonged treatment of most macrophages with LPS and the zVAD pan-caspase inhibitor induces necroptosis due the suppression of caspase-8 which otherwise restrains RIP1 and RIP3 activation. This should be evaluated/ considered in this experimental model.
We are aware of this issue and had therefor especially titrated zVAD down to a level where no cell death could be detected (10µM). We have now included data to back this up showing that no cell death occurs at 10µM but 50µM (a dose typically used for induction of necroptosis) efficiently kills the cells (See Appendix Figure S4).

Referee #2:

In the current manuscript, the authors describe that GM-CSF induces Nlrp3-dependent Il1b secretion in macrophages in vitro mediated by a ROS-dependent pathway, suppression of Nrf2 and post-translational modifications of Il1b.

The manuscript demonstrates a well constructed series of experiments with an interesting finding. However, several issues need be addressed, and experiments included that would strengthen their claim.
Thank you for your review and positive comments and constructive criticism. Please see below for point by point response to your concerns.

Key points:

Throughout the paper, secreted IL-1β is measured and the implication is that the effects of LPS/GM-CSF and various other reagents added to cell cultures which inhibit IL-1β secretion are, in fact, specific to IL-1β release. The authors should add experiments aimed at de-risking the possibility of a broader inhibition of secretory proteins by these various reagents may be taking place. This is necessary to strengthen the connection between the proposed mechanism and the results provided. To further elaborate with an example, in Figure 2, the authors claim that LPS stimulated HoxB8 macrophages produce mitochondrial ROS, and that these ROS are driving IL-1β secretion.

Small molecules can have many unidentified off target effects. 25 uM is a high concentration for 16h incubation periods and there is not much literature on Mdivi-1, so I recommend strengthening this point with some additional experiments. The awareness to avoid undesired toxicity of small molecules is appreciated, but this is not the only class of off-target effects that can be observed or make interpretation of results challenging.

The authors should show that secretion of other proinflammatory proteins is not inhibited (in your assay set ups) by the compounds used, ideally using a dose response strategy that covers the concentration used in the manuscript (for example, the 25uM MDIVI-1 or 50mM KCl). This should be considered for all compounds used that showed an increase or decrease of IL-1β secretion.

Perhaps a multiplex proinflammatory cytokine/chemokine assay on supernatants from LPS/GM-CSF-stimulated Hoxb8 and BMDMMs to show that indeed the reduction is either specific to, or enhanced for, IL-1β secretion.

Compounds to consider for this experiments include:
CRT0066101
NAP
CCCP
MDIVI-1
Punicalagin
KCl

If a claim is to be made that a mechanism influencing IL-1β secretion is discovered, it should ideally be shown in context of other cytokines and secreted proteins - namely that they are-or-are-not equally affected.

Perhaps the GM-CSF/NRF2 axis is of broader importance? This should be clarified.

We thank the reviewer for this important point. We have now performed a multiplex ELISA (Legendplex – biolegend) on samples from Hoxb8 cells treated with most of the inhibitors and compounds. The results are presented in Figure EV2. For the majority of cytokines, there was no significant increase in secretion in response to GM-CSF, with the exception of IL-6 and MCP-1, which both showed increases. There were mixed results for the effect of inhibitors. For most cytokines the majority of the inhibitors showed no significant alterations in secretion. Punicalagin could only reduce secretion of CXCL10 to some extent, but actually increased secretion of TNF. mDIVI and Mitotempo could only significantly decrease expression of IL-6, but NAC did not. IL-6 is thus the only cytokine tested that somewhat mimics IL-1β in that it is increased by GM-CSF treatment and promoted by mitochondrial ROS production.
We further analysed supernatants from primary cells using the legendplex. The only significant difference detected was the GM-CSF induced IL-1β increase and this was reduced by mitoTEMPO as already reported in the manuscript.
Additionally we have now quantified western blots from samples treated with the various inhibitors and shown that there is no loss of pro-IL-1β production (see new figure EV 1).
We conclude from these results that the various inhibitors are not having global effects on cytokine secretion or IL-1β production at the doses used. The regulation of IL-1β secretion appears to be fairly specific.

Additional comments:

- Figure 4 - Whilst the impact of GM-CSF in NRF2 is clear and a key result in the manuscript is observable.... the "two waves" described in the text relating to Fig 4A seem to be influenced by less protein abundance at the 8h time point (at least in the Hoxb8 samples)? The actin controls seem less intense at the 8h time point and I do not see a normalisation to band strength. A comment on this would be appreciated. Also, this should be quantified.

We agree that the loading controls were not of the best quality. We have re-probed the membranes shown with other loading controls to get better signals and have included these in the modified figure. For the HoxB8 cells, although we consistently see loss of NRF2, the signal gradually comes back and is usually similar to wild type by 16 hours, whereupon il1b secretion plateaus. The timing of this recovery is not exact and in some experiments may occur by 14 and in others at 12 hours for example. Because of this statistical analysis shows rather poor p values, but by 14 hours the Hoxb8 cells become significant. We have now quantified the blots from different time courses and have presented this in figure 4. We have also toned down the statement that there is a strong reduction of NRF2 to reflect this. We hope these changes are satisfactory.

- It would be nice to have a section in the discussion outlining the relative importance of these findings to human disease and development of new therapies. Are there any trials assessing ROS inhibition in IL-1β dependent disorders like CAPS, SJIA, FMF and others...?.

To the best of my knowledge there are no clinical trials testing ROS scavengers in IL-1β dependent disorders. We have however endeavoured to make some more discussion about the relevance of these findings to disease and possible uses for therapeutics strategies.

- Based on Supplementary Figure 1, it would recommend another experiment. CSF-1 promotes survival and differentiation of monocytes. GM-CSF has also been shown to have similar effects. It would be nice to show that CSF-1 has no impact on IL-1β secretion, particularly via the same mechanism. I would recommend running the same experiments as in Figure 1C and 4B with CSF-1, alongside GM-CSF. This would help to rule out survival benefits of exogenous cytokines in the culture systems.

We have now performed experiments using M-CSF (CSF-1) alongside GM-CSF or with GM-CSF alone. There is no induction of IL-1β by LPS + M-CSF, and addition of GM-CSF alone also cannot stimulate IL-1β secretion. See new appendix figure S2.

- Figure 2b, an FMO (no reagent added) should be shown as control for each condition in the same histogram. This should be quantified with MFI.
We have included this in the histograms and have also quantified the results and shown them in the new figure 2. There is a significant reduction in MFI in response to CCCP.

- For all figures, number of samples must be added. In addition, it is not clear whether one experiment is shown or pooled data of several experiments. Individual dots for the replicates should be shown for each experiment.
We have now indicated in all figure legends what the dots represent and shown the individual biological replicates for each analysis.

- For western blots, quantifications need to be added
We have quantified the majority of western blots where a quantitative difference is being shown. For those simply qualitative differences, such as presence of cleaved caspase-1 bands no quantification has been included.

Referee #3:

The manuscript entitled "GM-CSF suppresses antioxidant signaling through NRF2 downregulation, driving IL-1β secretion" by Carlo et al has used Bone marrow derived monocytic macrophages (BMDMM) and HoxB8 differentiated to monocytic macrophages as model systems to decipher the underlying mechanism of IL-1β secretion reported earlier by the monocytic macrophages in BMDC cultures. The study has shown secretion of IL-1β upon GM-CSF treatment to LPS primed monocytic macrophages. The IL-1β secretion was dependent on components of inflammasome signalling such as Asc, NLRP3, Caspase-1 and GSDMD indicating the role of caspase-1 signalling during GM-CSF mediated IL-1β release. The study goes on to show that GM-CSF mediated IL-1β secretion was dependent on ROS, wherein GM-CSF inhibits the antioxidant signalling via downregulation of NRF2 at mRNA levels as well as affecting the protein degradation. ROS signalling in turn was dependent on LPS-mediated TNF upregulation. The authors finally show that ROS results in ubiquitylation of IL-1β at K133, which may result in its cleavage and secretion.
Therefore, the manuscript provides a link between GM-CSF and regulation of oxidative stress, which in turn can regulate IL-1β maturation and secretion.

The experiments performed are conclusive and augment the scientific conclusions made in the manuscript. However, I have the following suggestions and concerns:

Thank you for your review and positive comments and constructive criticism. Please see below for point by point response to your concerns.

- Addition of GM-CSF to LPS primed cells results in IL-1β secretion (Fig 1A) and production of pro-IL -1β in cells over and above only LPS control (Fig 1B). The authors can include only GM-CSF controls without LPS priming in both these observations. It is important to show only GM-CSF controls, as GM-CSF is used for differentiation of these cells in the first place, and to see the effect of GM-CSF on IL-1β production and secretion without LPS priming.
We have now included these controls and as expected, GM-CSF is not sufficient to induce IL-1β secretion on its own (see Appendix figure S2).

- Although, the authors have cited a paper, it is still important to show the transcriptional upregulation of IL-1β upon GM-CSF addition in this scenario. Since, the later section of manuscript points towards the post-translational modification of IL-1β mediated by GM-CSF, it is worthy to see the effect of GM-CSF on the transcription of IL-1β as there are higher levels of pro-IL-1β in GM-CSF treated cells than LPS alone. Is it due to transcriptional upregulation or the post translation modifications and protein stability?
We have now performed qPCR and included it in figure EV 1A and B. GM-CSF leads to a roughly 3.5 fold induction of IL-1β at the mRNA level, but only around 2 fold at the protein level. Quantification of western blots however shows that the increase is not significant. Therefore, GM-CSF can induce IL-1β transcription, but this has a relatively mild effect on protein levels, which cannot explain the large increase in secretion we observe.

- In figure 1C, authors have shown the secretion of GM-CSF mediated IL-1β to be dependent on Asc, NLRP3 and Caspase-1. However, the production of GM-CSF mediated pro-IL-1β is not dependent on these players. I would expect higher levels of pro-IL-1β than WT in all these cases as there is no IL-1β secretion. A densitometric analysis of the pro-IL-1β blot in this case will be helpful for the readers to appreciate the pro-IL1- β levels upon GM-CSF treatment and in case where there is no secretion.
We have now quantified all blots in question and the quantification is included. There is no significant increase in pro-il1b in the various knockouts (Figure EV 1C). This is perhaps not surprising, as a loss of secretion due to knockouts may not necessarily result in detectable increases in intracellular IL-1β over the time periods we use. We see a slow and steady secretion of IL-1β between 8-16 hours. It is not clear what happens to IL-1β that is not secreted. It may be turned over in that time, thus preventing accumulation for example.

- In fig 1C, the authors can consider validating the KO lines using western blots as a supplementary information.
We have now included western blots for all of the knockouts within the relevant figures where they are used. For controlling TNF-/- we used ELISA instead of western blot.

- In Fig 3C, the secreted IL-1β levels on LPS+TNF and LPS+GM-CSF+TNF are not statistically significant. However, the authors have concluded in the text that GM-CSF enhances the secretion of IL-1 β in this case. The text "Addition of TNF and LPS alone ...., but this could be further enhanced again by GM-CSF addition........ (Figure 3C)". I think such conclusion is not experimentally validated in the figure.

We have now repeated this experiment at 50ng/mL. Please see below comment for an explanation of why we have used this concentration now. With this dose, we see a significant difference after addition of GM-CSF. We have additionally used mDIVI here as well and shown that blocking ROS with mDIVI reverses the effect of GM-CSF in the presence of exogenous TNF. TNF without GM-CSF is still

able to induce IL-1β secretion which is not dependent on ROS. This may be due to parallel activation of other inflammasomes by TNF or even caspase-8 dependent cleavage of IL-1β for example.

- In fig 3B and 3C, the levels of TNF produced/secreted is approximately 7.5 to 10 ng/mL upon LPS or LPS+GM-CSF treatment. However, the authors have used 100 ng/mL of rTNF in figure 3C. The effect of GM-CSF on IL-1β production vs LPS alone may be much more prominent if the authors use 10 ng/mL of TNF, which seems physiological in this case rather than a saturating concentration of 100 ng/mL for the experiment.

We have now preformed multiplex ELISA and detected TNF in these same samples using this method. Surprisingly, the amount of TNF detected is much higher than we detected with plate ELISA and corresponds to roughly 50-70ng/mL of TNF. We have reason to suspect the sensitivity of our plate ELISAs for TNF unfortunately, based on some poor quality control from the supplier, and think rather that the multiplex ELISA is more accurate in this case and have replaced the old data in figure 3 with data from the multiplex ELISA.

- The authors conclude that it is TNF-mediated ROS induction which leads to GM-CSF mediated IL-1β secretion. An experiment with rTNF and mDIVI on TNF knock-out cells with controls will be much more conclusive way of saying the same.

Please see the comment above. We have performed this experiment and included it in figure 3. mDIVI was able to reverse the effect of GM-CSF in the presence of recombinant TNF.

- The authors have not supported the text "GM-CSF treated cells did not show increased ROS staining" with any experimental results.
Thank you for pointing this out. We have now included this result in figure 2.

- In supplementary figure 4, the authors have concluded that GM-CSF regulates the protein stability of NRF2 and despite lower mRNA levels of NRF2 post GM-CSF treatment; it is the proteasomal degradation of NRF2 that is higher in case of GM-CSF treatment. However, the results provided do not give proper explanation of the conclusion made. In fig supp 4B, there is lower protein levels of NRF2 upon GM-CSF treatment in comparison to LPS alone, this could be because of lower mRNA levels as shown in fig supp 4A. Upon addition of MG-132, there are higher NRF2 levels in LPS alone treatment, and there are higher levels of NRF2 in cells treated with LPS+GM-CSF. Although, this could entirely be due to accumulation of NRF2 since there is no degradation happening and the lower levels are entirely due to lower mRNA levels. The authors should provide a kinetic experiment using cycloheximide to show higher turnover of NRF2 in GM-CSF treated cells vs LPS alone treatment, to support the conclusions.
We thank the reviewer for this suggestion. We have attempted to address this as the reviewer suggested (please see figure below). What is clear however, is that the half life of NRF2 once stabilized is extremely short (less than 30mins). We have gone down to 30min of CHX treatment and see basically complete loss of any NRF2 present in the cell. It is thus technically very difficult to reliably go much further. NRF2 can be thought of as having two states, with differing half-lifes. The first state basically has a half life of probably seconds, thus leading to net zero protein in the cell (see cartoon figure below). When a signal such as LPS arrives, this leads to stabilization of a sub-population of NRF2 only (supported by our experiments with MG132, showing strong accumulation of NRF2 with MG132, even after LPS treatment), leaving the majority in the first state. Once stabilized it travels to the nucleus, activates target genes, and returns to the cytosol where it is either degraded again, or may re-enter the nucleus (see figure below). This second state of NRF2 is what we detect by western blot and due to recycling into the cytosol, where it is degraded, still has a half-life of only minutes. We propose that GM-CSF reduces the pool of NRF2 that escapes state one and is

stabilized, thus leading to overall lower levels. Given the amount of NRF2 that enters state 2 is generally low relative to the total pool that remains in state 1 (i.e. degradation), the fate of this NRF2 is most likely degradation when it returns to the cytosol. It is likely then that the half-life is effectively the same for NRF2 regardless of addition of GM-CSF or not, but that the total amount of NRF2 that escapes the rapid degradation is smaller with GM-CSF, leading to less NRF2 entering state 2. This is of course a difficult thing to properly analyse and we believe beyond the scope of the revision.

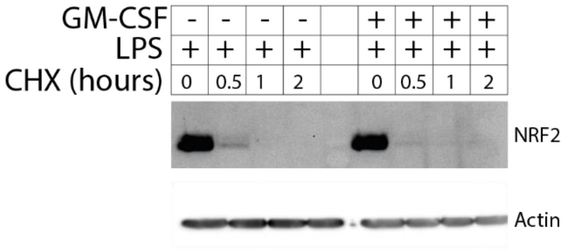

NRF2 is extremely unstable: HoxB8 Cells were primed for 3h using 60ng/ml LPS before addition of 10µM QVD (to block cell death). After 30 minutes of QVD stimulation, 20µg/ml cycloheximide was added for the indicated times. Cells were lysed in 6M urea lysis buffer and ran on a 4-20% SDS-PAGE Tris-glycine gel.

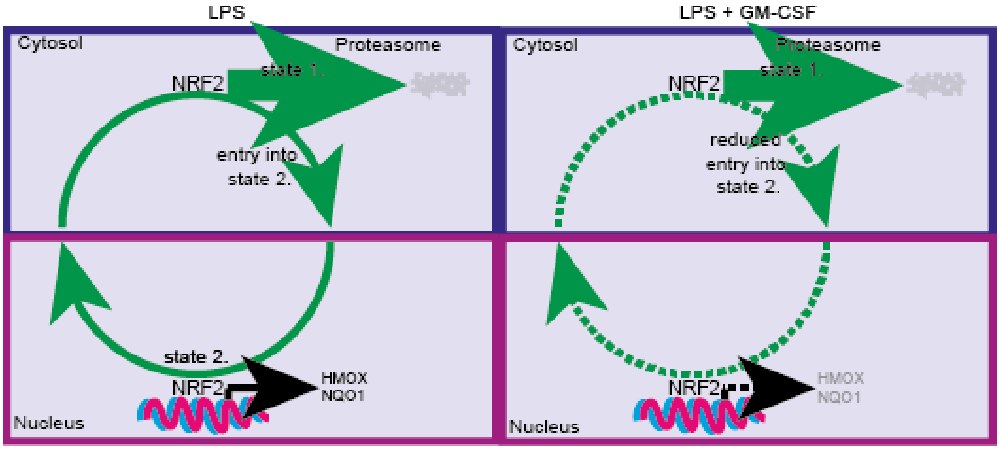

Model for GM-CSf regulation of NRF2: GM-CSF may reduce the amount of NRF2 that is stabilized (state 2), leading to a net reduction in active NRF2.

- The western blot in figure 5C looks very white for Caspase-1 knock-out. Can authors provide any other representative blot?

We have included another exposure with reduced contrast from the same blot.

- Is there any published report to support the statement "To determine the trigger for ubiquitylation of IL-1β we reasoned that ROS, which are required ....role in IL-1β ubiquitylation"?

This statement is rather referring to our own results from the previous figure. To avoid confusion, we have re-worded the sentence to refer back to the relevant figure.

- Fig 6 shows that GM-CSF mediated ubiquitylation of IL-1β at K133 and its secretion is dependent on ROS. However, there is no increased ROS experimentally observed in GM-CSF treated cells. The downregulation of NRF2 may result in increased ROS, but that was not observed experimentally. Can the authors comment on this enigma?

This is indeed an enigma which we dearly wish to resolve and it has not been for want of trying. We have used fluorescent ROS reporters (several different ones), as well as trying to measure glutathione (reduced/oxidized) ratios and staining with biotinylated glutathione to name but a few techniques. What we observed is that in each case, these techniques are rather insensitive. We could consistently detect ROS changes with strong stimuli such as H202 which we used as a positive controls, but other less potent stimuli are not well detected. There are mild redox sensitive modifications of proteins that do not necessarily require strong oxidative stress, such as cysteine sulfenylation which almost certainly occur even if ROS levels are too low to readily detect with reagents such as ROS sensitive Dyes. We believe that this is the case in our model.

- Finally, the GM-CSF mediated IL-1β secretion is dependent of K133 ubiquitylation. Is it possible for the authors to transfect an active form of A20 DUB to the cells to show that deubiquitylation of K133 will result in no IL-1β secretion post GM-CSF treatment?
We have attempted to express A20 in the HoxB8 cells, using both viral transduction and transient transfection, but have not achieved significant overexpression unfortunately. Not surprisingly we could see no changes in IL-1β ubiquitylation in these attempts (data not shown). I would however like to comment that this approach may not be helpful given the strong impact A20 has on both TLR4 and TNF signalling. Overexpression of A20 is known to block TNF induced NF-kb activation for example (4). Given the central role of both TLR4 and TNF signalling in the pathway we are analysing, it is likely that had A20 overexpression worked, we may have simply prevented activation of the pathway upstream by blocking TNF production for example. To further analyse possible roles of A20, we will need to make use of either other cell systems or potentially inducible expression or even trying knock-in mutants using CRISPR, but this is beyond the scope of what we could achieve with this revision.

Minor comment:
- Figure 5B and 5C, is it LPS + GM-CSF + Nigercin or LPS + Nigercin?
Thank you for spotting this. It is in fact LPS + Nigericin, not LPS + GM-CSF + Nigericin. We have corrected the figure now.

- The authors have not cited Figure 5E.
Thank you for pointing this out. We have now included the figure reference in the text.

References

1. Monteleone M, Stanley AC, Chen KW, Brown DL, Bezbradica JS, von Pein JB, Holley CL, Boucher D, Shakespear MR, Kapetanovic R, Rolfes V, Sweet MJ, Stow JL, Schroder K. 2018. Interleukin-1β Maturation Triggers Its Relocation to the Plasma Membrane for Gasdermin-D-Dependent and -Independent Secretion. CellReports 24:1425-1433.
2. Saitoh T, Fujita N, Jang MH, Uematsu S, Yang B-G, Satoh T, Omori H, Noda T, Yamamoto N, Komatsu M, Tanaka K, Kawai T, Tsujimura T, Takeuchi O, Yoshimori T, Akira S. 2008. Loss of the autophagy protein Atg16L1 enhances endotoxin-induced IL-1beta production. Nature 456:264-268.
3. Bulek K, Zhao J, Liao Y, Rana N, Corridoni D, Antanaviciute A, Chen X, Wang H, Qian W, Miller-Little WA, Swaidani S, Tang F, Willard BB, McCrae K, Kang Z, Dubyak GR, Cominelli F, Simmons A, Pizarro TT, Li X. 2020. Epithelial-derived gasdermin D mediates nonlytic IL-1β release during experimental colitis. The Journal of Clinical Investigation 130:4218-4234.
4. Heyninck K, De Valck D, Vanden Berghe W, Van Criekinge W, Contreras R, Fiers W, Haegeman G, Beyaert R. 1999. The zinc finger protein A20 inhibits TNF-induced NF-kappaB-dependent gene expression by interfering with an RIP- or TRAF2-mediated transactivation signal and

directly binds to a novel NF-kappaB-inhibiting protein ABIN. The Journal of Cell Biology 145:1471-1482.

1st Revision - Editorial Decision 12th Apr 2022

Dear Dr. Gentle,

Thank you for the submission of your revised manuscript to our editorial offices. I have now received the reports from the referees that was asked to re-evaluate your study, you will find below. As you will see, the referees now fully support the publication of your work. Referee #3 has 2 remaining minor concerns or suggestions to improve the study I ask you to address in a final revised version of the manuscript. Please also provide a short p-b-p-response to these remaining points.

Moreover, I have these editorial requests:

- I would suggest a modified title (without the comma):
GM-CSF suppresses antioxidant signaling and drives IL-1β secretion through NRF2 downregulation

- Please reduce the number of keywords to five.

- Please provide the abstract written in present tense throughout.

- Please call out the figure panels separately and sequentially. Presently, there seems to be no callout for panel 6A. Please check.

- Please order the manuscript sections like this:
Title page - Abstract - Introduction - Results - Discussion - Materials and Methods - DAS - Acknowledgements - Author contributions - Disclosure and Competing Interests Statement - References - Figure legends - Expanded View Figure legends

- Per journal policy, we do not allow 'data not shown', which is stated three times in the manuscript (pages 9, 12, 14). All data referred to in the paper should be displayed in the main or Expanded View figures, or an Appendix. Thus, please add these data (or change the text accordingly if these data are not central to the study). See:
http://embor.embopress.org/authorguide#unpublisheddata

- We updated our journal's competing interests policy in January 2022 and request authors to consider both actual and perceived competing interests. Please review the policy https://www.embopress.org/competing-interests and update your competing interests if necessary. Please name this section 'Disclosure and Competing Interests Statement' and put it after the Acknowledgements section.

- Please make sure that the number "n" for how many independent experiments were performed, their nature (biological versus technical replicates), the bars and error bars (e.g. SEM, SD) and the test used to calculate p-values is indicated in the respective figure legends (main, EV and Appendix figures), and that statistical testing has been done where applicable. Please avoid phrases like 'independent experiment', but clearly state if these were biological or technical replicates. Please add complete statistical testing to all diagrams (main, EV and Appendix figures). Please also indicate (e.g. with n.s.) if testing was performed, but the differences are not significant. Presently, several diagrams have no statistics (e.g. 1D, 2D, 4F, 7C and mot panels of EV1) or only partial statistics (4A, 4E, 4D, 8C and those in EV2).

- Please add scale bars of similar style and thickness to the microscopic images (main, EV and Appendix figures), using clearly visible black or white bars (depending on the background). Please place these in the lower right corner of the images. Please do not write on or near the bars in the image but define the size in the respective figure legend. Presently, some panels are missing scale bars, or they are positioned wrongly or are too thin or contain text.

- There is a diagram in Fig. 3 below panel 3A. It is not clear where this belongs to, and where its legend is. Please check.

- As there are only few panels in the EV and Appendix images. I would therefore suggest combining these to have 5 EV figures containing all these data. In that case, we would need no Appendix. Please consider this. In case please update the figure/panel callouts. If you decide to keep an Appendix, please put the legends below the figures. That is much more comprehensible. For all figures, please order the panels alphabetically (presently, in Fig. EV the order is A,B,D,E,F,C). Please consult our guide for figure preparation:
http://wol-prod-cdn.literatumonline.com/pb-assets/embo-site/EMBOPress_Figure_Guidelines_061115-1561436025777.pdf

- As they are partially significantly cropped, please provide the source data for all the Western blots shown in the manuscript (main and EV figures). The source data will be published in separate source data files online along with the accepted manuscript and will be linked to the relevant figures. Please submit scans of entire gels or blots together with the final revised manuscript. Please include size markers for scans of entire gels, label the scans with figure and panel number, and send one PDF file per figure.

- Please provide your funding information in the acknowledgements and make sure that all the funding information is also

entered into the online submission system and that it is complete and similar to the one in the acknowledgement section of the manuscript text file.

- The synopsis image provided has the wrong dimensions. Please upload this in jpeg or tiff format with the exact width of 550 pixels and a height of not more than 400 pixels. Please make sure that after reformatting the writing and details are not too small.

- Finally, please find attached a word file of the manuscript text (provided by our publisher) with changes we ask you to include in your final manuscript text, and some queries, we ask you to address. Please provide your final manuscript file with track changes, in order that we can see any modifications done.

I look forward to seeing the final revised version of your manuscript when it is ready. Please let me know if you have questions regarding the revision.

Please use this link to submit your revision: https://embor.msubmit.net/cgi-bin/main.plex

Best,

Achim Breiling
Senior Editor
EMBO Reports

Referee #1:

General Comments: This MS describes novel and significant observations regarding signaling mechanisms by which GM-CSF can synergize with LPS to drive slow but sustained NLRP3/caspase-1-dependent proteolytic processing and non-lytic secretion of IL-1beta from murine bone marrow-derived monocytic macrophages. The authors present compelling data that supports an atypical pathway for processing and release of IL-1beta that includes: 1) LPS induced production of autocrine TNFa; 2) TNFa induction of mitochondrial ROS production; 3) GM-CSF-mediated suppression of the LPS-driven up-regulation of the Nrf2 master anti-oxidant protein; 4) ROS-dependent potentiation of an NLRP3/ASC/caspase-1 signaling axis which does NOT involve stable assembly of large NLRP3/ASC inflammasome signaling platforms; 5) sufficient caspase-1 activity to drive the proteolytic processing of proIL-1beta to mature IL-1b; 6) a GM-CSF-dependent increase in K63-ubiquitination of proIL-1beta which further enhances its proteolytic processing by caspase-1; 7) an atypical pathway for secretion/ release of the mature IL-1beta that is dependent on Gasdermin D (GSDMD) but apparently independent of GSDMD-proteolytic cleavage, GSDMD-pore-driven plasma membrane permeabilization and consequent pyroptosis. In general, the experiments are well-designed and thoughtfully interpreted.

In this revised MS, the authors have thoroughly responded to the concerns and recommendations of the reviewers with new experiments and modification of the text.

Referee #2:

The manuscript is suitable for publication in EMBO reports without further revision.

Referee #3:

The manuscript entitled "GM-CSF suppresses antioxidant signaling through NRF2 downregulation, driving IL-1β secretion" by Carlo et al has used Bone marrow derived monocytic macrophages (BMDMM) and HoxB8 differentiated to monocytic macrophages as model systems to decipher the underlying mechanism of IL-1β secretion reported earlier by the monocytic macrophages in BMDC cultures. The study has shown secretion of IL-1β upon GM-CSF treatment to LPS primed monocytic macrophages. The IL-1β secretion was dependent on components of inflammasome signalling such as Asc, NLRP3, Caspase-1 and GSDMD indicating the role of caspase-1 signalling during GM-CSF mediated IL-1β release. The study goes on to show that GM-CSF mediated IL-1β secretion was dependent on ROS, wherein GM-CSF inhibits the antioxidant signalling via downregulation of NRF2 at mRNA levels as well as affecting the protein degradation. ROS signalling in turn was dependent on LPS-mediated TNF upregulation. The authors finally show that ROS results in ubiquitylation of IL-1β at K133, which may result in its cleavage and secretion. Therefore, the manuscript provides a link between GM-CSF and regulation of oxidative stress, which in turn can regulate IL-1β maturation and secretion.

I thank the authors for their responses to the queries. The responses were comprehensive and answered my curiosities and queries regarding the study.

However, I have a few minor comments as follows:
- In Figure 1B: the IL-1β secretion is dependent on dose of GM-CSF, except for 2 ng/mL concentration, where the IL-1β levels are less than that observed for 1.6 ng/mL. However, the western blot performed on the supernatant does not depict similar results. Can authors investigate this discrepancy?
- Figure 8E: legends for the western blot missing.

Referee #3:

The manuscript entitled "GM-CSF suppresses antioxidant signaling through NRF2 downregulation, driving IL-1β secretion" by Carlo et al has used Bone marrow derived monocytic macrophages (BMDMM) and HoxB8 differentiated to monocytic macrophages as model systems to decipher the underlying mechanism of IL-1β secretion reported earlier by the monocytic macrophages in BMDC cultures. The study has shown secretion of IL-1β upon GM-CSF treatment to LPS primed monocytic macrophages. The IL-1β secretion was dependent on components of inflammasome signalling such as Asc, NLRP3, Caspase-1 and GSDMD indicating the role of caspase-1 signalling during GM-CSF mediated IL-1β release. The study goes on to show that GM-CSF mediated IL-1β secretion was dependent on ROS, wherein GM-CSF inhibits the antioxidant signalling via downregulation of NRF2 at mRNA levels as well as affecting the protein degradation. ROS signalling in turn was dependent on LPS-mediated TNF upregulation. The authors finally show that ROS results in ubiquitylation of IL-1β at K133, which may result in its cleavage and secretion. Therefore, the manuscript provides a link between GM-CSF and regulation of oxidative stress, which in turn can regulate IL-1β maturation and secretion.

I thank the authors for their responses to the queries. The responses were comprehensive and answered my curiosities and queries regarding the study.

However, I have a few minor comments as follows:
- In Figure 1B: the IL-1β secretion is dependent on dose of GM-CSF, except for 2 ng/mL concentration, where the IL-1β levels are less than that observed for 1.6 ng/mL. However, the western blot performed on the supernatant does not depict similar results. Can authors investigate this discrepancy?

We thank the reviewer for their positive response and for pointing out this issue. It seems that during figure preparation there was a mislabelling of the ELISA data, so that the 2ng/mL was actually the LPS alone treated sample, thus the lowered secretion. We have now adjusted the graph to reflect the correct labelling and corresponding western blot.

- Figure 8E: legends for the western blot missing.

Again, thank you for spotting this. We have now included a figure legend for figure 8E.

Dr. Ian Gentle
University Medical Centre Freiburg
Medical Microbiology and Hygiene
Hermann-Herder.-Str. 11
Freiburg 79104
Germany

Dear Dr. Gentle,

I am very pleased to accept your manuscript for publication in the next available issue of EMBO reports. Thank you for your contribution to our journal.

At the end of this email I include important information about how to proceed. Please ensure that you take the time to read the information and complete and return the necessary forms to allow us to publish your manuscript as quickly as possible.

As part of the EMBO publication's Transparent Editorial Process, EMBO reports publishes online a Review Process File to accompany accepted manuscripts. As you are aware, this File will be published in conjunction with your paper and will include the referee reports, your point-by-point response and all pertinent correspondence relating to the manuscript.

If you do NOT want this File to be published, please inform the editorial office within 2 days, if you have not done so already, otherwise the File will be published by default [contact: emboreports@embo.org]. If you do opt out, the Review Process File link will point to the following statement: "No Review Process File is available with this article, as the authors have chosen not to make the review process public in this case."

Should you be planning a Press Release on your article, please get in contact with emboreports@wiley.com as early as possible, in order to coordinate publication and release dates.

Thank you again for your contribution to EMBO reports and congratulations on a successful publication. Please consider us again in the future for your most exciting work.

Yours sincerely,

Achim Breiling
Editor
EMBO Reports

****

THINGS TO DO NOW:

Please note that you will be contacted by Wiley Author Services to complete licensing and payment information. The required 'Page Charges Authorization Form' is available here: https://www.embopress.org/pb-assets/embo-site/er_apc.pdf

You will receive proofs by e-mail approximately 2-3 weeks after all relevant files have been sent to our Production Office; you should return your corrections within 2 days of receiving the proofs.

Please inform us if there is likely to be any difficulty in reaching you at the above address at that time. Failure to meet our deadlines may result in a delay of publication, or publication without your corrections.

All further communications concerning your paper should quote reference number EMBOR-2021-54226V3 and be addressed to emboreports@wiley.com.

Should you be planning a Press Release on your article, please get in contact with emboreports@wiley.com as early as possible, in order to coordinate publication and release dates.

EMBO Press Author Checklist

Corresponding Author Name: Ian Gentle	
Journal Submitted to: EMBO Reports	
Manuscript Number: EMBOR-2021-54226	

USEFUL LINKS FOR COMPLETING THIS FORM
The EMBO Journal - Author Guidelines
EMBO Reports - Author Guidelines
Molecular Systems Biology - Author Guidelines
EMBO Molecular Medicine - Author Guidelines

Reporting Checklist for Life Science Articles (updated January 2022)
This checklist is adapted from Materials Design Analysis Reporting (MDAR) Checklist for Authors. MDAR establishes a minimum set of requirements in transparent reporting in the life sciences (see Statement of Task: 10.31222/osf.io/9sm4x). Please follow the journal's guidelines in preparing your manuscript.
Please note that a copy of this checklist will be published alongside your article.

Abridged guidelines for figures
1. Data
The data shown in figures should satisfy the following conditions:
- the data were obtained and processed according to the field's best practice and are presented to reflect the results of the experiments in an accurate and unbiased manner.
- ideally, figure panels should include only measurements that are directly comparable to each other and obtained with the same assay.
- plots include clearly labeled error bars for independent experiments and sample sizes. Unless justified, error bars should not be shown for technical replicates.
- if n<5, the individual data points from each experiment should be plotted. Any statistical test employed should be justified.
- Source Data should be included to report the data underlying figures according to the guidelines set out in the authorship guidelines on Data Presentation.

2. Captions
Each figure caption should contain the following information, for each panel where they are relevant:
- a specification of the experimental system investigated (eg cell line, species name).
- the assay(s) and method(s) used to carry out the reported observations and measurements.
- an explicit mention of the biological and chemical entity(ies) that are being measured.
- an explicit mention of the biological and chemical entity(ies) that are altered/varied/perturbed in a controlled manner.
- the exact sample size (n) for each experimental group/condition, given as a number, not a range;
- a description of the sample collection allowing the reader to understand whether the samples represent technical or biological replicates (including how many animals, litters, cultures, etc.).
- a statement of how many times the experiment shown was independently replicated in the laboratory.
- definitions of statistical methods and measures:
  - common tests, such as t-test (please specify whether paired vs. unpaired), simple χ2 tests, Wilcoxon and Mann-Whitney tests, can be unambiguously identified by name only, but more complex techniques should be described in the methods section;
  - are tests one-sided or two-sided?
  - are there adjustments for multiple comparisons?
  - exact statistical test results, e.g., P values = x but not P values < x;
  - definition of 'center values' as median or average;
  - definition of error bars as s.d. or s.e.m.

Please complete ALL of the questions below.
Select "Not Applicable" only when the requested information is not relevant for your study.

Materials

Newly Created Materials	Information included in the manuscript?	In which section is the information available? (Reagents and Tools Table, Materials and Methods, Figures, Data Availability Section)
New materials and reagents need to be available; do any restrictions apply?	Yes	No

Antibodies	Information included in the manuscript?	In which section is the information available? (Reagents and Tools Table, Materials and Methods, Figures, Data Availability Section)
For **antibodies** provide the following information:<br>- Commercial antibodies: RRID (if possible) or supplier name, catalogue number and or/clone number<br>- Non-commercial: RRID or citation	Yes	Material and Methods

DNA and RNA sequences	Information included in the manuscript?	In which section is the information available? (Reagents and Tools Table, Materials and Methods, Figures, Data Availability Section)
**Short novel DNA or RNA including primers, probes**: provide the sequences.	Not Applicable	

Cell materials	Information included in the manuscript?	In which section is the information available? (Reagents and Tools Table, Materials and Methods, Figures, Data Availability Section)
**Cell lines:** Provide species information, strain. Provide accession number in repository **OR** supplier name, catalog number, clone number, and/**OR** RRID.	Yes	Material and Methods
**Primary cultures:** Provide species, strain, sex of origin, genetic modification status.	Yes	Material and Methods
Report if the cell lines were recently **authenticated** (e.g., by STR profiling) and tested for mycoplasma contamination.	Yes	Tested for Mycoplasma - Negative

Experimental animals	Information included in the manuscript?	In which section is the information available? (Reagents and Tools Table, Materials and Methods, Figures, Data Availability Section)
**Laboratory animals or Model organisms:** Provide species, strain, sex, age, genetic modification status. Provide accession number in repository **OR** supplier name, catalog number, clone number, **OR** RRID.	Not Applicable	
**Animal observed in or captured from the field:** Provide species, sex, and age where possible.	Not Applicable	
Please detail **housing and husbandry conditions**.	Not Applicable	

Plants and microbes	Information included in the manuscript?	In which section is the information available? (Reagents and Tools Table, Materials and Methods, Figures, Data Availability Section)
**Plants:** provide species and strain, ecotype and cultivar where relevant, unique accession number if available, and source (including location for collected wild specimens).	Not Applicable	
**Microbes:** provide species and strain, unique accession number if available, and source.	Not Applicable	

Human research participants	Information included in the manuscript?	In which section is the information available? (Reagents and Tools Table, Materials and Methods, Figures, Data Availability Section)
If collected and within the bounds of privacy constraints report on age, sex and gender or ethnicity for all study participants.	Not Applicable	

Core facilities	Information included in the manuscript?	In which section is the information available? (Reagents and Tools Table, Materials and Methods, Figures, Data Availability Section)
If your work benefited from core facilities, was their service mentioned in the acknowledgments section?	Yes	Acknowledgments

Design

Study protocol	Information included in the manuscript?	In which section is the information available? (Reagents and Tools Table, Materials and Methods, Figures, Data Availability Section)
If study protocol has been **pre-registered, provide DOI in the manuscript**. For clinical trials, provide the trial registration number **OR** cite DOI.	Not Applicable	
Report the **clinical trial registration number** (at ClinicalTrials.gov or equivalent), where applicable.	Not Applicable	

Laboratory protocol	Information included in the manuscript?	In which section is the information available? (Reagents and Tools Table, Materials and Methods, Figures, Data Availability Section)
Provide DOI OR other citation details if **external detailed step-by-step protocols** are available.	Not Applicable	

Experimental study design and statistics	Information included in the manuscript?	In which section is the information available? (Reagents and Tools Table, Materials and Methods, Figures, Data Availability Section)
Include a statement about **sample size** estimate even if no statistical methods were used.	Yes	Figures
Were any steps taken to minimize the effects of subjective bias when allocating animals/samples to treatment (e.g. **randomization procedure**)? If yes, have they been described?	Not Applicable	No
Include a statement about **blinding** even if no blinding was done.	Not Applicable	No blinding was done
Describe **inclusion/exclusion criteria** if samples or animals were excluded from the analysis. Were the criteria pre-established?<br><br>If sample or data points were omitted from analysis, report if this was due to attrition or intentional exclusion and provide justification.	Not Applicable	
For every figure, are **statistical tests** justified as appropriate? Do the data meet the assumptions of the tests (e.g., normal distribution)? Describe any methods used to assess it. Is there an estimate of variation within each group of data? Is the variance similar between the groups that are being statistically compared?	Yes	Figures and Methods

Sample definition and in-laboratory replication	Information included in the manuscript?	In which section is the information available? (Reagents and Tools Table, Materials and Methods, Figures, Data Availability Section)
In the figure legends: state number of times the experiment was **replicated** in laboratory.	Yes	Figures
In the figure legends: define whether data describe **technical or biological replicates**.	Yes	Figures

Ethics

Ethics	Information included in the manuscript?	In which section is the information available? (Reagents and Tools Table, Materials and Methods, Figures, Data Availability Section)
Studies involving **human participants**: State details of **authority granting ethics approval** (IRB or equivalent committee(s), provide reference number for approval.	Not Applicable	
Studies involving **human participants**: Include a statement confirming that **informed consent** was obtained from all subjects and that the experiments conformed to the principles set out in the WMA Declaration of Helsinki and the Department of Health and Human Services Belmont Report.	Not Applicable	
Studies involving **human participants:** For publication of **patient photos**, include a statement confirming that consent to publish was obtained.	Not Applicable	
Studies involving experimental **animals**: State details of **authority granting ethics approva**l (IRB or equivalent committee(s), provide reference number for approval. Include a statement of compliance with ethical regulations.	Not Applicable	
Studies involving **specimen and field samples:** State if relevant **permits** obtained, provide details of authority approving study; if none were required, explain why.	Not Applicable	

Dual Use Research of Concern (DURC)	Information included in the manuscript?	In which section is the information available? (Reagents and Tools Table, Materials and Methods, Figures, Data Availability Section)
Could your study fall under dual use research restrictions? Please check biosecurity documents and list of **select agents and toxins** (CDC): https://www.selectagents.gov/sat/list.htm	Not Applicable	
If you used a select agent, is the security level of the lab appropriate and reported in the manuscript?	Not Applicable	
If a study is subject to dual use research of concern regulations, is the name of the **authority granting approval and reference number** for the regulatory approval provided in the manuscript?	Not Applicable	

Reporting

The MDAR framework recommends adoption of discipline-specific guidelines, established and endorsed through community initiatives. Journals have their own policy about requiring specific guidelines and recommendations to complement MDAR.

Adherence to community standards	Information included in the manuscript?	In which section is the information available? (Reagents and Tools Table, Materials and Methods, Figures, Data Availability Section)
State if relevant guidelines or checklists (e.g., **ICMJE, MIBBI, ARRIVE, PRISMA**) have been followed or provided.	Not Applicable	
For **tumor marker prognostic studies,** we recommend that you follow the **REMARK** reporting guidelines (see link list at top right). See author guidelines, under 'Reporting Guidelines'. Please confirm you have followed these guidelines.	Not Applicable	
For **phase II and III randomized controlled trials**, please refer to the **CONSORT** flow diagram (see link list at top right) and submit the CONSORT checklist (see link list at top right) with your submission. See author guidelines, under 'Reporting Guidelines'. Please confirm you have submitted this list.	Not Applicable	

Data Availability

Data availability	Information included in the manuscript?	In which section is the information available? (Reagents and Tools Table, Materials and Methods, Figures, Data Availability Section)
Have **primary datasets** been deposited according to the journal's guidelines (see 'Data Deposition' section) and the respective accession numbers provided in the Data Availability Section?	Not Applicable	
Were **human clinical and genomic datasets** deposited in a public access-controlled repository in accordance to ethical obligations to the patients and to the applicable consent agreement?	Not Applicable	
Are **computational models** that are central and integral to a study available without restrictions in a machine-readable form? Were the relevant accession numbers or links provided?	Not Applicable	
If publicly available data were reused, provide the respective **data citations in the reference list**.	Not Applicable	
